# HIV-Specific Reported Outcome Measures: Systematic Review of Psychometric Properties

**DOI:** 10.2196/39015

**Published:** 2022-12-08

**Authors:** Ziqi Wang, Yaxin Zhu, Xiyu Duan, Hao Kang, Bo Qu

**Affiliations:** 1 Institute for International Health Professions Education and Research China Medical University Shenyang China; 2 Administration Department of Nosocomial Infection Southwest Hospital Third Military Medical University Chongqing China; 3 School of Public Health China Medical University Shenyang China

**Keywords:** HIV, AIDS, people living with HIV and AIDS, patient-reported outcome measures, psychometric properties

## Abstract

**Background:**

The management of people living with HIV and AIDS is multidimensional and complex. Using patient-reported outcome measures (PROMs) has been increasingly recognized to be the key factor for providing patient-centered health care to meet the lifelong needs of people living with HIV and AIDS from diagnosis to death. However, there is currently no consensus on a PROM recommended for health care providers and researchers to assess health outcomes in people living with HIV and AIDS.

**Objective:**

The purpose of this systematic review was to summarize and categorize the available validated HIV-specific PROMs in adults living with HIV and AIDS and to assess these PROMs using the Consensus-Based Standards for the Selection of Health Measurement Instruments (COSMIN) methodology.

**Methods:**

This systematic review followed the PRISMA (Preferred Reporting Items for Systematic Reviews and Meta-Analyses) guidelines. A literature search of 3 recommended databases (PubMed, Embase, and PsychINFO) was conducted on January 15, 2021. Studies were included if they assessed any psychometric property of HIV-specific PROMs in adults living with HIV and AIDS and met the eligibility criteria. The PROMs were assessed for 9 psychometric properties, evaluated in each included study following the COSMIN methodology by assessing the following: the methodological quality assessed using the COSMIN risk of bias checklist; overall rating of results; level of evidence assessed using the modified Grading of Recommendations, Assessment, Development, and Evaluation approach; and level of recommendation.

**Results:**

A total of 88 PROMs classified into 8 categories, assessing the psychometric properties of PROMs for adults living with HIV and AIDS, were identified in 152 studies including 79,213 people living with HIV and AIDS. The psychometric properties of most included PROMs were rated with insufficient evidence. The PROMs that received class A recommendation were the Poz Quality of Life, HIV Symptom Index or Symptoms Distress Module of the Adult AIDS Clinical Trial Group, and People Living with HIV Resilience Scale. In addition, because of a lack of evidence, recommendations regarding use could not be made for most of the remaining assessed PROMs (received class B recommendation).

**Conclusions:**

This systematic review recommends 3 PROMs to assess health outcomes in adults living with HIV and AIDS. However, all these PROMs have some shortcomings. In addition, most of the included PROMs do not have sufficient evidence for assessing their psychometric properties and require a more comprehensive validation of the psychometric properties in the future to provide more scientific evidence. Thus, our findings may provide a reference for the selection of high-quality HIV-specific PROMs by health care providers and researchers for clinical practice and research.

## Introduction

### Background

According to the statistics from the Joint United Nations Program on HIV/AIDS, 28.2 million individuals were accessing antiretroviral therapy (ART) as of mid-2021 [1]. Although effective treatment via ART has improved the life expectancy of people living with HIV and AIDS [2], this population still faces substantial challenges brought by HIV [3-6]. Therefore, Lazarus et al [7] proposed the *Fourth 90* target to ensure that 90% of people living with HIV and AIDS with viral suppression have a good health-related quality of life (HRQoL) after the World Health Organization proposed the *90-90-90* targets. They proposed that HRQoL in people living with HIV and AIDS should be considered as important as viral suppression [8]. For people living with HIV and AIDS, the focus should be shifted toward improving HIV-related care [9].

The management of people living with HIV and AIDS is multidimensional and complex. To overcome the obstacles to achieving the *Fourth 90* [10], patient-centered care that can meet the lifelong needs of people living with HIV and AIDS from diagnosis to death is the key requirement [9]. The collection and use of patient-reported outcome (PRO) data is one of the most effective approaches for ensuring that the care reflects the needs and priorities of people living with HIV and AIDS [9]. Compared with clinician-reported outcomes, PROs present a more comprehensive method for assessing the subjective perceptions of people living with HIV and AIDS of their own health that cannot be observed or are not easily observed directly and have been shown to accurately predict health outcomes among this population [11,12]. Furthermore, there is sufficient evidence that PROs can be used to improve the care quality and health outcomes in people living with HIV and AIDS, such as by improving patient-physician communication [13], clinical decision-making [14], and symptom recognition [15].

### Why Did This Systematic Review Only Include HIV-Specific PRO Measures?

Patient-reported outcome measures (PROMs) are the actual tool developed for collecting PRO data. There are 2 types of PROMs: generic (designed for use in any population and cover general aspects of outcome measures) and disease specific (designed for use in people with a condition and measure specific aspects of an outcome of importance). Many generic and HIV-specific PROMs have been validated in people living with HIV and AIDS. The advantage of a generic PROM is that it enables researchers to compare the health outcomes of people living with HIV and AIDS with those of other populations based on the same measurements [16]. However, unlike generic PROMs, HIV-specific PROMs do not have a significant ceiling and floor effect and do not overestimate health outcomes in people living with HIV and AIDS [17,18]. Furthermore, HIV-specific PROMs are more closely associated with HIV than are generic PROMs. In addition, they have the sensitivity for detecting and quantifying minor changes and specificity needed for HIV-specific domains, such as HIV-related stigma, comorbidities, and ART-related treatment [19]. Some related reviews have recommended a strategy to combine generic and HIV-specific PROMs to supplement HIV-specific health care outcomes that cannot be obtained with generic PROMs alone [20,21]. Clayson et al [20] suggested that the right combination of generic and HIV-specific PROMs can improve the comprehensiveness of assessment content, such that it includes not only the 3 core domains that generic PROMs focus on, that is, physical function, social or role function, and mental health or emotional well-being, but also the items or domains addressing issues relevant to HIV or AIDS and its treatment. Considering that many HIV-specific PROMs were developed before the widespread use of ART, they may not be able to detect the impact of current treatment on people living with HIV and AIDS and serve as an assessment tool for the long-term management of people living with HIV and AIDS [9]. In addition, many poorly designed PROMs lack a standardized development process. Therefore, it is necessary to summarize the existing HIV-specific PROMs and assess their psychometric properties.

### Previous Studies

With the rapid development of this field, many HIV-specific PROMs have been developed. After a preliminary literature search in MEDLINE using a comprehensive search strategy (Table S1 in Multimedia Appendix 1), we found some relevant reviews. Wen et al [19] recently conducted a systematic review on a similar topic; however, they only aimed at identifying and assessing the psychometric properties of HRQoL in people living with HIV and AIDS. Engler et al [22] identified 117 different HIV-specific PROMs in 2016; however, they did not quantitatively assess the psychometric properties of these PROMs. Cooper [16] reported an overview of the available reviews and summarized the PROMs with <40 items for measuring HRQoL in people living with HIV and AIDS in 2017. Earlier, several researchers conducted nonsystematic reviews of some PROMs in specific contexts [20,23,24]. Although many previous reviews have summarized the content of some existing HIV-specific PROMs, few have comprehensively reported the psychometric properties of these PROMs and given recommendations for the use of these PROMs.

As accurate and reliable PROMs are a precondition for obtaining robust results, PROMs with good psychometric properties are indispensable for research [25]. *Lancet HIV* also suggested in the special issue of “HIV outcomes beyond viral suppression” that the psychometric properties of the existing PROMs should be assessed in line with the existing guidelines, such as the Consensus-Based Standards for the Selection of Health Measurement Instruments (COSMIN) guidelines [9]. The COSMIN guidelines provide a consecutive procedure to help health care providers and researchers improve the selection of the most suitable PROMs in research and clinical practice [26]. Therefore, we conducted a systematic review to identify studies assessing the psychometric properties of HIV-specific PROMs validated in a population of adults living with HIV and AIDS and categorized these PROMs based on the type of outcome measure. We further assessed the methodological quality and level of evidence of these PROMs in association with their psychometric properties.

### Objective

The purpose of this systematic review was to summarize and categorize the available and validated HIV-specific PROMs for adults living with HIV and AIDS. This systematic review also aimed to use the COSMIN methodology to assess the psychometric properties of these PROMs and make an evidence-based and completely transparent recommendation for the use of these PROMs.

## Methods

### Overview

This systematic review was conducted and reported according to the COSMIN guidelines [27] and the PRISMA (Preferred Reporting Items for Systematic Reviews and Meta-Analyses) statement [28]. It included only a secondary data analysis of publicly available content not involving human participants. Therefore, ethics approval was not required for this review.

### Search Strategy

Three literature databases (MEDLINE, Embase, and PsycINFO) were searched on January 15, 2021. Two important web databases, PROQOLID and PROMIS, which contain a large number of PROMs and cover a wide range of populations and therapeutic areas, were also searched for PROMs. These 2 databases were developed by the Mapi Research Trust in France and the National Institutes of Health in the United States to facilitate the selection process of PROMs and are now used by many clinical investigators. The reference lists of relevant reviews in the preliminary literature search and the included studies were further examined for relevant publications. The search strategy used three COSMIN-guided search terms in reference to the search for constructs developed by Terwee et al [29]: (1) construct of interest, (2) condition of interest, and (3) psychometric properties (Table S2 in Multimedia Appendix 1). A comprehensive search strategy was developed under the guidance of a senior health research librarian.

### Study Selection

The eligibility criteria of the studies were as follows: (1) the study validated HIV-specific PROMs for adults living with HIV or AIDS and assessed at least one of the 9 psychometric properties defined by the COSMIN guidelines: content validity, structural validity, internal consistency, cross-cultural validity or measurement invariance, reliability, measurement error, criterion validity, hypotheses testing for construct validity, and responsiveness [30]; (2) the study was published in English in a peer-reviewed journal; and (3) the study applied self-administered PROMs for patients.

Studies were excluded if (1) they used the PROM mainly for outcome measures rather than for assessing the 9 psychometric properties; (2) they developed and used PROMs for screening or diagnostic purposes only; (3) they were not an original investigation, such as reviews, letters, and editorials; (4) they included generic PROMs or other disease-specific PROMs not related to or only partially related to HIV (such as the 36-Item Short-Form Health Survey Questionnaire); and (5) they provided indirect evidence of psychometric properties (such as studies using a PROM in a validation study of another instrument [30]).

The retrieved literature was imported into the EndNote software (version X9; Clarivate Plc), and duplications were automatically removed. A 2-stage screening process was used to select eligible studies. First, the titles and abstracts were screened based on the predetermined selection criteria (stage I). Subsequently, the full texts of articles deemed relevant or possibly relevant were obtained and further assessed for eligibility (stage II). Two independent researchers (ZW and YZ) determined study eligibility, and any disagreement was settled by consensus or discussion with a third researcher (BQ).

### Data Exclusion

For the eligible studies, data were independently extracted by the same 2 researchers (ZW and YZ) using a standardized form, and completeness and correctness were confirmed. Any discrepancy was resolved via a discussion with the third researcher (BQ). The extracted data included the characteristics of PROMs (name of the PROM[abbreviation], year of PROM development, targeted concept, recall period, number of items, each domain and the number of items in each domain, response options and score range, and original language), characteristics of the included studies (first author [year of publication], the total number of patients [N], age, gender, patient description, years diagnosis, severity of disease, recruitment context, country of research, and effective response rate of the questionnaire), and results of the included studies (COSMIN risk of bias information, evidence of the 9 psychometric properties, and COSMIN summary and rating).

### Data Analysis

According to the suggestions mentioned in the COSMIN guidelines, each PROM was assessed via a 4-step process [27]. First, the methodological quality for every psychometric property in each study was assessed using the COSMIN risk of bias checklist based on a four-point response, “very good,” “adequate,” “doubtful,” or “inadequate,” and an overall rating of the psychometric property was determined based on the item with the worst rating [30]. Second, the results for every psychometric property in each study were rated based on the updated criteria for good psychometric properties [27], and each result was graded as positive (+), negative (–), or indeterminate (?). Third, the overall results for each psychometric property of a PROM were rated as sufficient (+), insufficient (–), inconsistent (±), or indeterminate (?), and the level of evidence for each psychometric property of a PROM was rated as “high,” “moderate,” “low,” or “very low” by following the Grading of Recommendations, Assessment, Development, and Evaluation approach, which considered the initial level of evidence to be high, with subsequent downgrading based on the score for 4 criteria: risk of bias, inconsistencies, imprecision, and indirectness. Finally, a table summarizing the findings was constructed and used to make recommendations for the selection of the most suitable PROMs.

All assessments were conducted independently by 3 researchers (ZW, HK, and XD), and any disagreement was settled via consensus or discussion with a fourth researcher (YZ). The Cohen κ coefficient was calculated using the SPSS software (version 24.0; IBM) to evaluate the interrater agreement for title and abstract screening, study selection, and data extraction.

## Results

### Search Results

**Figure 1:**
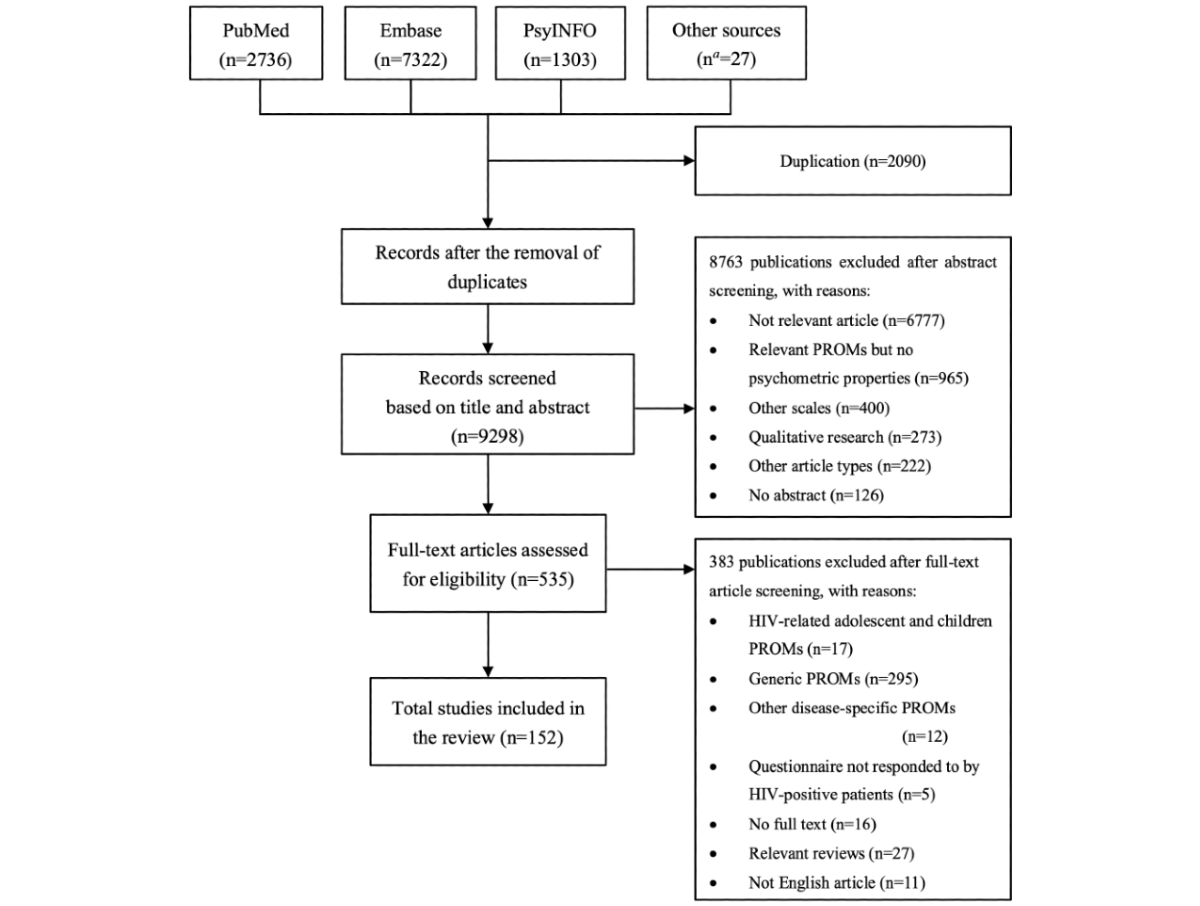
The PRISMA (Preferred Reporting Items for Systematic Reviews and Meta-Analyses) flow diagram. PROM: patient-reported outcome measure. ^a^These studies were identified through further research of the reference lists of relevant reviews in the preliminary literature search and the included studies.

A total of 11,361 articles were identified in the literature search, and another 27 articles were identified through reference and citation searches. Of these, 2090 were excluded because of duplication. After screening the titles and abstracts, 535 articles were found to be potentially relevant, and their full text was reviewed for further assessment. Of these, 152 articles were finally included [31-182]. The PRISMA flow diagram and the reasons for exclusion are presented in Figure 1. The average Cohen κ coefficients for the title and abstract screening, study selection, and data extraction were 0.85, 0.82, and 0.89, respectively, indicating that the 2 researchers reached a “substantial agreement” as defined by Landis and Koch [183] in 1991.

### Characteristics of the Included PROMs

Table 1 lists the characteristics of the included PROMs, with details of the subscales provided in Table S3 in Multimedia Appendix 1. A total of 88 PROMs were reported in the 152 included studies, and these PROMs can be divided into 8 categories (improved based on the initial taxonomy developed by Engler et al [22]): HRQoL (24/88, 27% of PROMs) [31-102], symptoms (10/88, 11% of PROMs) [103-120], stigma (15/88, 17% of PROMs) [121-142], psychological (8/88, 9% of PROMs) [143-151], body and facial appearance (5/88, 6% of PROMs) [152-156], treatment (17/88, 19% of PROMs) [153-173], social support (3/88, 3% of PROMs) [174-176], and self-management and self-care (6/88, 7% of PROMs) [177-182]. All the included PROMs were tools self-administered by people living with HIV and AIDS either in a clinical or research context. Of these 88 PROMs, 22 (25%) PROMs were developed before 2000, 31 (35%) between 2000 and 2009, and 35 (40%) after 2010. The recall period for PROMs ranged from “past 7 days” to “last 12 months.” The number of items varied between 4 and 165. The original language for most PROMs was English, and the response option format for most PROMs was the 5-point Likert scale.

**Table 1:** Characteristics of the included patient-reported outcome measures (PROMs)^a^.

PROM; year of development		Targeted concept	Recall period	Total no. of items	Response options	Score range	Original language
**HRQoL^b^**							
	MOS-HIV^c^ [31-51]; 1996	HRQoL	Past 4 weeks	35	Multiple response options	Raw scores for each scale were transformed to a scale of 0 to 100	English
	MOS-HIV-17 [53]; 2000	HRQoL	Past 4 weeks	17	Multiple response options	Raw scores for each scale were transformed to a scale of 0 to 100	English
	MOS-HIV-29 [52]; 2012	HRQoL	Past 30 days	29	Multiple response options	Raw scores for each scale were transformed to a scale of 0 to100	Luganda
	HIV Overview of Problems Evaluation System [54,55]; 1992	HRQoL	—^d^	165	5-point Likert scale (0-4)	Summary of scales: physical scale, medical interaction, psychosocial scale, sexual scale, and significant others or partners	English
	HIV-Related Quality of Life Questions [56]; 1993	HRQoL	Past month	34	Multiple response options	—	English
	AIDS Health Assessment Questionnaire [57]; 1997	HRQoL	Different recall periods per dimensions	116	Multiple response options	Raw scores were transformed to a scale of 0 to 100	English
	HIV-PARSE^e^ [58]; 1994	HRQoL	Different recall periods per dimensions	30	Multiple response options	Perceived Health Index (25 items)	English
	HIV-PARSE-Brief [59]; 1995	HRQoL	Different recall periods per dimensions	21	Multiple response options	Perceived Health Index (13 items)	English
	HRQoL [60]; 1995	HRQoL	Past 4 weeks	64	Multiple response options	A physical health dimension and a Mental health dimension	English
	Functional Assessment of HIV Infection [61-65]; 1996	HRQoL	Past 7 days	44	5-point Likert scale (0-4)	Sum of all item scores (0-176)	English
	General Health Self-Assessment [66]; 1997	HRQoL	Past 4 weeks	49	Multiple response options	The subscales are scored as summated and transformed on a scale of 0 to 100	English
	HIV Quality of Life 31-item scale [67]; 1997	HRQoL	—	31	Dichotomous: yes or no	Simple summation of dichotomous response options	French
	HAT-QoL^f^-42 [68,69]; 1997	HRQoL	Past 4 weeks	42	5-point Likert scale (1-5)	All subscales are coded to range from 0 to 100	English
	HAT-QoL-30 [35]; 1999	HRQoL	Past 4 weeks	30	5-point Likert scale (1-5)	All subscales are coded to range from 0 to 100	English
	HAT-QoL-34 [42,70,71]; 2008	HRQoL	Past 4 weeks	34	5-point Likert scale (1-5)	All subscales are coded to range from 0 to 100	English
	MQoL^g^ for patients with HIV or AIDS [34,72-75]; 1997	HRQoL	—	40	7-point Likert scale (1-7)	Each subscale ranged from 4 to 28; mental health score + (2 × physical functioning score) = overall index for MQoL (12-84)	English
	Living with HIV Scale [76]; 1998	HRQoL	—	32	5-point Likert scale (0-4)	Sum of all item scores: 0-128; subscale scores range: 0-24	English
	WHOQOL-HIV^h^ [77-83]; 2004	HRQoL	The last 2 weeks	120 (30 facets)	5-point Likert scale (1-5)	Facet scores range: 4-20	English
	WHOQOL-HIV-BREF^i^ [84-95]; 2012	HRQoL	The last 2 weeks	31	5-point Likert scale (1-5)	Facet scores range: 4-20	English
	Instituto Superiore di Sanità Quality of Life [96]; 2006	HRQoL	Past 4 weeks	62	5-point Likert scales (1-5)	All subscales are coded to range from 0 to 100	Italian
	Symptom Quality of Life Adherence [97]; 2009	HRQoL	Past 4 weeks	26	HRQoL: 5-point Likert scales (1-5), symptoms: yes or no, and adherence: 10 cm VAS^j^	HRQoL: standardized sum (0-100), symptoms: summed, and adherence: score 0-10 VAS	French
	PROQOL-HIV^k^-43 [98-100]; 2012	HRQoL	Past 2 weeks	43	5-point Likert scale (0-4)	Sum of the 8 subscales and coded as a total score range from 0 to 100	English
	PROQOL-HIV-38 [101]; 2016	HRQoL	Past 2 weeks	38	5-point Likert scale (0-4)	Four subscale scores are summed of item responses, coded to range from 0 to 100	French
	Poz Quality of Life [102]; 2018	HRQoL	—	13	5-point Likert scale (1-5)	Items were averaged to create the total score and scores for each subscale	English
**Symptoms**							
	Riverside Symptom Checklist [103]; 1993	HIV-related symptoms	Past 3 months	28	5-point Likert scale (0-4)	The subscales are scored as summated and transformed on a scale from 0 to 100	English
	HIV Symptom Index [104]; 1994	HIV-related symptoms	Past 2 weeks	12	4-point Likert scales (0-3)	Scores range: 0-24	English
	HIV Assessment Tool [105]; 1994	HIV-related symptoms	—	34 in each exploratory factor analysis	100-mm linear scale	Items were averaged to create the total score (0-100)	English
	SSC-HIV^l^ [106,107]; 1999	HIV-related symptoms	—	26	4-point Likert scales (0-3)	The items within a factor are summed for a subscale score	English
	SSC-HIV-rev [108]; 2001	HIV-related symptoms	—	72	4-point Likert scales (0-3)	The items within a factor are summed for a subscale score	English
	HIV Cost and Services Utilization Study Symptom Measure [109]; 2000	HIV-related symptoms	Preceding 6 months	13 for male and 14 for female respondents	5-point Likert scale (1-5)	The subscales are scored as summated and transformed on a scale of 0 to 100	English
	HIV Symptom Index or Symptoms Distress Module of the ACTG^m^ [110-112]; 2001	HIV-related symptoms	Past 4 weeks	20	5-point Likert scale (0-4)	Score range: 0-80	English
	HIV-Related Fatigue Scale [113-115]; 2002	HIV-related fatigue	Past week	56	Multiple response options	All subscales are coded to range from 1 to 10	English
	HIV Disability Questionnaire [116-119]; 2013	HIV-related disability	Past week	69	Disability presence scores: yes or no; disability severity scores: 5-point Likert scale (0-4); episodic scores: yes or no; 7-point Likert scale (0-6) [116]	Each method of calculating scores was to sum the scores and transform them into scores out of 100	English
	Istituto Superiore di Sanità-HIV symptoms scale [120]; 2016	HIV-related symptoms	Past 4 weeks	22	5-point Likert scale (no score for each option)	—	Italian
**Stigma**							
	HSS^n^-40 [121,122]; 2001	HIV-related stigma	—	40	4-point Likert scale (1-4)	Score range: 40-160	English
	HSS-32 [123,124]; 2007	HIV-related stigma	—	32	4-point Likert scale (1-4)	Score range: 32-128	English
	HSS-12 [125-127]; 2010	HIV-related stigma	—	12	4-point Likert scale (1-4)	Score range: 12-48	Swedish
	HSS-39 [128]; 2014	HIV-related stigma	—	39	4-point Likert scale (1-4)	Score range: 39-156	Swedish
	HSS-30 [129]; 2015	HIV-related stigma	—	30	4-point Likert scale (1-4)	Score range: 30-120	Spanish
	HSS-10 [130]; 2020	HIV-related stigma	—	10	5-point Likert scale (1-5)	Score range: 10-50	Japanese
	HIV or AIDS stigma instrument–People living with AIDS [131,132]; 2007	HIV-related stigma	Two recall periods: past 3 months and ever since HIV diagnosis	33	4-point Likert scale (0-3)	Score range: 0-3	English
	Internalized HIV Stigma Measure [133]; 2008	Internalized HIV-related stigma	—	28	Transformed linearly to a range of 0 to 100	The subscales are scored as summated and transformed on a scale of 0 to 100	English
	Internalized AIDS-Related Stigma Scale [134-136]; 2009	Internalized HIV-related stigma	—	6	Binary response: 1=agree and 0=disagree	Total scores range of endorsed stigma items: 0-6	English
	Internalized Stigma in Those With HIV or AIDS [137]; 2011	Internalized HIV-related stigma	Ever since HIV diagnosis	10	5-point Likert scale (1-5)	Score range: 10-50	English
	HIV- and Abuse-Related Shame Inventory [138]; 2012	HIV- and abuse-related shame	Past month	31	5-point Likert scale (0-4)	Score range: 0-124	English
	Self, Experienced, and Perceived HIV or AIDS Stigma Scales [139]; 2012	HIV-related stigma	—	22	4-point Likert scale (1-4)	Score range: 22-88	English
	HIV Stigma Mechanisms [140]; 2013	HIV stigma mechanisms	—	24	5-point Likert scales (1-5)	Items were averaged to create composite scores	English
	HIV or AIDS Stigma Assessment for Latino Gay Men, Bisexual Men, and Transgender Women Living With HIV [141]; 2013	HIV-related stigma	—	36	4-point Likert scale (1-4)	Score range: 36-144	English
	Van Rie HIV or AIDS-Related Stigma Scale-Revised for use in the United States [142]; 2015	HIV-related stigma	—	15	4-point Likert scale (0-3)	Items were averaged to create composite scores and subscales scores	English
**Psychological**							
	The Mental Adjustment to HIV scale [143]; 1994	Mental adjustment	—	40	4-point Likert scale (1-4)	The subscales are scored as summated	English
	HIV or AIDS Stress Scale [144]; 2002	Stress and coping	Past month	23	5-point Likert scale (0-4)	Score range: 0-92	English
	Perceived Stress Scale Among People Living With HIV or AIDS [145]; 2008	HIV-related stress	Past month	35	5-point Likert scale (1-5)	Score range: 35-175	Simplified Chinese
	Screenphiv [146,147]; 2012	Psychological issues related to HIV	—	63	VAS (0-100 mm)	Items were averaged to create composite scores	Spanish
	Impact on Self-Concept Scale [148]; 2013	Impact of HIV on self-concept	—	10	6-point Likert scale (1-6)	Items were averaged to create composite scores	English
	Impact of HIV [149]; 2015	Challenges of HIV survivorship	—	38	5-point Likert scale (1-5)	Score range: 38-190	English
	HIV Meaningfulness Scale [150]; 2015	HIV meaningfulness	—	4	7-point Likert scale	Score range: 1-28	English
	People Living with HIV Resilience Scale [151]; 2019	Resilience	Past 12 months	10	Positively affected: “+1,” not affected: “0,” and negatively affected: “–1”	Score range: (–10 to 10)	English
**Body and facial appearance**							
	Body Image in Patients With HIV or AIDS [152]; 2005	Perceived body image	—	12	5-point VAS	Score range: 12-60	English
	Owen Clinic Lipodystrophy Scale [153]; 2006	Body change	—	12	Dichotomous: (yes or no)	—	English
	ACTG-ABCD^o^ [154]; 2006	Body change and distress	Part 3: past 4 weeks	27	Part 1: dichotomous: (yes or no); part 2 and part 3: 5-point Likert scale (1-5)	Sum of all item scores in part 3 (20 items)	English
	ACTG-ABCD Short form [155]; 2014	Body change and distress	Past 4 weeks	18	5-point Likert scale (1-5)	Sum of all item scores	English
	Facial Appearance Inventory [156]; 2016	Appearance	Past 4 weeks	10	7-point Likert scale (1-7)	Score range: 24-168; final score is linearly transformed to 0-100	English
**Treatment**							
	Medication Attribution Scale [157]; 1998	Attributions about ART^p^ (its limitations on functioning, etc)	—	10	11-point Likert scale (0-10)	Sum of all item scores	English
	HIVTSQ^q^ [158]; 2001	Satisfaction with ART	Past 4 weeks	9	7-point Likert scale (0-6)	Total treatment satisfaction is the sum of the 9 item scores	English
	HIV Treatment Satisfaction Questionnaire status version [159]; 2006	Satisfaction with ART	Past few weeks	10	7-point Likert scale (0-6)	Total treatment satisfaction is the sum of the 10 item scores	English
	Treatment-Related Empowerment Scale [160]; 2001	Empowerment (involvement in treatment decision-making)	—	10	5-point Likert scale (1-5)	Sum of all item scores	English
	Subcutaneous Injection Survey [161]; 2002	Satisfaction with ART–subcutaneous injection	—	15	5-point Likert scale (1-5)	Score range: 20-100	English
	Quality of care through the patient’s eyes [162]; 2003	Quality of care	—	27	Importance and performance were measured using a 4-point Likert scale (1-4)	[Qij = Iij × Pij]^r^	English
	Attitudes Toward HIV Health Care Provider scale [163]; 2004	Attitudes toward health care providers	—	19	6-point Likert scale (1-6)	Sum of all item scores	English
	Antiretroviral General Adherence Scale [164]; 2006	Ease and ability to adhere to ART	Past 30 days	5	6-point Likert scale (1-6)	Sum of all item scores or a proportion (by dividing this score by the total possible score)	English
	Health Care Relationship Trust Scale [165]; 2006	Trust toward health care providers	—	15	5-point Likert scale (0-4)	Sum of item scores and the mean of item scores	English
	HIV Medication Readiness Scale [166]; 2007	Readiness to adhere to ART	—	10	5-point Likert scale (0-4)	Score range: 0-40	English and French
	SECope [167]; 2007	Coping with the side effects of ART	—	20	5-point Likert scale (0-4)	Score range: 0-80	English
	HIV Treatment Optimism Scale [168]; 2009	Optimism about ART	—	19	7-point Likert scale (1-7)	Score range: 19-133	English
	HIV Medication Taking Self-Efficacy Scale [169]; 2010	Self-efficacy to adhere to ART	—	26	11-point Likert scale (0-10)	Sum of all item scores	English
	Brief Estimate of Health Knowledge and Action-HIV version [170]; 2010	ART-related health literacy	—	8	Part I: 4-point Likert scale (0-3); Part II: 6-point Likert scale (0-5)	Sum of all item scores	English
	HIV Treatment Readiness Measure [171]; 2011	Factors affecting the readiness for ART	Alcohol and drug use subscale in the past 3 months	38	5-point Likert scale (1-5)	Sum of all item scores and the mean of all item scores	English
	HIV Treatment Regimen Fatigue Scale [172]; 2015	Regimen fatigue	—	22	–3 to 3 (excluding 0)	Sum of all item scores	English
	HIV Engagement in and Continuity of Care Scale [173]; 2017	Engagement in care	—	26	5-point Likert scale	—	English
**Social support**							
	Social Support Inventory [174]; 1999	Received social support	—	14/17	Satisfaction: 5-point Likert scale (1-5); want: yes or no; have: yes, no, or not applicable	Nine subscales: 0-5	English
	Unsupportive Social Interactions Inventory-HIV version [175]; 1999	Unsupportive social interactions	—	24	4-point Likert scale (0-4)	An overall score, the Unsupportive Social Interactions Inventory-18, is based on 3 of its subscales	English
	Perceived Social Support for HIV [176]; 2014	Perceived social support	—	12	5-point Likert scale (1-5)	Sum of all item scores; Score range: 12-60	Spanish
**Self-management and self-care**							
	HIV Treatment Adherence Self-Efficacy Scale [177]; 2007	Self-efficacy to adhere to HIV care	Past 1 month	12	11-point Likert scale (0-10)	Item scores were averaged for each respondent	English
	Perceived HIV Self-Management Scale [178]; 2011	Self-efficacy for HIV self-management	—	8	6-point Likert scale (1-6)	Sum of all item scores	English
	HIV Self-Management Scale (Women) [179]; 2012	HIV Self-Management Scale (Women)	—	20	4-point Likert scale (0-3)	Subscale score range: 0-3	English
	HIV Intention Measure [180]; 2012	Intention to adhere to HIV care	—	14	6-point Likert scale (1-6)	—	English
	HIV Exercise Stereotypes Scale [181]; 2016	Stereotypes related to exercise in people living with HIV	—	14	6-point Likert scale (1-6)	Three subscale scores are computed as the mean of item responses	French
	HIV Symptom Management Self-Efficacy for Women Scale [182]; 2011	Self-efficacy for HIV symptom management	—	9	11-point Likert scale (0-10)	The final score is calculated as the mean of the 9 item scores	English

^a^Each version of a PROM is considered a separate PROM.

^b^HRQoL: health-related quality of life.

^c^MOS-HIV: Medical Outcomes Study-HIV Health Survey.

^d^—: not reported.

^e^HIV-PARSE: HIV Patient–Reported Status and Experience.

^f^HAT-QoL: HIV or AIDS-Targeted Quality of Life Instrument.

^g^MQoL: Multidimensional Quality of Life.

^h^WHOQOL-HIV: World Health Organization Quality of Life-HIV.

^i^WHOQOL-HIV-BREF: World Health Organization Quality of Life-HIV-Bref instrument.

^j^VAS: visual analog scale.

^k^PROQOL-HIV: Patient-Reported Outcome Quality of Life-HIV Questionnaire.

^l^SSC-HIV: Sign and Symptom Checklist for HIV.

^m^ACTG: Adult AIDS Clinical Trial Group.

^n^HSS: HIV Stigma Scale.

^o^ACTG-ABCD: Adult AIDS Clinical Trial Group’s Assessment of Body Change and Distress.

^p^ART: antiretroviral therapy.

^q^HIVTSQ: HIV Treatment Satisfaction Questionnaire.

^r^The quality improvement score (Q) on a health service (j) by an individual patient (i) is equal to the importance score (I) multiplied by the (perceived) performance score (P).

### Characteristics of the Included Records

As 3 studies [34,35,42] included the assessment of 2 PROMs, 155 records were included. Table S4 in Multimedia Appendix 1 shows the characteristics of the 155 included records. Of these 155 records, 31 (20%) records were reported before 2000, 46 (29.7%) records were reported between 2000 and 2009, and 78 (50.3%) records were reported after 2010. The total sample size of these records was 79,213 (range 20-5521). There were more men than women in 83.2% (129/155) of the records, and 1.3% (2/155) of records did not indicate gender data. Most records gave the mean (SD) or median (IQR) age data for samples (range 16-84 years), and 8.4% (13/155) of records indicated no age data. There were 70.3% (109/155) records from high-income countries (64/155, 41.3% records from the United States), 20.6% (32/155) records from low- and medium-income countries (9/155, 5.8% records from China), and 9% (14/155) of records from multiple countries. Table S4 in Multimedia Appendix 1 also summarizes the years since diagnosis, the severity of the disease, recruitment context, and effective response rate.

### Methodological Quality Assessment

The methodological quality for each psychometric property of every record is summarized in Table S5 in Multimedia Appendix 1 based on the COSMIN risk of bias checklist. As there is no generally accepted “golden standard” for assessing health outcomes in adults living with HIV and AIDS, the criterion validity of all studies was not considered. Most records assessed internal consistency (146/155, 94.2% of records) and structural validity (96/155, 61.9% of records), and most of them were rated as “very good” or “adequate.” Although 79.4% (123/155) of records assessed the hypotheses testing for construct validity, most were rated as “doubtful” or “inadequate.” As for the remaining psychometric properties, only a few records assessed them, and most of them were rated as “doubtful” or “inadequate.”

### Overall Results and the Level of Evidence

Table S6 in Multimedia Appendix 1 shows the results of each psychometric property of each record. The overall results and the level of evidence are presented in Table S7 in Multimedia Appendix 1. There are only few studies on PROMs, except for some well-known PROMs; accordingly, there is little evidence for psychometric properties.

Of the 88 PROMs, PROM development was assessed in 18% (16/88) PROMs, and original content validity was assessed in 3% (3/88) PROMs. However, no PROM exhibited “sufficient” high-quality evidence for content validity. Subsequently, we found that 16% (14/88) of the PROMs had “sufficient” high-quality evidence of structural validity; however, most others had “indeterminate” moderate-quality evidence. The internal consistency for a PROM can be assessed only if it has at least low-quality evidence for “sufficient” structural validity; otherwise, the internal consistency will be considered “indeterminate” [30]. Therefore, although 83% (73/88) of PROMs presented high-quality evidence for internal consistency, only 16% (14/88) demonstrated “sufficient” results. Evidence supporting hypotheses testing for construct validity was available for 81% (71/88) of the PROMs. Furthermore, reliability was assessed in 30% (26/88) PROMs, but no PROM presented “sufficient” high-quality evidence. The responsiveness of 8% (7/88) of PROMs was evaluated as “sufficient,” but only 2% (2/88) PROMs (Functional Assessment of HIV Infection [61-66] and HIV Medication Readiness Scale [166]) showed high-quality evidence. Cross-cultural validity or measurement invariance was assessed in only 6% (5/88) of PROMs with low or very low quality [82,111,122,124,127]. Finally, only 1% (1/88) of PROMs assessed measurement error with “indeterminate” low-quality evidence [118].

### Recommendations

The following recommendations are presented according to the COSMIN guidelines (Table 2):

- Class A: The PROMs with evidence for “sufficient” content validity (any level) and at least low-quality evidence for “sufficient” internal consistency included the following: Poz Quality of Life (PozQoL) [102], HIV Symptom Index or Symptoms Distress Module of the Adult AIDS Clinical Trial Group (HIV-SI or SDM) [110-112], and People Living with HIV Resilience Scale (PLHIV-RS) [151]. These may be recommended for use, and the results obtained may be credible.
- Class B: The remaining PROMs have the potential to be recommended for use; however, further research is required to assess their quality (PROMs not included in class A or C).
- Class C: The PROMs with high-quality evidence for an “insufficient” psychometric property included the following: Multidimensional Quality of Life for patients With HIV and AIDS [72-75], Patient-Reported Outcome Quality of Life-HIV Questionnaire-38 [101], HIV-Related Fatigue Scale [113-115], HIV Stigma Scale-10 [130], HIV or AIDS Stress Scale [144], Screenphiv [146,147], SECope [167], and HIV Exercise Stereotypes Scale [181]. They may not be recommended for use.

Although 3 PROMs have been recommended, they all have some shortcomings, reducing the strength of the recommendation for their routine use. Furthermore, although PozQoL [102] and PLHIV-RS [151] achieved class A, they were developed and assessed based on a single validation study. In addition, some items in HIV-SI or SDM have significant differential item functioning between different cultural groups [111], indicating low-quality evidence for “insufficient” cross-cultural validity.

**Table 2:** Summary of findings^a^.

PROM^b^	Content validity		Structural validity			Internal consistency^c^			CCV or MI^d^			Reliability			Measurement error			HTCV^e,f^			Responsiveness			Class^g^
	Results	LoE^h^	Results	LoE	Results		LoE	Results		LoE	Results		LoE	Results		LoE	Results		LoE	Results		LoE		
MOS-HIV^i^ [31-51]			+	M	±		M				±		M				+		H				B	
MOS-HIV-17 [53]					?		H										–		L				B	
MOS-HIV-29 [52]			?	M	?		M										+		L	+		L	B	
HOPES^j^ [54,55]					?		H										+		VL	+		VL	B	
HIV-QoL^k^ [56]					?		H																B	
AIDS-HAQ^l^ [57]					?		H										+		H	+		L	B	
HIV-PARSE^m^ [58]					?		H																B	
HIV-PARSE-Brief [59]					?		H																B	
HRQOL^n^ [60]			–	VL	?		H										+		L				B	
FAHI^o^ [61-65]	±	M	–	M	?		H										+		H	+		H	B	
GHSA^p^ [66]			?	M	?		H										+		L				B	
HIV-QL31^q^ [67]	?	VL	–	M	?		H										–		L				B	
HAT-QoL^r^-42 [68,69]					?		H										–		M				B	
HAT-QoL-30 [35]					?		H										+		L				B	
HAT-QoL-34 [42,70,71]			?	M	?		H				–		M				+		H				B	
MQoL-HIV^s^ [34,72-75]	?	M	?	M	?		H				–		H				+		H	+		M	C	
LWHIVS^t^ [76]	±	VL	?	M	?		H										–		L				B	
WHOQOL-HIV^u^ [77-83]			+	M	?		L	?		VL	+		M				+		H				B	
WHOQOL-HIV-BREF [84-95]			±	L	?		H				?		M				+		L				B	
ISSQoL^v^ [96]	+	L			?		H										+		L				B	
HIV-SQUAD^w^ [97]			?	M	?		H										+		L				B	
PROQOL^x^-HIV-43 [98-100]	+	VL	?	M	?		H				+		L				+		L				B	
PROQOL-HIV-38 [101]			–	H	?		H										+		VL				C	
PozQol^y^ [102]	+	L	+	H	+		H				+		M				+		M				A	
RSC^z^ [103]					?		H										+		H				B	
HSI^aa^ [104]					?		H				+		L				–		L	+		VL	B	
HAT^ab^ [105]			?	VL	?		VL				+		L										B	
SSC-HIV^ac^ [106,107]			+	M	+		H																B	
SSC-HIV-rev [108]			?	M	?		H										–		L				B	
HCSUS-SM^ad^ [109]					?		H																B	
HIV-SI or SDM^ae^ [110-112]	+	L	+	H	+		H	–		L							+		L				A	
HRFS^af^ [113-115]	?	VL			?		H				–		H				+		M				C	
HDQ^ag^ [116-119]			+	M	+		H				+		M	?		L	±		M				B	
ISS-HIV-SS^ah^ [120]			?	M	?		H										+		H				B	
HSS^a^^i^-40 [121,122]			?	M	?		H	–		L	+		M				+		H				B	
HSS-32 [123,124]			±	M	?		H	–		VL							+		L				B	
HSS-12 [125-127]			+	H	+		H	–		L							–		M				B	
HSS-39 [128]			?	VL	?		H										+		VL				B	
HSS-30 [129]	?	M	+	H	+		H										+		H				B	
HSS-10 [130]			+	H	–		H										+		L				C	
HASI^aj^-P [131,132]			?	H	?		H										+		H				B	
IHSM^ak^ [133]			?	M	?		H										–		L				B	
IA-RSS^al^ [134-136]			+	H	+		H				–		M				±		M				B	
ISAT^am^ [137]			?	M	?		H										+		H				B	
HARSI^an^ [138]			?	M	?		H				–		VL										B	
SEP-HASS^ao^ [139]					?		H										+		VL				B	
HIV-SM^ap^ [140]					?		M										–		VL				B	
HA-SAL-GBT^aq^ [141]			?	VL	?		H										–		L				B	
VR-HARSSR^ar^ [142]			?	L	?		M										+		M				B	
MAH^as^ [143]			?	VL	?		H																B	
SS-HIV^at^ [144]			–	H	?		H				+		M				+		L				C	
PSSHIV^au^ [145]	±	VL	?	M	?		H				+		L				+		L				B	
Screenphiv [146,147]	?	L	+	H	–		H										+		H				C	
ISCS^av^ [148]			?	M	?		H										+		L				B	
IHIV^aw^ [149]			+	H	+		H																B	
HIVMS^ax^ [150]			?	M	?		H				+		L				+		VL				B	
PLHIV-RS^ay^ [151]	+	L	+	H	+		H										+		L				A	
BIS^az^ [152]			?	M	?		L				+		VL										B	
OCLS^ba^ [153]			?	M	?		VL																B	
ACTG-ABCD^bb^ [154]					?		H										–		L				B	
ACTG-ABCD-SF^bc^ [155]			?	M	?		H										+		L				B	
FAI^bd^ [156]	?	L			?		VL										+		VL				B	
MAS^be^ [157]					?		M										–		M				B	
HIVTSQ^bf^ [158]			?	M	?		H										–		L				B	
HIVTSQ status version [159]			+	H	+		H										–		L				B	
TES^bg^ [160]					?		L										–		VL				B	
SIS^bh^ [161]			?	M	?		H										–		L				B	
QUOTE-HIV^b^^i^ [162]					?		L																B	
AHHCP^bj^ [163]			?	M	?		H										+		H				B	
AGAS^bk^ [164]			?	M	?		H										+		L				B	
HCR^bl^ [165]	±	VL	?	L	?		M				–		VL				–		VL				B	
HMRS^bm^ [166]			?	M	?		H				+		VL				+		L	+		H	B	
SECope [167]	±	VL	+	H	–		H				–		M				–		L				C	
HTOS^bn^ [168]			?	M	?		H																B	
HIV-MT-SES^bo^ [169]			+	H	+		H				–		L				+		L				B	
BEHKA-HIV^bp^ [170]			?	M	?		H																B	
HTRM^bq^ [171]			?	M	?		H				–		M										B	
HTRFS^br^ [172]			?	L	?		M										+		VL				B	
HECCS^bs^ [173]			+	M	+		H										+		H				B	
SSI^bt^ [174]	±	VL	?	M	?		H										+		VL				B	
USII-HIV^bu^ [175]			?	M	?		H										–		L				B	
PSS-HIV^bv^ [176]					?		L										+		VL				B	
HIV-ASES^bw^ [177]			+	H	+		H				?		L				+		L				B	
PHIVSMS^bx^ [178]					?		H										+		L				B	
HIV-SMS-W^b^^y^ [179]	+	VL	+	H	+		H				?		VL										B	
HIV-IM^bz^ [180]	±	L	?	L	?		H										+		L				B	
HIVESS^ca^ [181]			–	H	?		H										+		VL				C	
HSM-SEWS^cb^ [182]			?	L	?		M				?		VL										B	

^a^As there is no generally accepted “golden standard” for assessing health outcomes in adults living with HIV and AIDS, the criterion validity of all studies was not considered. Overall results of PROMs are rated as +: sufficient; ?: indeterminate; ±: inconsistent; and –: insufficient. LoE is rated as H: high, M: moderate, L: low; VL: very low. Blank cells indicate that the data are not available.

^b^PROM: patient-reported outcome measure.

^c^Internal consistency can be rated as “sufficient” if there is at least low evidence for “sufficient” structural validity, and Cronbach α values≥.70 for each unidimensional scale or subscale; the evidence for “sufficient” structural validity may come from different studies, and the “at least low evidence” was defined by grading the evidence according to the Grading of Recommendations, Assessment, Development, and Evaluation approach.

^d^CCV or MI: cross-cultural validity or measurement invariance.

^e^HTCV: hypotheses testing for construct validity.

^f^The results of all included records should be taken together, and it should then be decided if 75% of the results are in accordance with the hypotheses. Only assessed measurement properties are shown.

^g^Class A represents evidence for sufficient content validity (any level) and at least low-quality evidence for sufficient internal consistency (PROMs can be recommended for use); class B, PROMs categorized not in class A or C; and class C, high-quality evidence for an insufficient measurement property; PROMs with class B recommendation require further evaluation to assess their quality before recommendation for use; PROMs with class C recommendation are not recommended for use.

^h^LoE: level of evidence (using the Grading of Recommendations, Assessment, Development, and Evaluations assessment tool).

^i^MOS-HIV: Medical Outcomes Study-HIV Health Survey.

^j^HOPES: HIV Overview of Problems Evaluation System.

^k^HIV-QoL: HIV-Related Quality of Life Questions.

^l^AIDS-HAQ: AIDS Health Assessment Questionnaire.

^m^HIV-PARSE: HIV Patient–Reported Status and Experience.

^n^HRQoL: health-related quality of life.

^o^FAHI: Functional Assessment of HIV Infection.

^p^GHSA: General Health Self-Assessment.

^q^HIV-QL31: HIV Quality of Life 31-item scale.

^r^HAT-QoL: HIV or AIDS-Targeted QoL Instrument.

^s^MQoL-HIV: Multidimensional QoL for patients with HIV or AIDS.

^t^LWHIVS: Living with HIV Scale.

^u^WHOQOL-HIV: World Health Organization Quality of Life-HIV.

^v^ISSQoL: Instituto Superiore di Sanità Quality of Life.

^w^HIV-SQUAD: Symptom Quality of Life Adherence.

^x^PROQOL-HIV: Patient-Reported Outcome Quality of Life-HIV Questionnaire.

^y^PozQol: Poz Quality of Life.

^z^RSC: Riverside Symptom Checklist.

^aa^HSI: HIV Symptom Index.

^ab^HAT: HIV Assessment Tool.

^ac^SSC-HIV: Sign and Symptom Checklist for HIV.

^ad^HCSUS-SM: HIV Cost and Services Utilization Study Symptom Measure.

^ae^HIV-SI or SDM: HIV Symptom Index or Symptoms Distress Module of the Adult AIDS Clinical Trial Group.

^af^HRFS: HIV-Related Fatigue Scale.

^ag^HDQ: HIV Disability Questionnaire.

^ah^ISS-HIV-SS: Istituto Superiore di Sanità-HIV symptoms scale.

^ai^HSS-40: HIV Stigma Scale.

^aj^HASI-P: HIV or AIDS Stigma Instrument-PLWA.

^ak^IHSM: Internalized HIV Stigma Measure.

^al^IA-RSS: Internalized AIDS-Related Stigma Scale.

^am^ISAT: Internalized Stigma in Those With HIV or AIDS.

^an^HARSI: HIV- and Abuse-Related Shame Inventory.

^ao^SEP-HASS: Self, Experienced, and Perceived HIV or AIDS Stigma Scales.

^ap^HIV-SM: HIV stigma mechanisms.

^aq^HA-SAL-GBT: HIV or AIDS Stigma Assessment for Latino Gay Men, Bisexual Men and Transgender Women Living With HIV.

^ar^VR-HARSSR: Van Rie HIV or AIDS-Related Stigma Scale-Revised for use in the United States.

^as^MAH: Mental Adjustment to HIV scale.

^at^SS-HIV: HIV or AIDS Stress Scale.

^au^PSSHIV: Perceived Stress Scale Among People Living With HIV and AIDS.

^av^ISCS: Impact on Self-Concept Scale.

^aw^IHIV: Impact of HIV.

^ax^HIVMS: HIV Meaningfulness Scale.

^ay^PLHIV-RS: People Living with HIV Resilience Scale.

^az^BIS: Body Image in Patients With HIV or AIDS.

^ba^OCLS: Owen Clinic Lipodystrophy Scale.

^bb^ACTG-ABCD: Adult AIDS Clinical Trial Group’s Assessment of Body Change and Distress.

^bc^ACTG-ABCD-SF: ACTG-ABCD Short Form.

^bd^FAI: Facial Appearance Inventory.

^be^MAS: Medication Attribution Scale.

^bf^HIVTSQ: HIV Treatment Satisfaction Questionnaire.

^bg^TES: Treatment-Related Empowerment Scale.

^bh^SIS: Subcutaneous Injection Survey.

^bi^QUOTE-HIV: quality of care through the patient’s eyes.

^bj^AHHCP: Attitudes Toward HIV Health Care Provider scale.

^bk^AGAS: Antiretroviral General Adherence Scale.

^bl^HCR: Health Care Relationship Trust Scale.

^bm^HMRS: HIV Medication Readiness Scale.

^bn^HTOS: HIV Treatment Optimism Scale.

^bo^HIV-MT-SES: HIV Medication Taking Self-Efficacy Scale.

^bp^BEHKA-HIV: Brief Estimate of Health Knowledge and Action-HIV version.

^bq^HTRM: HIV Treatment Readiness Measure.

^br^HTRFS: HIV Treatment Regimen Fatigue Scale.

^bs^HECCS: HIV Engagement in and Continuity of Care Scale.

^bt^SSI: Social Support Inventory.

^bu^USII-HIV: Unsupportive Social Interactions Inventory-HIV version.

^bv^PSS-HIV: Perceived Social Support for HIV.

^bw^HIV-ASES: HIV Treatment Adherence Self-Efficacy Scale.

^bx^PHIVSMS: Perceived HIV Self-Management Scale.

^by^HIV-SMS-W: HIV Self-Management Scale (Women).

^bz^HIV-IM: HIV Intention Measure.

^ca^HIVESS: HIV Exercise Stereotypes Scale.

^cb^HSM-SEWS: HIV Symptom Management Self-Efficacy for Women Scale.

## Discussion

### Principal Findings

From the 152 included studies, we identified 88 PROMs in 8 categories for adults living with HIV, and the psychometric properties of the majority of the included PROMs were rated with insufficient evidence. The principal finding of this review was the lack of comprehensively validated HIV-specific PROMs for the assessment of health outcomes in adults living with HIV and AIDS. Although 3 available PROMs (PozQoL, HIV-SI or SDM, and PLHIV-RS) have been recommended based on the COSMIN guidelines, they all have some shortcomings. In addition, because of limited evidence, recommendations regarding the use of most of the remaining assessed PROMs (class B recommendation) cannot be made. These findings emphasize on the need for a more comprehensive validation of the psychometric properties of the existing PROMs. Furthermore, our findings indicate the need for a robust and rapid validation of PROMs through the use of electronic PROMs (ePROMs) and modern measurement theories (such as Item Response Theory).

### Taxonomy of HIV-Specific PROMs

This systematic review updated the review reported by Engler [22] and provided improvisations on the inclusion and exclusion criteria, such that many unvalidated PROMs were excluded because if we include these PROMs, we cannot summarize the overall status of their psychometric properties. In addition, using the 12 categories reported by inductive content analysis in the review of Engler [22] as reference, this review reported 8 integrated categories (Table 1). The 2 categories of “ART and adherence-related views and experiences” and “healthcare-related views and experiences” in the study by Engler et al [22] were integrated into “treatment,” and “psychological challenges” and “psychological resources” were integrated into the category “psychological”; the PROMs in the “sexual and reproductive health” category were excluded because they did not meet the inclusion criteria for our study. Finally, the “Disability” category was integrated with “Symptoms.” The new taxonomy proposed in this review should be helpful for health care providers and researchers in selecting PROM.

In addition, although some of the PROMs included cognitive function or symptoms to some extent (such as “cognitive functioning” of Medical Outcomes Study-HIV Health Survey and “cognitive symptoms” of HIV Disability Questionnaire), no PROM specifically designed to measure cognitive concerns was included in the analysis. However, considering the high prevalence of HIV-associated neurocognitive disorders and HIV-associated dementia in people living with HIV and AIDS, it is important to assess their cognition via PROMs [184]. Askari [185,186] conducted a series of studies to progressively simplify the item pool and developed a PROM (the Communicating Cognitive Concerns Questionnaire) aimed at assessing the cognitive abilities of people living with HIV and AIDS. The main cognitive dimensions measured by this PROM included memory, concentration, executive function, language, emotions, and motivation. Although the Communicating Cognitive Concerns Questionnaire did not correlate strongly with cognitive test performance in people living with HIV and AIDS, it reflected the real-life concerns of people living with HIV and AIDS in terms of their mood, work, and work productivity. Although the related PROMs were not included in this review, we will further explore these cognitive concerns as an independent PROM category in future studies.

### Psychometric Properties

#### Overview

A thorough validation process is important for ensuring the applicability of a PROM to individual patient care [187]. However, in this review, most included PROMs were short of evidence for many psychometric properties, such as content validity, measurement error, cross-cultural validity or measurement invariance, and responsiveness. Therefore, it was difficult to assess the quality of these PROMs.

#### Content Validity

On the basis of the most up-to-date COSMIN methodology [26], content validity is the most important psychometric property, and the current guidance suggests that it is very important for patients to participate in development and validation studies [25]. As suggested by Selby and Velikova [188], and public involvement should appear as a core feature in PROM design and application. In addition, Wilson [189] believed that the perception of patients was essential for providing better insights into how a disease affects HRQoL. However, they were short of evidence in terms of patient and public involvement in the development process of the included PROMs. To determine whether a PROM was well designed, it should be confirmed that the PROM is relevant, comprehensible, and comprehensive from a patient perspective and for their context of use [190]. In addition, PROMs should be able to record the experience of people living with HIV and AIDS and how HIV affects their lives so as to make a study more relevant and have better content validity [191].

#### Internal Structure

Internal consistency was the most frequently reported psychometric property. However, many studies used internal consistency as the only indicator of reliability, which was definitely not enough. Besides, structural validity is also one of the most important psychometric properties [192]. The premise for assessing internal consistency is at least “low” evidence for “sufficient” structural validity, and this evidence may come from different studies [27]. However, only exploratory factor analysis was conducted in many studies for the assessment of structural validity instead of confirmatory factor analysis. Accordingly, this property can only obtain the rating of “indeterminate,” further affecting the assessment of internal consistency. In addition, the assessment of structural validity in most studies included in this review was based on classical test theory. Only 2 studies used Rasch analysis to assess the extent of interval level measurement and implementation of unidimensionality in this review [62,67]. However, no guidance has been provided in the COSMIN guidelines with regard to relying on only Rasch analysis without classical test theory statistics to assess the structural validity of PROMs. Therefore, Recchioni [193] suggested that it is necessary to provide additional guidance for the study that only uses Rasch analysis, especially in the development of new PROMs.

A PROM developed in one particular context may not be suitable for another. Therefore, it is necessary to use the same PROM for direct comparisons between different populations. No positive results for cross-cultural validity or measurement invariance were reported in this review [82,111,122,124,127], showing that the validity and transferability of the included PROMs between different geographies, cultural contexts, and risk populations were still unclear. Many researchers directly use the existing PROMs through simple translations and ignore cross-cultural adaptation [194]. However, there are great differences in the understanding of some concepts among people of different cultures, global regions, genders, ages, and socioeconomic strata [195]. The use of PROMs in different contexts is not simply dependent on translating items but should be processed based on a 7-key-step process for comprehensive cross-cultural adaptation [196].

#### Remaining Psychometric Properties

Measurement error was also important for interpreting PROs. Minimal important change is best calculated from multiple studies and using multiple anchors with an anchor-based longitudinal approach [197]. In this review, only 1 study reported the smallest detectable change ranging from 7.3 to 15.0 points without minimal important change. Therefore, measurement error was assessed as “indeterminate” [118]. Moreover, only few studies assessed responsiveness. However, responsiveness was vital to assess the effectiveness of a clinical intervention designed to improve the health outcomes of people living with HIV and AIDS. This identifies several gaps for future research in the area of HIV. Without such information, it is impossible to understand whether changes in the levels of health outcomes of people living with HIV and AIDS are meaningful and matter to health care providers and researchers.

### Clinical Implications

Despite a 64% reduction in HIV-related deaths in 2020 compared with the peak reported in 2004, a total of 680,000 people living with HIV and AIDS still died from HIV-related illnesses in 2020. This was largely due to the unique physical and psychosocial symptoms [1]. These symptoms seriously affect the physical function and clinical outcomes of people living with HIV and AIDS [4,198-200]. PRO data can be used in a variety of ways to improve care and health outcomes at a patient, institution, and population level [201-204]. Considering the particularity of people living with HIV and AIDS on subjective and privacy issues, PROs should be the primary outcome or end point. Many regulatory agencies and guidelines also recommend the inclusion of PROMs as the primary or secondary end points in clinical trials [205,206]. In addition, the development of the current ART regimen aims at simplifying the form of administration to meet the needs of long-term ART and maintain viral suppression with minimal toxicity [207]. Therefore, PRO data are becoming increasingly important for determining which ART regimen to use [208]. Therefore, a reliable, valid, and sensitive PROM is invaluable to health care providers and researchers.

In this systematic review, only 3 available PROMs (PozQoL, HIV-SI or SDM, and PLHIV-RS) were recommended based on the COSMIN guidelines, wherein PozQoL was used to assess HIV-related HRQoL, HIV-SI or SDM was used to assess HIV-related symptoms, and PLHIV-RS was used to assess HIV-related resilience. Health care providers can adopt these 3 PROMs for different application purposes. With regard to PROMs that received class B recommendation, although these PROMs are not recommended in this systematic review, researchers can select the PROMs with relatively good results for psychometric properties and use them according to the research purpose or further validate them for use in their context. For administrators, selecting validated PROMs can aid in the development of continuous quality improvement reports to understand health care providers’ performance against the measurement framework and standard key performance indicators [209]. On the basis of the data collected through validated PROMs, policy makers can further evaluate system performance by comparing outcomes over time and support health care policy decision-making [210]. In summary, this review will help health care providers, administrators, policy makers, and researchers to choose suitable PROMs in different contexts, which in turn will promote the systematic use of these PROMs, identify areas that need to be improved from a patient perspective, and improve the quality of assessment for intervention.

### Limitations

Our study has some limitations. First, although this systematic review additionally searched 2 important web-based databases of PROMs (PROQOLID and PROMIS) that are considered to be an important source of gray literature, we did not search dissertations, non-English literature, and other gray literature. This may have caused some relevant studies to be left out of our analysis, and these studies may help provide some evidence to support or refute our findings. Furthermore, evidence on the validation of PROMs can be deduced from the results of some studies. However, it was not the primary purpose of these studies; therefore, these studies were not included. Furthermore, some other PROMs were not included because they are still under study. Moreover, this systematic review may have ignored PROMs that only assessed a certain domain related to specific comorbidities, such as PROMs specifically designed to measure cognitive concerns. Considering the importance of evaluating these comorbidities in people living with HIV and AIDS, we will conduct further research on these PROMs. Furthermore, because no generally accepted “golden standard” measure for adults living with HIV and AIDS currently exists, the criterion validity of the included PROMs was not assessed. In addition, an insufficient number of studies reporting PROM development and content validity were included in this systematic review. Although we excluded many qualitative studies during the title and abstract screening stage, none of these studies researched on content validity. However, this is the same as the other relevant reviews [16,19] that also searched for insufficient studies reporting on the content validity of HIV-related PROMs.

One another limitation of this review is that the selection of studies, scoring of methodological quality, and grading of evidence were subjective in nature. However, this systematic review strictly followed the steps of the COSMIN guidelines, and the processes mentioned earlier involved multiple researchers. We believe that this could resolve discrepancies and reduce variability in interpretation, thereby minimizing the chance of errors. Furthermore, given that the negative results of many PROMs are less likely to be published, the possibility of publication bias cannot be eliminated. Moreover, some included studies may have reported on only some psychometric properties; accordingly, there may be a selective reporting bias. Finally, quantitative pooled summary or meta-analyses were not performed because of the possible large heterogeneity. These limitations may help to explain why concrete recommendations for the use of some PROMs were not made because there were few included studies for some PROMs, and not all psychometric properties were assessed in these studies.

### Future Work

Although there are a large number of PROMs in each category, it would be necessary to validate the existing PROMs, or even develop new PROMs in some categories, because not enough validated PROMs are available. Considering the shortcomings of the 3 class A PROMs, efforts in future research should focus on validation as well as class B PROMs. It should be noted that multiple personnel such as patients themselves, their family members, health care providers, and researchers should participate in the development and validation of all PROMs [211]. In the future research on PROMs, researchers should follow the suggestions of the COSMIN guidelines to ensure the complete reporting of research details and accurate interpretation of results [27].

For the existing PROMs, research should focus on the validation of content validity and measurement error to determine the suitability of a PROM for use in the care of people living with HIV and AIDS. Moreover, these PROMs should be applied to different regions or populations to assess their cross-cultural validity or measurement invariance and explore the comparability of the results. In addition, future research should use more longitudinal or experimental study designs to assess the responsiveness of PROMs [9].

With the gradual aging of people living with HIV and AIDS, new and adjusted PROMs should focus on exploring the impact of aging on people living with HIV and AIDS, such as complex complications [212], polypharmacy [213], menopause in older women [214], low social support [215], cognitive impairment [216], and special symptoms of early exposure to HIV [9]. PROMs for children will be summarized in our future research.

In the past decades, researchers have mainly used interviewer-administered surveys and self-administered paper questionnaires to collect data [217]. However, several limitations of these methods have been found in the actual application process. ePROMs are becoming increasingly popular in recent years, greatly saving labor and time costs, minimizing errors, and realizing complex survey management [9]. Despite the fact that ePROMs are rapidly developing, future research should pay attention to evaluating the equivalence between electronic questionnaires and paper questionnaires [218]. Some researchers have used the most advanced technologies to integrate ePROMs into electronic hospital records or routine HIV care, allowing health care providers to easily and conveniently assess the qualitative and quantitative health outcomes of people living with HIV and AIDS. In addition, there are independent apps and software used in clinical practice and research.

Moreover, with the development of computer adaptive tests (CATs) in recent years, future research can develop and improve the item bank for people living with HIV and AIDS and use the CAT technology to dynamically select items for administration based on the respondent’s previous answers for finally assessing their PROs [219-221]. However, the item bank of the CAT instrument requires a large number of unidimensional scales, posing a great challenge to the content validity of each PROM and its subconstructs. At the same time, the development of a CAT item bank can promote the improvement of the existing HIV-specific PROMs and the development of new HIV-specific PROMs, further promoting the vigorous development of research in related fields in the future.

### Conclusions

This systematic review provides a detailed assessment of the psychometric properties of the existing HIV-specific PROMs for adults living with HIV and AIDS. Class A rating of PROMs was achieved for PozQoL, HIV-SI or SDM, and PLHIV-RS. However, all of these have a few shortcomings. Therefore, this study believes that future studies should conduct a more comprehensive validation of the psychometric properties of the existing PROMs to provide sufficient assessment evidence. These findings may provide a reference for the selection of high-quality HIV-specific PROMs by health care providers and researchers for clinical practice and research.

## Appendix

Multimedia Appendix 1 Literature search strategy for existing review, literature search strategy for HIV-specific patient-reported outcome measures, subscales of the included PROMs, characteristics of the included records, methodological quality assessment of the included records, results and ratings of each psychometric property of each record, the overall results and the level of evidence, PRISMA (Preferred Reporting Items for Systematic Reviews and Meta-Analyses) 2020 abstract checklist, and PRISMA 2020 main checklist.

## Abbreviations

ART: antiretroviral therapy
CAT: computer adaptive test
COSMIN: Consensus-Based Standards for the Selection of Health Measurement Instruments
ePROM: electronic patient-reported outcome measure
HIV-SI or SDM: HIV Symptom Index or Symptoms Distress Module of the Adult AIDS Clinical Trial Group
HRQoL: health-related quality of life
PLHIV-RS: People Living with HIV Resilience Scale
PozQoL: Poz Quality of Life
PRISMA: Preferred Reporting Items for Systematic Reviews and Meta-Analyses
PRO: patient-reported outcome
PROM: patient-reported outcome measure
