# Supplementary material for: HIV-Specific Reported Outcome Measures: Systematic Review of Psychometric Properties
Source: JMIR Public Health Surveill. 2022 Dec 8;8(12):e39015. doi: 10.2196/39015 (PMC9782451; doi:10.2196/39015)
Supplement: Multimedia Appendix 1 [file publichealth_v8i12e39015_app1.docx]

# Multimedia Appendix Content

Table S1. Literature Search Strategy for Existing Review

Table S2. Literature Search Strategy for HIV-Specific patient-reported outcome measures (PROMs)

Table S3. Subscales of the included PROMs.

Table S4. Characteristics of the Included Records

Table S5. Methodological Quality Assessment of the Included Records

Table S6. Results and Ratings of Each Psychometric Property of Each Record

Table S7. The Overall Results and the Level of Evidence

Table S8. PRISMA 2020 Abstract Checklist

Table S9. PRISMA 2020 Main Checklist

This Multimedia Appendix has been provided by the authors to give readers additional information about their work.

Please note: The publisher is not responsible for the content or functionality of any Multimedia Appendix supplied by the authors. Any queries (other than missing content) should be directed to the corresponding author for the article.

Article title: HIV-Specific Patient Reported Outcome Measures: Systematic Review of Psychometric Properties

Journal name: JMIR Public Health & Surveillance

Author names: Ziqi Wang Yaxin Zhu, Xiyu Duan, Hao Kang, Bo Qu

Corresponding author: Professor. Bo Qu

Affiliation: School of Public Health, China Medical University, Shenyang, China

E-mail address: qubo6666@163.com

## Table S1. Literature Search Strategy for Existing Review

MEDLINE (via Pubmed) database — 242 records

| **Terms** | **Strategy** |
| --- | --- |
| **Condition of Interest** | HIV[MESH] OR AIDS[MESH] OR “People living with HIV/AIDS”[tiab] OR “People living with HIV”[tiab] OR “HIV positive”[tiab] |
| **AND** | |
| **Construct of Interest** | “Quality of life”[MESH] OR “life quality”[tw] OR "Health related quality of life"[tiab] OR QoL[tiab] OR QL[tiab] OR HRQOL[tiab] OR HR-QOL[tiab] OR HRQL[tiab] OR “Patient Reported Outcome Measures”[MESH] OR "Health Related Patient Reported Outcomes"[tiab] OR HR-PRO[tiab] OR “Personal Satisfaction”[MESH] OR "Life satisfaction"[tiab] OR “health index*”[tiab] OR “health indices”[tiab] OR “health profile*”[tiab] OR “health status”[tw] OR ((disability[tiab] OR function[tiab] OR functional[tiab] OR functions[tiab] OR subjective[tiab] OR utility[tiab] OR utilities[tiab] OR wellbeing[tiab] OR “well-being”[tiab]) AND (index[tiab] OR indices[tiab] OR instrument[tiab] OR instruments[tiab] OR measure[tiab] OR measures[tiab] OR questionnaire[tiab] OR questionnaires[tiab] OR profile[tiab] OR profiles[tiab] OR scale[tiab] OR scales[tiab] OR score[tiab] OR scores[tiab] OR survey[tiab] OR surveys[tiab] OR status[tiab])) |
| **AND** | |
| **Psychometric Properties** | Instrumentation[sh] OR methods[sh] OR “Validation Studies”[pt] OR “Comparative Study”[pt] OR “Psychometrics”[MESH] OR psychometr*[tiab] OR clinimetr*[tw] OR clinometr*[tw] OR "outcome assessment (health care)"[MESH] OR “outcome assessment”[tiab] OR “outcome measure*”[tw] OR “observer variation”[MESH] OR “observer variation”[tiab] OR “Health Status Indicators”[MESH] OR “reproducibility of results”[MESH] OR reproducib*[tiab] OR “discriminant analysis”[MESH] OR reliab*[tiab] OR unreliab*[tiab] OR valid*[tiab] OR "coefficient of variation"[tiab] OR coefficient[tiab] OR homogeneity[tiab] OR homogeneous[tiab] OR “internal consistency”[tiab] OR (cronbach*[tiab] AND (alpha[tiab] OR alphas[tiab])) OR (item[tiab] AND (correlation*[tiab] OR selection*[tiab] OR reduction*[tiab])) OR agreement[tiab] OR precision[tiab] OR imprecision[tiab] OR “precise values”[tiab] OR test-retest[tiab] OR (test[tiab] AND retest[tiab]) OR (reliab*[tiab] AND (test[tiab] OR retest[tiab])) OR stability[tiab] OR interrater[tiab] OR inter-rater[tiab] OR intrarater[tiab] OR intra-rater[tiab] OR intertester[tiab] OR inter-tester[tiab] OR intratester[tiab] OR intra-tester[tiab] OR interobserver[tiab] OR inter-observer[tiab] OR intraobserver[tiab] OR intraobserver[tiab] OR intertechnician[tiab] OR inter-technician[tiab] OR intratechnician[tiab] OR intra-technician[tiab] OR interexaminer[tiab] OR inter-examiner[tiab] OR intraexaminer[tiab] OR intra-examiner[tiab] OR interassay[tiab] OR inter-assay[tiab] OR intraassay[tiab] OR intra-assay[tiab] OR interindividual[tiab] OR inter-individual[tiab] OR intraindividual[tiab] OR intra-individual[tiab] OR interparticipant[tiab] OR inter-participant[tiab] OR intraparticipant[tiab] OR intra-participant[tiab] OR kappa[tiab] OR kappa’s[tiab] OR kappas[tiab] OR repeatab*[tiab] OR ((replicab*[tiab] OR repeated[tiab]) AND (measure[tiab] OR measures[tiab] OR findings[tiab] OR result[tiab] OR results[tiab] OR test[tiab] OR tests[tiab])) OR generaliza*[tiab] OR generalisa*[tiab] OR concordance[tiab] OR (intraclass[tiab] AND correlation*[tiab]) OR discriminative[tiab] OR “known group”[tiab] OR “factor analysis”[tiab] OR “factor analyses”[tiab] OR "factor structure"[tiab] OR "factor structures"[tiab] OR "interval variability"[tiab] OR "rate variability"[tiab] OR dimension*[tiab] OR subscale*[tiab] OR (multitrait[tiab] AND scaling[tiab] AND (analysis[tiab] OR analyses[tiab])) OR “item discriminant”[tiab] OR “interscale correlation*”[tiab] OR error[tiab] OR errors[tiab] OR “individual variability”[tiab] OR (variability[tiab] AND (analysis[tiab] OR values[tiab])) OR (uncertainty[tiab] AND (measurement[tiab] OR measuring[tiab])) OR “standard error of measurement”[tiab] OR sensitiv*[tiab] OR responsive*[tiab] OR (limit[tiab] AND detection[tiab]) OR "minimal detectable concentration"[tiab] OR interpretab*[tiab] OR ((minimal[tiab] OR minimally[tiab] OR clinical[tiab] OR clinically[tiab]) AND (important[tiab] OR significant[tiab] OR detectable[tiab]) AND (change[tiab] OR difference[tiab])) OR (small*[tiab] AND (real[tiab] OR detectable[tiab]) AND (change[tiab] OR difference[tiab])) OR “meaningful change”[tiab] OR “ceiling effect”[tiab] OR “floor effect”[tiab] OR “Item response model”[tiab] OR IRT[tiab] OR Rasch[tiab] OR “Differential item functioning”[tiab] OR DIF[tiab] OR “computer adaptive testing”[tiab] OR “item bank”[tiab] OR “cross-cultural equivalence”[tiab] |
| **AND** | |
| **Article type** | (Review[Publication Type]) OR (Systematic Review[Publication Type]) OR (Meta-Analysis[Publication Type]) |

## Table S2. Literature Search Strategy for HIV-Specific PROMs

1. MEDLINE (via Pubmed) database — 2736 records

| **Terms** | **Strategy** |
| --- | --- |
| **Condition of Interest** | HIV[MESH] OR AIDS[MESH] OR “People living with HIV/AIDS”[tiab] OR “People living with HIV”[tiab] OR “HIV positive”[tiab] |
| **AND** | |
| **Construct of Interest** | “Quality of life”[MESH] OR “life quality”[tw] OR "Health related quality of life"[tiab] OR QoL[tiab] OR QL[tiab] OR HRQOL[tiab] OR HR-QOL[tiab] OR HRQL[tiab] OR “Patient Reported Outcome Measures”[MESH] OR "Health Related Patient Reported Outcomes"[tiab] OR HR-PRO[tiab] OR “Personal Satisfaction”[MESH] OR "Life satisfaction"[tiab] OR “health index*”[tiab] OR “health indices”[tiab] OR “health profile*”[tiab] OR “health status”[tw] OR ((disability[tiab] OR function[tiab] OR functional[tiab] OR functions[tiab] OR subjective[tiab] OR utility[tiab] OR utilities[tiab] OR wellbeing[tiab] OR “well-being”[tiab]) AND (index[tiab] OR indices[tiab] OR instrument[tiab] OR instruments[tiab] OR measure[tiab] OR measures[tiab] OR questionnaire[tiab] OR questionnaires[tiab] OR profile[tiab] OR profiles[tiab] OR scale[tiab] OR scales[tiab] OR score[tiab] OR scores[tiab] OR survey[tiab] OR surveys[tiab] OR status[tiab])) |
| **AND** | |
| **Psychometric Properties** | Instrumentation[sh] OR methods[sh] OR “Validation Studies”[pt] OR “Comparative Study”[pt] OR “Psychometrics”[MESH] OR psychometr*[tiab] OR clinimetr*[tw] OR clinometr*[tw] OR "outcome assessment (health care)"[MESH] OR “outcome assessment”[tiab] OR “outcome measure*”[tw] OR “observer variation”[MESH] OR “observer variation”[tiab] OR “Health Status Indicators”[MESH] OR “reproducibility of results”[MESH] OR reproducib*[tiab] OR “discriminant analysis”[MESH] OR reliab*[tiab] OR unreliab*[tiab] OR valid*[tiab] OR "coefficient of variation"[tiab] OR coefficient[tiab] OR homogeneity[tiab] OR homogeneous[tiab] OR “internal consistency”[tiab] OR (cronbach*[tiab] AND (alpha[tiab] OR alphas[tiab])) OR (item[tiab] AND (correlation*[tiab] OR selection*[tiab] OR reduction*[tiab])) OR agreement[tiab] OR precision[tiab] OR imprecision[tiab] OR “precise values”[tiab] OR test-retest[tiab] OR (test[tiab] AND retest[tiab]) OR (reliab*[tiab] AND (test[tiab] OR retest[tiab])) OR stability[tiab] OR interrater[tiab] OR inter-rater[tiab] OR intrarater[tiab] OR intra-rater[tiab] OR intertester[tiab] OR inter-tester[tiab] OR intratester[tiab] OR intra-tester[tiab] OR interobserver[tiab] OR inter-observer[tiab] OR intraobserver[tiab] OR intraobserver[tiab] OR intertechnician[tiab] OR inter-technician[tiab] OR intratechnician[tiab] OR intra-technician[tiab] OR interexaminer[tiab] OR inter-examiner[tiab] OR intraexaminer[tiab] OR intra-examiner[tiab] OR interassay[tiab] OR inter-assay[tiab] OR intraassay[tiab] OR intra-assay[tiab] OR interindividual[tiab] OR inter-individual[tiab] OR intraindividual[tiab] OR intra-individual[tiab] OR interparticipant[tiab] OR inter-participant[tiab] OR intraparticipant[tiab] OR intra-participant[tiab] OR kappa[tiab] OR kappa’s[tiab] OR kappas[tiab] OR repeatab*[tiab] OR ((replicab*[tiab] OR repeated[tiab]) AND (measure[tiab] OR measures[tiab] OR findings[tiab] OR result[tiab] OR results[tiab] OR test[tiab] OR tests[tiab])) OR generaliza*[tiab] OR generalisa*[tiab] OR concordance[tiab] OR (intraclass[tiab] AND correlation*[tiab]) OR discriminative[tiab] OR “known group”[tiab] OR “factor analysis”[tiab] OR “factor analyses”[tiab] OR "factor structure"[tiab] OR "factor structures"[tiab] OR "interval variability"[tiab] OR "rate variability"[tiab] OR dimension*[tiab] OR subscale*[tiab] OR (multitrait[tiab] AND scaling[tiab] AND (analysis[tiab] OR analyses[tiab])) OR “item discriminant”[tiab] OR “interscale correlation*”[tiab] OR error[tiab] OR errors[tiab] OR “individual variability”[tiab] OR (variability[tiab] AND (analysis[tiab] OR values[tiab])) OR (uncertainty[tiab] AND (measurement[tiab] OR measuring[tiab])) OR “standard error of measurement”[tiab] OR sensitiv*[tiab] OR responsive*[tiab] OR (limit[tiab] AND detection[tiab]) OR "minimal detectable concentration"[tiab] OR interpretab*[tiab] OR ((minimal[tiab] OR minimally[tiab] OR clinical[tiab] OR clinically[tiab]) AND (important[tiab] OR significant[tiab] OR detectable[tiab]) AND (change[tiab] OR difference[tiab])) OR (small*[tiab] AND (real[tiab] OR detectable[tiab]) AND (change[tiab] OR difference[tiab])) OR “meaningful change”[tiab] OR “ceiling effect”[tiab] OR “floor effect”[tiab] OR “Item response model”[tiab] OR IRT[tiab] OR Rasch[tiab] OR “Differential item functioning”[tiab] OR DIF[tiab] OR “computer adaptive testing”[tiab] OR “item bank”[tiab] OR “cross-cultural equivalence”[tiab] |

2. Embase database — 7322 records

| **Terms** | **Strategy** |
| --- | --- |
| **Condition of Interest** | 'human immunodeficiency virus infection'/exp OR 'human immunodeficiency virus infection' OR 'acquired immune deficiency syndrome'/exp OR 'acquired immune deficiency syndrome' OR 'aids patient'/exp OR 'aids patient' OR 'human immunodeficiency virus infected patient'/exp OR 'human immunodeficiency virus infected patient' OR 'people living with hiv/aids'/exp OR 'people living with hiv/aids' OR 'hiv positive' |
| **AND** | |
| **Construct of Interest** | 'quality of life'/exp OR 'quality of life' OR 'life quality'/exp OR 'life quality' OR 'health related quality of life':ab,ti OR 'health-related quality of life':ab,ti OR 'patient reported outcome measures'/exp OR 'patient reported outcome measures' OR 'patient reported outcomes'/exp OR 'patient reported outcomes' OR 'health related patient reported outcomes' OR 'satisfaction' OR 'satisfaction'/exp OR satisfaction OR 'life satisfaction':ab,ti OR 'health index*':ab,ti OR 'health indices':ab,ti OR 'health profile*':ab,ti OR 'health status'/exp OR 'health status' OR ((disability:ab,ti OR function*:ab,ti OR subjective:ab,ti OR utility:ab,ti OR utilities:ab,ti OR wellbeing:ab,ti OR 'well being':ab,ti) AND (index:ab,ti OR indices:ab,ti OR instrument*:ab,ti OR measure*:ab,ti OR questionnaire*:ab,ti OR profile*:ab,ti OR scale*:ab,ti OR score*:ab,ti OR survey*:ab,ti OR status:ab,ti)) |
| **AND** | |
| **Psychometric Properties** | instrumentation:ti OR methods:ti OR 'psychometry' OR 'psychometry'/exp OR psychometry OR psychometr*:ab,ti OR clinimetr* OR clinometr* OR 'outcome assessment':ab,ti OR 'outcome assessment (health care)'/exp OR 'outcome assessment (health care)' OR 'outcome measure*' OR 'observer variation'/exp OR 'observer variation' OR 'observer variation':ab,ti OR 'health status indicator'/exp OR 'health status indicator' OR 'reproducibility of results'/exp OR 'reproducibility of results' OR reproducib*:ab,ti OR 'discriminant analysis'/exp OR 'discriminant analysis' OR reliab*:ab,ti OR unreliab*:ab,ti OR valid*:ab,ti OR 'coefficient of variation':ab,ti OR coefficient:ab,ti OR homogeneity:ab,ti OR homogeneous:ab,ti OR 'internal consistency':ab,ti OR (cronbach*:ab,ti AND (alpha:ab,ti OR alphas:ab,ti)) OR (item:ab,ti AND (correlation*:ab,ti OR selection*:ab,ti OR reduction*:ab,ti)) OR agreement:ab,ti OR precision:ab,ti OR imprecision:ab,ti OR 'precise values':ab,ti OR 'test retest reliability':ab,ti OR (test:ab,ti AND retest:ab,ti) OR (reliab*:ab,ti AND (test:ab,ti OR retest:ab,ti)) OR stability:ab,ti OR interrater:ab,ti OR 'inter-rater':ab,ti OR intrarater:ab,ti OR 'intra-rater':ab,ti OR intertester:ab,ti OR 'inter-tester':ab,ti OR intratester:ab,ti OR 'intra-tester':ab,ti OR interobserver:ab,ti OR 'inter observer':ab,ti OR intraobserver:ab,ti OR intertechnician:ab,ti OR 'inter-technician':ab,ti OR intratechnician:ab,ti OR 'intra-technician':ab,ti OR interexaminer:ab,ti OR 'inter-examiner':ab,ti OR intraexaminer:ab,ti OR 'intra-examiner':ab,ti OR interassay:ab,ti OR 'inter-assay':ab,ti OR intraassay:ab,ti OR 'intra-assay':ab,ti OR interindividual:ab,ti OR 'inter-individual':ab,ti OR intraindividual:ab,ti OR 'intra-individual':ab,ti OR interparticipant:ab,ti OR 'inter-participant':ab,ti OR intraparticipant:ab,ti OR 'intra-participant':ab,ti OR kappa*:ab,ti OR 'kappa statistics':ab,ti OR repeatab*:ab,ti OR ((replicab*:ab,ti OR repeated:ab,ti) AND (measure*:ab,ti OR findings:ab,ti OR result*:ab,ti OR test*:ab,ti)) OR generaliza*:ab,ti OR generalisa*:ab,ti OR concordance:ab,ti OR (intraclass:ab,ti AND correlation*:ab,ti) OR discriminative:ab,ti OR 'known group':ab,ti OR 'factor analysis':ab,ti OR 'factor analyses':ab,ti OR 'factor structure':ab,ti OR 'factor structures':ab,ti OR 'interval variability':ab,ti OR 'rate variability':ab,ti OR dimension*:ab,ti OR subscale*:ab,ti OR (multitrait:ab,ti AND scaling:ab,ti AND (analysis:ab,ti OR analyses:ab,ti)) OR 'item discriminant':ab,ti OR 'interscale correlation*':ab,ti OR error*:ab,ti OR 'individual variability':ab,ti OR (variability:ab,ti AND (analysis:ab,ti OR values:ab,ti)) OR (uncertainty:ab,ti AND (measurement:ab,ti OR measuring:ab,ti)) OR 'standard error of measurement':ab,ti OR sensitiv*:ab,ti OR responsive*:ab,ti OR (limit:ab,ti AND detection:ab,ti) OR 'minimal detectable concentration':ab,ti OR interpretab*:ab,ti OR ((minimal:ab,ti OR minimally:ab,ti OR clinical:ab,ti OR clinically:ab,ti) AND (important:ab,ti OR significant:ab,ti OR detectable:ab,ti) AND (change:ab,ti OR difference:ab,ti)) OR (small*:ab,ti AND (real:ab,ti OR detectable:ab,ti) AND (change:ab,ti OR difference:ab,ti)) OR 'meaningful change':ab,ti OR 'ceiling effect':ab,ti OR 'floor effect':ab,ti OR 'item response model':ab,ti OR irt:ab,ti OR rasch:ab,ti OR 'differential item functioning':ab,ti OR dif:ab,ti OR 'computer adaptive testing':ab,ti OR 'item bank':ab,ti OR 'cross-cultural equivalence':ab,ti |

3. PsycInfo (via EBSCO) database — 1303 records

| **Terms** | **Strategy** |
| --- | --- |
| **Condition of Interest** | TX (“Human Immunodeficiency Virus” OR “acquired immunodeficiency syndrome” OR “People living with HIV/AIDS” OR “People living with HIV” OR “HIV positive”) |
| **AND** | |
| **Construct of Interest** | TX (“Quality of life” OR “life quality” OR "Health related quality of life" OR "Health-related quality of life" OR QoL OR QL OR HRQOL OR HR-QOL OR HRQL OR “Patient Reported Outcome Measures” OR “Patient Reported Outcomes” OR "Health Related Patient Reported Outcomes" OR "Personal Satisfaction" OR "Life satisfaction" OR “health index*” OR “health indices” OR “health profile*” OR “health status” OR ((disability OR function OR functional OR functions OR subjective OR utility OR utilities OR wellbeing OR “well being”) AND (index OR indices OR instrument OR instruments OR measure OR measures OR questionnaire OR questionnaires OR profile OR profiles OR scale OR scales OR score OR scores OR survey OR surveys OR status))) |
| **AND** | |
| **Psychometric Properties** | AB (Instrumentation OR methods OR “Validation Studies” OR “Comparative Study” OR “Psychometrics” OR psychometr* OR clinimetr* OR clinometr* OR “outcome assessment (Health Care)” OR “outcome assessment” OR “outcome measure*” OR “observer variation” OR “observer variation” OR “Health Status Indicators” OR “reproducibility of results” OR reproducib* OR “discriminant analysis” OR reliab* OR unreliab* OR valid* OR "coefficient of variation" OR coefficient OR homogeneity OR homogeneous OR “internal consistency” OR (cronbach* AND (alpha OR alphas)) OR (item AND (correlation* OR selection* OR reduction*)) OR agreement OR precision OR imprecision OR “precise values” OR test-retest OR (test AND retest) OR (reliab* AND (test OR retest)) OR stability OR interrater OR inter-rater OR intrarater OR intra-rater OR intertester OR inter-tester OR intratester OR intra-tester OR interobserver OR inter-observer OR intraobserver OR intraobserver OR intertechnician OR inter-technician OR intratechnician OR intra-technician OR interexaminer OR inter-examiner OR intraexaminer OR intra-examiner OR interassay OR inter-assay OR intraassay OR intra-assay OR interindividual OR inter-individual OR intraindividual OR intra-individual OR interparticipant OR inter-participant OR intraparticipant OR intra-participant OR kappa OR kappa’s OR kappas OR repeatab* OR ((replicab* OR repeated) AND (measure OR measures OR findings OR result OR results OR test OR tests)) OR generaliza* OR generalisa* OR concordance OR (intraclass AND correlation*) OR discriminative OR “known group” OR “factor analysis” OR “factor analyses” OR "factor structure" OR "factor structures" OR "interval variability" OR "rate variability" OR dimension* OR subscale* OR (multitrait AND scaling AND (analysis OR analyses)) OR “item discriminant” OR “interscale correlation*” OR error OR errors OR “individual variability” OR (variability AND (analysis OR values)) OR (uncertainty AND (measurement OR measuring)) OR “standard error of measurement” OR sensitiv* OR responsive* OR (limit AND detection) OR "minimal detectable concentration" OR interpretab* OR ((minimal OR minimally OR clinical OR clinically) AND (important OR significant OR detectable) AND (change OR difference)) OR (small* AND (real OR detectable) AND (change OR difference)) OR “meaningful change” OR “ceiling effect” OR “floor effect” OR “Item response model” OR IRT OR Rasch OR “Differential item functioning” OR DIF OR “computer adaptive testing” OR “item bank” OR “cross-cultural equivalence” ) OR TI ( Instrumentation OR methods OR “Validation Studies” OR “Comparative Study” OR “Psychometrics” OR psychometr* OR clinimetr* OR clinometr* OR “outcome assessment (Health Care)” OR “outcome assessment” OR “outcome measure*” OR “observer variation” OR “observer variation” OR “Health Status Indicators” OR “reproducibility of results” OR reproducib* OR “discriminant analysis” OR reliab* OR unreliab* OR valid* OR "coefficient of variation" OR coefficient OR homogeneity OR homogeneous OR “internal consistency” OR (cronbach* AND (alpha OR alphas)) OR (item AND (correlation* OR selection* OR reduction*)) OR agreement OR precision OR imprecision OR “precise values” OR test-retest OR (test AND retest) OR (reliab* AND (test OR retest)) OR stability OR interrater OR inter-rater OR intrarater OR intra-rater OR intertester OR inter-tester OR intratester OR intra-tester OR interobserver OR inter-observer OR intraobserver OR intraobserver OR intertechnician OR inter-technician OR intratechnician OR intra-technician OR interexaminer OR inter-examiner OR intraexaminer OR intra-examiner OR interassay OR inter-assay OR intraassay OR intra-assay OR interindividual OR inter-individual OR intraindividual OR intra-individual OR interparticipant OR inter-participant OR intraparticipant OR intra-participant OR kappa OR kappa’s OR kappas OR repeatab* OR ((replicab* OR repeated) AND (measure OR measures OR findings OR result OR results OR test OR tests)) OR generaliza* OR generalisa* OR concordance OR (intraclass AND correlation*) OR discriminative OR “known group” OR “factor analysis” OR “factor analyses” OR "factor structure" OR "factor structures" OR "interval variability" OR "rate variability" OR dimension* OR subscale* OR (multitrait AND scaling AND (analysis OR analyses)) OR “item discriminant” OR “interscale correlation*” OR error OR errors OR “individual variability” OR (variability AND (analysis OR values)) OR (uncertainty AND (measurement OR measuring)) OR “standard error of measurement” OR sensitiv* OR responsive* OR (limit AND detection) OR "minimal detectable concentration" OR interpretab* OR ((minimal OR minimally OR clinical OR clinically) AND (important OR significant OR detectable) AND (change OR difference)) OR (small* AND (real OR detectable) AND (change OR difference)) OR “meaningful change” OR “ceiling effect” OR “floor effect” OR “Item response model” OR IRT OR Rasch OR “Differential item functioning” OR DIF OR “computer adaptive testing” OR “item bank” OR “cross-cultural equivalence”) |

## Table S3. Subscales of the included patient-reported outcome measures (PROMs)^a,b^.

| PROM; year of development | | Targeted concept | Subscale (no. of items in each subscale) | Total no. of items |  |
| --- | --- | --- | --- | --- | --- |
| **HRQoL^c^** | | | | | |
|  | MOS-HIV^d^ [31-51]; 1996 | HRQoL | - Quality of life (1); General health perceptions (5); Physical functioning (6); Role functioning (2); Pain (2); Energy or fatigue (4); Health distress (4); Mental health (5); Cognitive functioning (4); Social functioning (1); Health transition (1) | 35 |  |
|  | MOS-HIV-17 [53]; 2000 | HRQoL | - Quality of life (1); Physical functioning (6); Role functioning (1); Health distress (2); Mental health (4); Cognitive functioning (2); Social functioning (1) | 17 |  |
|  | MOS-HIV-29 [52]; 2012 | HRQoL | - Physical functioning (6); Role function (1); General health perceptions (1); Bodily pain (2); Health transition (3); Mental health (5); Cognitive function (4); Health distress (2); Social function (1); Vitality (4) | 29 |  |
|  | HIV Overview of Problems Evaluation System [54,55]; 1992 | HRQoL | - Ambulation; Activities of daily living; Recreational activities; Weight loss; Difficulty working; Pain; Clothing; Symptoms; Problem obtaining information from medical team; Difficulty communicating with medical team; Control of medical team; Body image; Stigma; Psychological distress; Cognitive problems; Difficulty communicating with friends or relatives; Friends’ or relatives’ difficulty interacting; Anxiety in medical situations; Worry; Interaction with children; At-work concerns; Sexual interest; Sexual dysfunction; Communication with partners; Affection with partners; Interaction with partners; Overprotection by partners; Neglect of care by partners; Compliance; Concrete barriers; Looking for work; New relationships; Chemotherapy problems; Radiation therapy problems; Antiretroviral therapy problems | 165 |  |
|  | HIV-Related Quality of Life Questions [56]; 1993 | HRQoL | - Basic activities of daily living (3); Intermediate activities of daily living (3); mental health (5); Total physical symptoms (14); Extreme pain (1); Self-perceived memory problems (2); Fatigue (4); Disability days (2) | 34 |  |
|  | AIDS Health Assessment Questionnaire [57]; 1997 | HRQoL | - Disability (23); Energy (5); General health (2); Pain (1); Cognitive functioning (5); mental health (5); Social functioning (3); Health distress (4); Symptoms (68) | 116 |  |
|  | HIV-PARSE^e^ [58]; 1994 | HRQoL | - Current health perceptions (5); Physical functioning (6); Pain (1); Energy or fatigue (4); Emotional well-being (5); Cognitive functioning or distress (6); Role functioning (2); Social functioning (1) | 30 |  |
|  | HIV-PARSE-Brief [59]; 1995 | HRQoL | - Current health perceptions (3); physical functioning (4); Pain (2); Energy or fatigue (2); Emotional well-being (3); Cognitive functioning or distress (3); Role functioning (2); Social functioning (2) | 21 |  |
|  | HRQoL [60]; 1995 | HRQoL | - Health perception (5); Social function (2); Energy or fatigue (5); Physical function (6); Role function (2); Freedom from pain (2); Overall quality of life (3); Emotional well-being (5); Hopefulness (11); Freedom from loneliness (8); Will to function (4); Cognitive function or distress (6); Other items (5) | 64 |  |
|  | Functional Assessment of HIV Infection [61-65]; 1996 | HRQoL | - Physical well-being (10); Functional and global well-being (13); Emotional well-being or living with HIV (10); Social or family well-being (8); Cognitive functioning (3) | 44 |  |
|  | General Health Self-Assessment [66]; 1997 | HRQoL | - Health perceptions (5); physical functioning, including measures of intermediate activities of daily living (3) and basic activities of daily living (3); Psychological functioning, including measures of Mental health (5), Fatigue (4), and Cognitive impairment (3); Social functioning (3); Symptoms (18) | 49 |  |
|  | HIV Quality of Life 31-item scale [67]; 1997 | HRQoL | - Unidimensional scale (31-item scale) |  |  |
|  | HAT-QoL^f^-42 [68,69]; 1997 | HRQoL | Overall function (7); sexual function (3); disclosure worries (5); health worries (5); financial worries (4); HIV mastery (3); life satisfaction (8); medication concerns (4); provider trust; (3); | 42 |  |
|  | HAT-QoL-30 [35]; 1999 | HRQoL | - Overall function (6); sexual function (3); disclosure worries (5); health worries (3); financial worries (3); HIV mastery (2); life satisfaction (4); medication concerns (2); provider trust (2); | 30 |  |
|  | HAT-QoL-34 [42,70,71]; 2008 | HRQoL | - Overall function (6); sexual function (2); disclosure worries (5); health worries (4); financial worries (3); HIV mastery (2); life satisfaction (4); medication concerns (5); provider trust (3); | 34 |  |
|  | MQoL^g^ for Patients with HIV or AIDS [34,72-75]; 1997 | HRQoL | - Mental health (4); Physical health (4); physical functioning (4); Social functioning (4); Social support (4); Cognitive functioning (4); Financial status (4); Partner intimacy (4); Sexual functioning (4); Medical service (4) | 40 |  |
|  | Living with HIV Scale [76]; 1998 | HRQoL | - Avoiding the fear zone (6); Cherishing the environment (5); Loss (4), Body image (5); Juggling treatments and side effects (2); Independence (4); Death calculations (2); Cherishing the environment; Coveting time (2); Resolving spiritual issues (2) | 32 |  |
|  | WHOQOL-HIV^h^ [77-83]; 2004 | HRQoL | - Physical scale (16); Psychological scale (20); Level of dependence (16); Social relationships (16); Environment (32); Spirituality (16); General (4) | 12  (30 facets) |  |
|  | WHOQOL-HIV-BREF^i^ [84-95]; 2012 | HRQoL | - Physical health (4); Psychological health (5); Level of independence (4); Social relationship (4); Environmental health (8); Spirituality, religion, and personal beliefs (4); General items (2) | 31 |  |
|  | Instituto Superiore di Sanità Quality of Life [96]; 2006 | HRQoL | - Core evaluation form (37): satisfaction with quality of life (3), physical well-being (6), role well-being (2), depression and anxiety (2), energy and vitality (7), health distress (4), cognitive functioning (4), social functioning (4), and sexual life (5); Additional areas (25): social support (4), interaction with medical staff (9), treatment impact (3), body changes (4), life planning (2), and motherhood or fatherhood (3) | 62 |  |
|  | Symptom Quality of Life Adherence [97]; 2009 | HRQoL | - Two dimensions of HRQoL (physical and psychological) (12); Symptoms (short-term side effects) (13); Adherence VAS^j^ (1) | 26 |  |
|  | PROQOL-HIV^k^-43 [98-100]; 2012 | HRQoL | - Physical health symptoms^v^ (9); Treatment impact (10); Emotional distress (4); Health concerns (4); Body change (4); Intimate relationships (3); Social relationships (2); Stigma (2); Extra items (4); General health item (1) | 43 |  |
|  | PROQOL-HIV-38 [101]; 2016 | HRQoL | - Physical health symptoms (11); Concerns and mental distress (10); Social and intimate relationships (7); Treatment-related impact (10) | 38 |  |
|  | Poz Quality of Life [102]; 2018 | HRQoL | - Health concerns (3); Psychological scale (4); Social scale (3); Functional scale (3) | 13 |  |
| **Symptoms** | | | | |  |
|  | Riverside Symptom Checklist [103]; 1993 | HIV-related symptoms | - Physical symptom scale (19); Cognitive scale (6); Psychological scale (3) | 28 |  |
|  | HIV Symptom Index [104]; 194 | HIV-related symptoms | - Unidimensional scale (12): fatigue, fevers, paresthesia, imbalance, headache, memory loss, sadness, sleep disturbance (insomnia or daytime sleepiness), nausea, diarrhea, cough, and skin problems | 12 |  |
|  | HIV Assessment Tool [105]; 1994 | HIV-related symptoms | - EFA^l^ 1: HIV-related symptoms (23) and general well-being (11) - EFA 2: General well-being (12), general symptoms (14), and HIV-specific symptoms (8) | 34 in each EFA |  |
|  | SSC-HIV^m^ [106,107]; 1999 | HIV-related symptoms | - Malaise, weakness, or fatigue (6); Confusion or distress (6); Fever or chills (4); Gastrointestinal discomfort (4); Shortness of breath (3); Nausea or vomiting (3) | 26 |  |
|  | SSC-HIV-rev [108]; 2001 | HIV-related symptoms | - Eleven scales in part 1: numbness (4), fear (4), gastrointestinal upset (6), bruising or bleeding (4), fatigue (4), headache (4), sore throat (4), rectal itch (3), shortness of breath (3), fever (4), and body changes (5); One scale in part 3 concerned with gynecological symptoms for women (8) and items that did not load on factor scores (19) | 72 |  |
|  | HIV Cost and Services Utilization Study Symptom Measure [109]; 2000 | HIV-related symptoms | - Unidimensional scale (14) | 13 for male and 14 for female respondents |  |
|  | HIV Symptom Index or Symptoms Distress Module of the ACTG^n^ [110-112]; 2001 | HIV-related symptoms | - Unidimensional scale (20): 2 primary factors (2012), mental and physical symptom distress | 20 |  |
|  | HIV-Related Fatigue Scale [113-115]; 2002 | HIV-related fatigue | - Fatigue intensity (8); Overall function‐related fatigue (22): impact on activities of daily living (12), impact on socialization (6), and mental functioning (4); Triggers of fatigue (8); Alleviators of fatigue (4); Fatigue description (12) | 56 |  |
|  | HIV Disability Questionnaire [116-119]; 2013 | HIV-related disability | - Physical symptoms (20); Cognitive symptoms (3); Mental-emotional health symptoms (11); Difficulties carrying out day-to-day activities (9); Uncertainty and worrying about the future (14); Challenges to social inclusion (12) | 69 |  |
|  | Istituto Superiore di Sanità-HIV symptoms scale [120]; 2016 | HIV-related symptoms | - Pain or general discomfort (7); Depression or anxiety (4); Emotional reaction or psychological distress (5); Gastrointestinal discomfort (4); Sexual discomfort (2) | 22 |  |
| **Stigma** | | | | | |
|  | HSS^o^-40 [121, 122]; 2001 | HIV-related stigma | - Personalized stigma (16); Disclosure concerns (8); Negative self-image (8); Concerns with public attitudes (8) | 40 |  |
|  | HSS-32 [123, 124]; 2007 | HIV-related stigma | - Enacted stigma (11); Disclosure concerns (8); Negative self-image (7); Concern with public attitudes (6) | 32 |  |
|  | HSS-12 [125-127]; 2010 | HIV-related stigma | - Personalized stigma (3); Disclosure concerns (3); Concerns with public attitudes (3); Negative self-image (3) | 12 |  |
|  | HSS-39 [128]; 2014 | HIV-related stigma | - Personalized stigma (16); Disclosure concerns (8); Negative self-image (8); Concerns with public attitudes (7) | 39 |  |
|  | HSS-30 [129]; 2015 | HIV-related stigma | - Four factors: personalized stigma (8), disclosure concerns (9), negative self-image (8), and concern with public attitudes (5); Two factors: perceived external stigma (13) and internalized stigma (17) | 30 |  |
|  | HSS-10 [130]; 2020 | HIV-related stigma | - Personalized stigma (3); Disclosure (2); Negative self-image (3); Public attitudes (2) | 10 |  |
|  | HIV or AIDS stigma instrument-People living with AIDS [131,132]; 2007 | HIV-related stigma | - Verbal abuse (8); Negative self-perception (5); Social isolation (5); Fear of contagion (6); Health care neglect (7); Workplace stigma (2) |  |  |
|  | Internalized HIV Stigma Measure [133]; 2008 | Internalized HIV-related stigma | - Stereotypes (12); Disclosure concerns (5); Social relationships (7); Self-acceptance (4) | 28 |  |
|  | Internalized AIDS-Related Stigma Scale [134-136]; 2009 | Internalized HIV-related stigma | - Unidimensional scale (6) | 6 |  |
|  | Internalized Stigma in Those With HIV or AIDS [137]; 2011 | Internalized HIV-related stigma | - Unidimensional scale (10) | 10 |  |
|  | HIV- and Abuse-Related Shame Inventory [138]; 2012 | HIV- and abuse-related shame | - HIV-related shame (13); Impact of shame on behavior (8); Sexual abuse-related shame (10) | 31 |  |
|  | Self, Experienced, and Perceived HIV or AIDS Stigma Scales [139]; 2012 | HIV-related stigma | - Self-stigma (8); Experienced stigma (7); Perceived stigma (7) | 22 |  |
|  | HIV Stigma Mechanisms [140]; 2013 | HIV stigma mechanisms | - Internalized HIV stigma (6); Anticipated HIV stigma (9); Enacted HIV stigma (9) | 24 |  |
|  | HIV or AIDS Stigma Assessment for Latino Gay Men, Bisexual Men, and Transgender Women Living With HIV [141]; 2013 | HIV-related stigma | - Generalized enacted stigma (11); Romantic and sexual enacted stigma (2); Perceived stigma (15); Internalized stigma (8) | 36 |  |
|  | Van Rie HIV or AIDS-Related Stigma Scale-Revised for use in the United States [142]; 2015 | HIV-related stigma | - Perceived community stigma (8); Loss of social relationships (3); Managing HIV concealment (4) | 15 |  |
| **Psychological characteristics** | | | | | |
|  | The Mental Adjustment to HIV scale [143]; 1994 | Mental adjustment | - Helplessness or hopelessness (12); Fighting spirit (9); Denial-avoidance (5); Fatalism (6); Belief in influencing the course of the disease (8) | 40 |  |
|  | HIV or AIDS Stress Scale [144]; 2002 | Stress and coping | - Social stress (10); Instrumental stress (6); Emotional or existential stress (7) | 23 |  |
|  | Perceived Stress Scale Among People Living With HIV or AIDS [145]; 2008 | HIV-related stress | - Social or psychological problems (9); Sexual relationships (6); Functional problems (4); Social acceptance or rejection (4); Work-related issues (3); Family or offspring concerns (3); Accessibility to medical treatments (3); Concerns about medical treatment outcomes (3) | 35 |  |
|  | Screenphiv [146,147]; 2012 | Psychological issues related to HIV | - Emotional distress experience related to HIV (21); Personal growing attitude and positive coping (15); Cognitive change related HIV (11); Active coping focus on collective action and social support (6); Perceived rejection related to HIV and avoidance coping (5); Personal experience of rejection (5) | 63 |  |
|  | Impact on Self-Concept Scale [148]; 2013 | Impact of HIV on self-concept | - Self-loss (7); Self-growth (3) | 10 |  |
|  | Impact of HIV [149]; 2015 | Challenges of HIV survivorship | - Health awareness (6); Positive self-evaluation (3); Positive outlook (3); Value of relationships (6); Negative self-evaluation-outlook (12); Health Worry (5); Physical: body changes (3) | 38 |  |
|  | HIV Meaningfulness Scale [150]; 2015 | HIV meaningfulness | - Unidimensional scale (4) | 4 |  |
|  | People Living with HIV Resilience Scale [151]; 2019 | Resilience | - Unidimensional scale (10) | 10 |  |
| **Body and facial appearance** | | | | |  |
|  | Body Image in Patients With HIV or AIDS [152]; 2005 | Perceived body image | - Unidimensional scale (12) - 5 dimensions: comfort (2), competence (2), appearance (3), predictability (2), and existential self (3) | 12 |  |
|  | Owen Clinic Lipodystrophy Scale [153]; 2006 | Body change | - Fat loss (6); Fat accumulation (3); Retinoid changes (3) | 12 |  |
|  | ACTG-ABCD^p^ [154]; 2006 | Body change and distress | - Three parts: pertaining to speciﬁc signs of lipodystrophy (6), satisfaction with one’s body appearance (1), and psychosocial consequences of body changes (20) | 27 |  |
|  | ACTG-ABCD Short form [155]; 2014 | Body change and distress | - 10 items: negative affect about appearance (4), HIV health–related outcomes or stigma (2), and exercise or eating (2), ART^p^ (2); 18 items: negative affect about appearance (10), HIV health–related outcomes or stigma (4), exercise or eating (2), and ART (2) | 18 |  |
|  | Facial Appearance Inventory [156]; 2016 | Appearance | - Unidimensional scale (24) | 10 |  |
| **Treatment** | | | | |  |
|  | Medication Attribution Scale [157]; 1998 | Attributions about ART (its limitations on functioning, etc) | - Unidimensional scale (10) | 10 |  |
|  | HIVTSQ^q^ [158]; 2001 | Satisfaction with ART | - General satisfaction or clinical (5); Lifestyle or ease (4) | 9 |  |
|  | HIV Treatment Satisfaction Questionnaire status version [159]; 2006 | Satisfaction with ART | - General satisfaction or clinical (5); Lifestyle or ease (5) | 10 |  |
|  | Treatment-Related Empowerment Scale [160]; 2001 | Empowerment (involvement in treatment decision-making) | - Unidimensional scale (10) | 10 |  |
|  | Subcutaneous Injection Survey [161]; 2002 | Satisfaction with ART–subcutaneous injection | - Daily functioning (8); Ease of injection (4); Activities of daily living (3); | 15 |  |
|  | Quality of care through the patient’s eyes [162]; 2003 | Quality of care | - Generic items (13); HIV-specific items (14); Importance scores were linear transformed of standardized values (*z* scores) to values between 0 and 10. The performance score (P) represents the proportion of respondents who were not satisfied with the care received | 27 |  |
|  | Attitudes Toward HIV Health Care Provider scale [163]; 2004 | Attitudes toward health care providers | - Professionalism (8); Emotional support (11) | 19 |  |
|  | Antiretroviral General Adherence Scale [164]; 2006 | Ease and ability to adhere to ART | - Unidimensional scale (5) | 5 |  |
|  | Health Care Relationship Trust Scale [165]; 2006 | Trust toward health care providers | Interpersonal connection (5); Respectful communication (4); Professional partnering (6) | 15 |  |
|  | HIV Medication Readiness Scale [166]; 2007 | Readiness to adhere to ART | - Unidimensional scale (10) | 10 |  |
|  | SECope [167]; 2007 | Coping with the side effects of ART | - Positive emotion focused coping (5); Social support seeking (5); Nonadherence (4); Information seeking (3); Taking side-effect medications (3) | 20 |  |
|  | HIV Treatment Optimism Scale [168]; 2009 | Optimism about ART | - Susceptibility (10); Condom motivation (5); Severity (4) | 19 |  |
|  | HIV Medication Taking Self-Efficacy Scale [169]; 2010 | Self-efficacy to adhere to ART | - Self-efficacy beliefs (17); Outcome expectancy (9); | 26 |  |
|  | Brief Estimate of Health Knowledge and Action-HIV version [170]; 2010 | ART-related health literacy | - Knowledge (3); Action (5) | 8 |  |
|  | HIV Treatment Readiness Measure [171]; 2011 | Factors affecting the readiness for ART | - Disclosure (6); Psychosocial issues (9); Connection with care (6); HIV medication beliefs (12); Alcohol and drug use (5) | 38 |  |
|  | HIV Treatment Regimen Fatigue Scale [172]; 2015 | Regimen fatigue | - Treatment cynicism (15); Self-efficacy (7) | 22 |  |
|  | HIV Engagement in and Continuity of Care Scale [173]; 2017 | Engagement in care | - Access or getting care; Care by physician; Communication between health care professionals; Self-care | 26 |  |
| **Social support** | | | | | |
|  | Social Support Inventory [174]; 1999 | Received social support | - Nine subscales in total to assess the support received (for each of the following: have, want, and satisfaction): instrumental support, informational support, and emotional support (14, 17 [revised version, which is not tested]) | 14/17 |  |
|  | Unsupportive Social Interactions Inventory-HIV version [175]; 1999 | Unsupportive social interactions | - Insensitivity (6); Disconnecting (6); Forced optimism (6); Blaming (6) | 24 |  |
|  | Perceived Social Support for HIV [176]; 2014 | Perceived social support | - Belonging (2); Esteem (4); Self-development (6) | 12 |  |
| **Self-management and self-care** | | | | | |
|  | HIV Treatment Adherence Self-Efficacy Scale [177]; 2007 | Self-efficacy to adhere to HIV care | - Integration (9); Perseverance (3) | 12 |  |
|  | Perceived HIV Self-Management Scale [178]; 2011 | Self-efficacy for HIV self-management | - Unidimensional scale (8) | 8 |  |
|  | HIV Self-Management Scale (Women) [179]; 2012 | HIV Self-Management Scale (Women) | - Daily self-management health practices (12); Social support and HIV self-management (3); Chronicity of HIV self-management (5) | 20 |  |
|  | HIV Intention Measure [180]; 2012 | Intention to adhere to HIV care | - Intention (10); Knowledge (4) | 14 |  |
|  | HIV Exercise Stereotypes Scale [181]; 2016 | Stereotypes related to exercise in people living with HIV | - Stereotypes related to the benefits of exercise (5); Stereotypes related to the risks of exercise (4); Stereotypes related to the lack of capacity for exercise (5) | 14 |  |
|  | HIV Symptom Management Self-Efficacy for Women Scale [182]; 2011 | Self-efficacy for HIV symptom management | - Unidimensional scale (9) | 9 |  |

^a^Each version of a PROM is considered a separate PROM.

^b^This table expands on Table 1 in the main text; all cited references correspond to the main reference list of the article.

^c^HRQoL: health-related quality of life.

^d^MOS-HIV: Medical Outcomes Study-HIV Health Survey.

^e^HIV-PARSE: HIV Patient–Reported Status and Experience.

^f^HAT-QoL: HIV or AIDS-Targeted Quality of Life Instrument.

^g^MQoL: Multidimensional Quality of Life.

^h^WHOQOL-HIV: World Health Organization Quality of Life-HIV.

^i^WHOQOL-HIV-BREF: World Health Organization Quality of Life-HIV-Bref instrument.

^j^VAS: visual analog scale.

^k^PROQOL-HIV: Patient-Reported Outcome Quality of Life-HIV Questionnaire.

^l^SSC-HIV: Sign and Symptom Checklist for HIV.

^m^ACTG: Adult AIDS Clinical Trial Group.

^n^HSS: HIV Stigma Scale.

^o^ACTG-ABCD: Adult AIDS Clinical Trial Group’s Assessment of Body Change and Distress.

^p^ART: antiretroviral therapy.

^q^HIVTSQ: HIV Treatment Satisfaction Questionnaire.

## Table S4. Characteristics of the Included Records

| **Abbreviation of PROM** | **Author (Year)** | **Patient Characteristics** | | | **Disease Characteristics** | | | **Instrument Administration** | | |
| --- | --- | --- | --- | --- | --- | --- | --- | --- | --- | --- |
|  |  | **Total Number (N)** | **Age** | **Gender** | **Patient Description** | **Years Since Diagnosis** | **Severity of Disease** | **Recruitment Context** | **Country of Research** | **Effective Response Rate** |
| MOS-HIV | Carretero 1996^31^ | 100 | Median (IQR): 31.5 (28.5-35.5) | 18% woman  82% man | HIV+ with a history of IDU | Not mentioned | Asymptomatic: 27%;  symptomatic: 48%;  AIDS: 25%. | 69 were recruited from the HIV clinic at the Chelsea and Westminster Hospital in London, and 31 from the Griffin Project, a residential rehabilitation unit for HIV-seropositive IDU in the London area. | UK | 100/110=91% |
| MOS-HIV | Murri 1997^32^ | 213 | Median (IQR): 36 (34.7-36.8) | 66.6% man  33.4% woman | 18yrs+ HIV+ had a documented HIV infection without previous opportunistic infections or neoplasms | Days  Median (IQR): 1634 (1498-1770) | CD4+ T-cell count (cells/mm3) Median (IQR): 325 (291-358). | All consecutive people with HIV attending the outpatient clinic of a tertiary university hospital in Rome were asked to take part in the study. | Italy | 213/240=88.75% |
| MOS-HIV | Revicki 1998^33^ | Group 1: 1022  Group 2: 1231  Group 3: 134 | Mean (SD):  1: 38.7 (8.4);  2: 38.5 (7.8);  3: 36.7 | 1: 93% man  2: 94% man  3: 66% man | 18yrs+ HIV+ | Not mentioned | Group3: AIDS: 44%;  Asymptomatic: 25%;  Symptomatic: 31%. | 2 RCTs of ART for the treatment of HIV disease are used to develop and validate the physical and mental summary scores. A smaller longitudinal observational study provided additional data. | USA | Not mentioned |
| MOS-HIV | Badia 1999^34^ | 275 | Mean (SD): 35.9 (7.2) | 24.7% woman | 18-65 HIV+ | Months Mean (SD): 62.1 (47.4) | Asymptomatic: 38.5%; Symptomatic: 30.9%;  AIDS: 30.5%;  CD4+ T-cell count < 200: 26.9%; CD4+ T-cell count 200–499: 53.9%; CD4+ T-cell count≥500: 19.2%. | Randomly to a sample of 558 male and female HIV-infected patients recruited in 23 Spanish hospitals. | Spain | 275+283/558=100% |
| MOS-HIV | Holmes 1999^35^ | 215 | Mean (SD): 37.8 (8.6) | 80% man | 18yrs+ HIV+ | Months  Mean (SD): 58.7 (38.1) | Mean CD4+ T-cell count: 266 (241) cells/mm3. | Subjects were convenience-sampled from an urban, HIV specialty clinic; from an AIDS Clinical Trials Unit; and from outpatient clinics at a non-urban hospital located two hours from the urban sites. | USA | Not mentioned |
| MOS-HIV | Scott-Lennox 1999^36^ | 363 | Mean: 37.8 | 86.9% man | HIV+ | Not mentioned | CD4+ T-cell count: 228.20 cells/mm3; asymptomatic: 35.1%; symptomatic: 32.3%; AIDS: 32.6%. | Clinician investigators practicing in infectious disease clinics recruited HIV infected patients during routine clinic visits to volunteer for this non-randomized study. | England, France, Germany, Netherlands, and Italy | Not mentioned |
| MOS-HIV | Delate 2001^37^ | 242 | Mean (SD, Range): 39.8 (8.4, 21.8-69.8) | 85.5% man  14.5% woman | 18yrs+ HIV+ | Mean (SD, Range): 6.8 (4.2, 0.25-20.0) | CD4+ T-cell count (cells/mm 3): 407 (333, 1-1826); viral load (copies/ml): 67780 (131955, <50-609448). | Data were collected at an HIV specialty clinic. Clinic staff recruited potential participants as they signed in for routine clinical visits. | USA | 242/264=91.7% |
| MOS-HIV | Paton 2002^38^ | 163 | Mean: 38.1 | 96% man | 18yrs+ HIV+ | Not mentioned | CDC Stage A: 42%; CDC Stage B: 14%; CDC Stage C: 44%; Median(range) CD4+ T-cell count (cells/mm 3): 159(5-1379); Median(range) viral load (copies/ml): 27,500(0-1,350,000). | Consecutive male or female patients attending the outpatient clinics at the Communicable Disease Centre in Singapore, the national referral centre for HIV infection, were approached for study participation on selected days during the period of April to August 1998. | Singapore | 163/200=81.5% |
| MOS-HIV | Schifano 2003^39^ | 146 | Median: 35 | 73% man  27% woman | 21yrs+ HIV+ newly diagnosed with AIDS | Not mentioned | CD4+ T-cell count < 100 cells/ml: 75%; the lowest stage of AIDS severity: 43%. | Persons who participated in the randomized trial living in Rome and reported to the local AIDS registry (mandatory notiﬁcation). | Italy | 146/185=78.9% |
| MOS-HIV | Ichikawa 2004^40^ | 200 | Mean (SD): 33.5 (6.7) | 75% woman | 20yrs+ HIV+ | Mean (SD): 3.0 (2.2) | HIV/AIDS-related symptoms: 60%. | This study involved PWA in Chiang Mai province, northern Thailand, where 60 PWA self-help groups were identiﬁed in 1997. In 2000, we contacted ﬁve self-help groups in the municipal area and invited the PWA who attended their regular meetings to participate. | Thailand | Not mentioned |
| MOS-HIV | Lau 2006^41^ | 242 | Mean (SD): 38.38 (9.75) | 87.9% man | PLWHA receiving HAART | Not mentioned | Asymptomatic: 35%; symptomatic: 22%; AIDS: 43%; CD4+ T-cell count below 200 cells/mm3: 32%. | PLWHA who attended one of the two out-patient public clinics serving PLWHA in Hong Kong participated in this study. | China | 242/332=72.9% |
| MOS-HIV | Taylor 2009^42^ | 400 | Not mentioned | 58% woman  42% man | Adult HIV+ receiving treatment | Not mentioned | Not mentioned | 12 sites in Chipinge, Zimbabwe. Nine of 12 sites were traditional healer facilities and three were biomedical. | Zimbabwe | Not mentioned |
| MOS-HIV | Henderson 2010^43^ | 532 | Mean (SD): 42.40 (7.86) | 69.7% man  30.3% woman | 18yrs+ HIV+ on ART | Not mentioned | CD4+ T-cell count (cells/mm3): 455.94 (303.97); Undetectable HIV Viral Load: 58.9%. | Using cross-sectional data obtained between 1997 and 2007 at the baseline evaluation of a parent study designed to evaluate techniques to improve medication adherence in HIV infected adults on antiretroviral therapy. | USA | Not mentioned |
| MOS-HIV | Stasinopoulou 2010^44^ | 154 | Mean (SD): 42.6 (9.4) | 76.6% man  23.4% woman | 18yrs+ HIV+ known HIV infection for at least 3 months | Not mentioned | Asymptomatic: 50.6%; Symptomatic: 24.7%; AIDS: 24.7%. CD4+ T-cell Count (cells/mm3): 516.2 (269.4). HIV-1 RNA (median (IQR)): 49.5 (49–10,625). | Patients of the clinic of Infectious Diseases of a tertiary hospital in Athens, Greece. | Greece | 154/185=83.2% |
| MOS-HIV | Chariyalertsak 2011^45^ | 50 | Mean (SD, Range): 37.1 (7.0, 26-64) | 58% woman | 20yrs+ HIV+ current or past use of HAART | Not mentioned | CD4+ T-cell Count (cells/mm3): 189.25 (138.80). | Patients receiving ARV at two local HIV outpatient hospital clinics in northern Thailand. | Thailand | Not mentioned |
| MOS-HIV | Hsiung 2011^46^ | 619 | Mean (SD, Range): 35 (9.6, 16-78) | 95.5% man | HIV+ | Not mentioned | CD4+ T-cell Count (cells/mm3) <=200: 9.7%; receiving HAART: 73.2%. | Recruited from among HIV-infected outpatients at the National Taiwan University Hospital and Taipei City Hospital. The study sample comprised mainly of adult men. | China | 619/690=89.7% |
| MOS-HIV | Epino 2012^47^ | 610 | Mean (SD): 38 (10) | 62% woman | HIV+ adults | Not mentioned | HIV disease Stage 3 or 4: 49%; mean CD4+ T-cell count (cells/ml): 214 (92). | Initiated lifelong ART for the first time at one of nine health clinics in rural Rwanda. | Rwanda | Not mentioned |
| MOS-HIV | Huang 2012^48^ | 120 | Mean (SD, Range): 47.8 (7.6, 33–64) | 52.5% woman | 18yrs+ HIV+ have received ART for more than 30 days | Not mentioned | CD4+ T-cell count (cells/mm3): 434.4 (195.9); Time on ART, mean (SD) (months): 21.7 (1.7). | Recruited from the countryside of Linquan Country, Anhui Province. | China | 100% |
| MOS-HIV | Marzieh 2017^49^ | 100 | Mean (SD): 29 (1) | 100% man | 18yrs+ HIV+ at least primary education and the lack of mental and cognitive disorders | Mean (SD): 5(3) | CD4+ T-cell count (cells/mm3) <=200: 15%; 201-500: 36%; >500: 14%; Missing: 35%. | Recruited from behavioral disorder counseling centers affiliated to Shiraz University of Medical Sciences in Fars Province, south west of Iran. | Iran | Not mentioned |
| MOS-HIV | Liu 2018^50^ | 635 | Mean (SD, Range): 39.2 (7.6, 33-64) | 70.4% man  29.6% woman | 18yrs+ HIV+ | Not mentioned | CD4+ T-cell count (cells/mm3) <200: 14.8%, 200-500: 52.3%, >500: 32.9%.  21.3% Symptomatic. | Recruited from the local CDC and infectious diseases hospitals in three cities (Dalian, Ningbo, and Zhengzhou). | China | 635/646=98.3% |
| MOS-HIV | Shim 2018^51^ | 201 | Mean (SD): 48.49 (13.33) | 89.1% man  10.9% woman | Outpatients with HIV/AIDS | Mean (SD): 8.26 (5.97) | CD4+ T-cell counts (cells/mm3): 649.09 (306.96); Undetectable plasma viral load: 70.2%. | Recruited via consecutive sampling, multi-center survey involved four university hospitals in South Korea (two located in Seoul, the capital city, and the other two in the cities of Seongnam and Busan). | South Korea | Not mentioned |
| MOS-HIV-29 | Stangl 2012^52^ | 947 | 18-30: 16.8%;  31-40: 45.8%  41+: 37.4%. | 75.0% man  25.0% woman | 18yrs+ HIV+ confirmed HIV-1 infection | Not mentioned | CD4+ T-cell counts (cells/mm3)>100: 58.9%; Plasma HIV-1 (log10) copies/ml<5: 30.3%. | Enrolled from Tororo and Busia Districts in Eastern Uganda. | Uganda | Not mentioned |
| MOS-HIV-17 | McDonnell 2000^53^ | 287 | Mean (SD): 33.1 (6.27) | 100% woman | 18-44 female HIV+ not currently pregnant | Months  Mean (SD): 41.3 (29.9) | CD4+ T-cell counts >200 (cells/mm3): 75%. | HIV-infected women were enrolled from clinic and community sites in Baltimore City. | USA | 287/322=89.1% |
| HOPES | Schag 1992^54^ | 318 | Mean (Range): 38 (22-68) | 96% man  4% woman | HIV+ adults | Mean (Range): 3.82 (0.12-10.76) | Not mentioned | The Medical Centers serving HIV infected patients in the Los Angeles community including UCLA, community physicians, Veterans Affairs Medical Centers, and a County hospital: and additional data contributed from Johns Hopkins University Medical Center CMV Retinitis Clinic. | USA | 318/342=93.0% |
| HOPES | De Boer 1996^55^ | 106 | Mean (SD, Range): 38.0 (7.8, 22-65) | 93.4% man  6.6% woman | 18yrs+ HIV+ diagnosis of a symptomatic HIV infection or AIDS | Days mean (SD, Range): 127 (114, 1-631) | AIDS: 65.1%; Symptomatic HIV infection (ARC): 21.7%; AIDS/Kaposi Sarcoma: 13.2%; CD4+ T-cell counts (cells/mm3) mean (SD, Range): 212 (162, 10-950). | 45 patients were enrolled in a randomized clinical trial; the remaining 61 subjects were outpatients (not treated in a trial context) attending one of the five hospitals in Amsterdam providing AIDS care. | Netherlands | Not mentioned |
| HIV-QoL | Cleary 1993^56^ | 189 | Mean (SD): 36.9 (7.2) | 92.6% man  7.4% woman | CDC-defined AIDS | Not mentioned | CDC-defined AIDS | Patients had a visit to one of the sites (Clinic, HMO, Group practice) in Massachusetts. | USA | 189/293=64.5% |
| AIDS-HAQ | Lubeck 1997^57^ | HIV positive: 441; Symptomatic: 306;  AIDS: 336. | Mean (SD, Range):  HIV positive: 36.6 (0.9, 18-65); Symptomatic: 39.5 (0.5, 21-65); AIDS: 38.7 (0.3, 22-73). | Man:  HIV positive: 96.7%, Symptomatic: 98.3%, AIDS: 97.3%. | 18yrs+ HIV+ | Months Mean (SD): HIV positive: 30.6 (1.6); Symptomatic: 34.2 (2.1); AIDS: 33.5 (1.6). | CD4+ T-cell counts Mean (SD): HIV positive: 428 (16);  Symptomatic: 329 (13);  AIDS: 121 (16). | The study patients are recruited by participating physicians in six California locales: three in Northern California and three in Southern California. One site is a community referral clinic; the other sites are private practices. | USA | 1083/1865=58.07% |
| HIV-PARSE | Bozzette 1994^58^ | 1697 | Mean (SD): 36 (8) | 93% man | HIV+ | Not mentioned | CD4+ T-cell counts (cell/mm3): 131 (94). | Enrolled in one or more multicenter RCTs of chronic therapies for advanced HIV disease conducted under the sponsorship of the ACTG. | USA | 1697/1862=91.14% |
| HIV-PARSE-brief | Bozzette 1995^59^ | 1934 | Not mentioned | 7% woman | HIV+ | Not mentioned | CD4+ T-cells count (cell/mm3)  <50: 300 (22%);  50-200: 716 (52%);  >200: 366 (26%). | Enrolled in one or more clinical trials of therapies for advanced HIV disease. | USA | Not mentioned |
| HRQOL | Hays 1995^60^ | 205 | Not mentioned | 93% man  7% woman | 18yrs+ HIV+ | Months Mean: 33.7 | CD4+ T-cells count (cell/mm3): 144 (192).  Onset of symptoms: 24.2 months. | Collected at two public hospitals: a country-run facility (Harbor-UCLA Medical Center) and a Veterans Administration hospital in West Los Angeles. | USA | 205/314=65.29% |
| FAHI | Cella 1996^61^ | SMS: 110;  BESS: 71;  BSSS: 64. | Median (Range): SMS: 37.5 (18-61); BESS: 36 (20-57); BSSS: 39 (21-63) | Woman:  SMS: 0%;  BESS: 14%;  BSSS: 3%. | 18yrs+ HIV+ | Not mentioned | SMS CDC A1 or A2: 65%;  SMS CDC A3 or A4: 35%.  BESS CD4 T-cell count /mm3 >200: 33%;  BESS CD4+ T-cells count /mm3 <200: 67%;  BSSS CD4+ T-cells count /mm3 >200: 50%;  BSSS CD4+ T-cells count /mm3 <200: 50%. | SMS: 110 men at various stages of HIV disease progression enrolled in the PNI research programme;  BESS+BSSS: Data from the BIOQOL project. | USA | Not mentioned |
| FAHI | Peterman 1997^62^ | 361 | Median (Range): 39 (19–75) | 85% man  15% woman | 18yrs+ HIV+ | Months Median (Range): 40 (1–180) | CD4+ T-cells count (cell/mm3) >200: 29.8%;  CD4+ T-cells count (cell/mm3) ≤200: 70.2%. | 3 sources:  1. BIOQoL project; 2. N. L. McCain (unpublished) conducted a pilot study at Virginia Commonwealth University with 39 individuals; 3. A large community-based multi-centre trial. | USA | Not mentioned |
| FAHI | Viala-Danten 2010^63^ | Group1: 565;  Group2: 1096. | Mean (SD):  1: 44.7(8.1);  2: 46.1(7.7). | 1: 11％ woman  2: 10% woman | Treatment-experienced HIV-infected patients | 1: 13.4(4.2);  2: 14.4(4.0). | CD4+ T-cells count (cell/mm3)  1: 179.6(171.5); 2: 146.6(147.0). Mean plasma HIV RNA (log10 copies/mm3):  1: 4.6(0.8); 2: 4.8(0.6). | Patients were participants in two sets of clinical trials. | France | 563/565=99.6%  1091/1096=99.5% |
| FAHI | Byrne 2013^64^ | 170 | Mean (SD): 42.9 (6.9) | 61.2% man | 18yrs+ HIV+ with a current substance use problems diagnosis | Not mentioned | Not mentioned | Participants were recruited via announcements and referrals at two drop-in HIV/AIDS clinics in the greater Hartford, CT area. | USA | Not mentioned |
| FAHI | Nyongesa 2017^65^ | 103 | Mean (SD): 37.4 (7.78) | 94.2% woman | 18yrs+ HIV+ | Not mentioned | Not mentioned | Registered at the Comprehensive Care and Research Centre located within the Kilifi County Hospital at the Kenyan coast. | Kenya | Not mentioned |
| GHSA | Lenderking 1997^66^ | 1694 | Mean (SD, Range): 35.25 (8.69, 13-70) | 83.1% man | HIV1+ adult or adolescent | Not mentioned | Mean (SD, Range):  CD4+ T-cells count (cell/mm3): 354.2 (108.4, 125-1117). | The QoL sub-study was not a required part of ACTG Protocol 175 and some sites elected not to participate. At participating sites, every patient was offered participation in the QoL component of the study. | USA | 1694/1789=94.69% |
| HIV-QL31 | Leplège 1997^67^ | 102 | Mean (SD, Range): 38.3 (9.0, 24-73) | 74.5% man  25.5% woman | HIV+ half of the group had to have no sign of cyto megalo virus infection | Not mentioned | Not mentioned | Subjects recruited in various medical and nonmedical settings in two French cities (Paris and Nancy). | France | Not mentioned |
| HAT-QoL-42 | Holmes 1997^68^ | 106 | Mean (SD): 37.8 (8.9) | 74% man | 18yrs+ HIV+ | Seropositivity time> 24 months: 79%. | Mean CD4+ T-cells count (cells/mm3): 358.4 (293.3); HIV-related disability: 27%. | Subjects for the second study were convenience-sampled from an urban, HIV specialty clinic, a medium-sized, rural hospital's outpatient clinics, an AIDS Clinical Trials Unit, and an urban, hospital-affiliated outpatient medical clinic. | USA | Not mentioned |
| HAT-QoL-42 | Holmes 1998^69^ | 201 | Mean (SD): 37.5 (8.1) | 78% man | 18yrs+ HIV+ | Months Mean (SD): 61.2 (38.4) | Mean CD4+ T-cells count (cells/mm3): 249.1 (262.2); AIDS: 62%. | Subjects for the second study were convenience-sampled from an urban, HIV specialty clinic, a medium-sized, rural hospital's outpatient clinics, an AIDS Clinical Trials Unit, and an urban, hospital-affiliated outpatient medical clinic. | USA | Not mentioned |
| HAT-QoL-30 | Holmes 1999^35^ | 215 | Mean (SD): 37.8 (8.6) | 80% man | 18yrs+ HIV+ | Months Mean (SD): 58.7 (38.1) | Mean CD4+ T-cells count (cells/mm3): 266 (241). | Subjects were convenience-sampled from an urban, HIV specialty clinic; from an AIDS Clinical Trials Unit; and from outpatient clinics at a non-urban hospital located two hours from the urban sites. | USA | Not mentioned |
| HAT-QoL-34 | Holmes 2008^70^ | 153 | Mean (SD): 37.9 (8.5) | 71% man | Patients with treatment experience | Months Mean (SD): 69.8 (49.0) | Mean CD4+ T-cells count (cells/mm3): 382 (304). | Participants were recruited from an HIV-specialty clinic or from outpatient clinics at a non-urban hospital located two hours from the urban site. | USA | Not mentioned |
| HAT-QoL-34 | Taylor 2009^42^ | 400 | Not mentioned | 58% woman  42% man | Adult HIV+ receiving treatment | Not mentioned | Not mentioned | 12 sites in Chipinge, Zimbabwe. Nine of 12 sites were traditional healer facilities and three were biomedical. | Zimbabwe | Not mentioned |
| HAT-QoL-34 | Mafirakureva 2016^71^ | 257 | Mean (SD): 39.7 (8.9) | 72% woman | 18-60 years HIV+ | Median ART: 24 months. | Median CD4+ T-cells count (cells/mm3): 343 (8–1431) cells/mm3. | Chitungwiza Central Hospital, HIV+ patient receiving ART were randomly selected and invited to participate during their clinic visits. | Zimbabwe | 257/350=73.4% |
| MQoL-HIV | Smith 1997^72^ | Group 1: 121;  Group 2: 95. | Not mentioned | 1: 29.8% woman;  2: 100% man. | HIV+ adults | Not mentioned | CD4+ T-cells count (cells/mm3): 1: < 200: 38%;  2: < 200: 33.3%. | Recruited from two health centres in the Boston metropolitan area. | USA | Not mentioned |
| MQoL-HIV | Badia 1999^34^ | 283 | Mean (SD): 36.4 (7.5) | 25.9% woman | 18-65 HIV+ | Months Mean (SD): 63.25 (46.5) | Asymptomatic: 34.6%; Symptomatic: 33.2%; AIDS: 32.2%; CD4+ T-cells count (cells/mm3): < 200: 35.5%; 200–499: 43.6%; ≥500: 20.9%. | Randomly to a sample of 558 male and female HIV-infected patients recruited in 23 Spanish hospitals. | Spain | 275+283/558=100% |
| MQoL-HIV | Kemmler 2003^73^ | 207 | Mean (SD): 37.8 (9.5) | 57.0% man  43.0% woman | 18yrs+ HIV+ | Median: 66 months | CDC stage A: 27.0%; CDC stage B: 31.9%; CDC stage C: 41.1%. Viral load (log10 copies/mm3) <4.3: 77.2%; Current HIV therapy: 72.9%. CD4+ T-cells count (cells/mm3): >500: 36.9%; <200: 15.5%. | Out-patients contacting the HIV/ AIDS Unit at one of the following two institutions: Innsbruck University Clinics and Otto Wagner Hospital, Vienna. | Germany | 207/227=91.2% |
| MQoL-HIV | Remple 2004^74^ | 85 | Mean (SD, Range): 36.5 (9.5, 19-60) | 100% woman | HIV-infected women | Mean (SD, Range): 5.4 (4.0, 0-17) | Viral Load Undetectable: 41%; Median (IQR) plasma HIV RNA (copies/ mm3): 22,500 (3685, 129,000); CD4+ T-cells count (cells/mm3): 415 (218). | At the Oak Tree Clinic in the Women’s Health Centre of British Columbia in Vancouver. | Canada | Not mentioned |
| MQoL-HIV | Watanabe 2004^75^ | 375 | Mean (SD, Range): 36.5 (10.3, 20-74) | 8% woman | 20yrs+ HIV+ | Not mentioned | CD4+ T-cells count (cells/mm3): 409 (227, 2-1224); Mean plasma HIV RNA (copies/mm3): 11334 (52968, <400-680000) | Patients with HIV/AIDS from the AIDS Clinical Centre, International Medical Centre of Japan and eight other regional HIV treatment hospitals in Japan were recruited. | Japan | 375/423=88.7% |
| LWHIVS | Holzemer 1998^76^ | 187 | Mean (SD, Range): 40.64 (8.82, 23-75) | 65.8% man;  34.2% woman; 3.2% nonbinary | 23-75 HIV+ | Mean (SD, Range): 5.27 (3.76, 0-15) | CD4+ T-cells count (cells/mm3): 229 (204, 0-905). | Several community-based organizations and outpatient clinics related to their HIV care. | USA | 187/218=85.78% |
| WHOQOL-HIV | Starace 2002^77^ | 151 | Mean (SD): 35.6 (7.3) | 70% man | HIV+ adults | Not mentioned | CD4+ T-cells count (cells/mm3): 350.4 (226.9); HIV viral load (log10 copies/mm3): 9.7 (1.6); HAART: 71.1%. | The survey has been carried out in the largest infectious disease hospital in Italy (Cotugno Hospital, Naples). | Italy | 151/160=94.4% |
| WHOQOL-HIV | WHOQOL HIV Group 2004^78^ | 1334 | Mean (SD): 33.9 (9.36) | 66.6% man | HIV+ adults | Not mentioned | Asymptomatic: 43.9%; symptomatic: 34.5%; AIDS-converted: 21.6%. | Participants were recruited from departments or faculties where they sought help. convenience recruited from seven centres (Australia; Brazil; Italy; Thailand; Ukraine; and two sites in India: Bangalore; and New Delhi). | Australia, Brazil, India, Thailand, Italy, and Ukraine | Not mentioned |
| WHOQOL-HIV | Zimpel 2007^79^ | 308 | Mean (SD): 34.6 (8.1) | 60.4% man | 18yrs+ HIV+ with pre-documented HIV infection | Mean: 4.1 | Asymptomatic: 42.5%; Symptomatic: 29.5%; AIDS: 27.9%; CD4+ T-cells count (cells/mm3): 431.8(263.3); 71.1% used two or more ART drugs. | At the two HIV/AIDS referral centres, the Rio Grande do Sul Sanitary Dermatology outpatient clinic and the outpatient and inpatient services for HIV/AIDS of Hospital de Clı´nicas de Porto Alegre. | Brazil | Not mentioned |
| WHOQOL-HIV | Canavarro 2011^80^ | 200 | Mean (SD, Range): 39.23 (9.21, 18-72) | 60% man;  39.5% woman;  0.5% nonbinary | 18yrs+ HIV+ | Mean (SD, Range): 8.69 (4.81, 1-21) | Asymptomatic: 43.7%; Symptomatic: 18.6%; AIDS: 19.6%; Unknown: 18.1%. | Contacted the Department of Infectious Diseases of the University of Coimbra Hospitals. | Portugal | 200/210=95.2% |
| WHOQOL-HIV | Mweemba 2011^81^ | 160 | Mean (SD): 36.52 (8.98) | 50% woman | 18yrs+ HIV+ | Not mentioned | Mean CD4+ T-cells count (cells/mm3): 358.35;  HAART: 86.9%;  Asymptomatic: 45%;  AIDS: 25%. | The study was conducted in two urban and two rural sites in Zambia. Clinic managers recruited subjects from antiretroviral clinics. | Zambia | 160/166=96.38% |
| WHOQOL-HIV | Olsen 2013^82^ | 20 | Range: 20-49 | 55% woman | 20-49 yrs HIV+ | Mean (Range): 3 (0.5-9) | All had initiated ART treatment. | Included initial observations at the ART clinic and home visits of patients as well as informal interviews with peer educators at Jimma University Specialized Hospital and the managers of three local HIV organizations. | Ethiopia | Not mentioned |
| WHOQOL-HIV | Reychler 2013^83^ | 50 | Mean (SD, Range): 41.2 (10.9, 20-66) | 64% man  36% woman | 18yrs+ HIV+ infected with HIV for at least 6 months | Not mentioned | CD4+ T-cells count (cells/mm3): 605.4; Undetectable viral load 84%; HAART: 90%; Asymptomatic: 78%; Symptomatic: 6%; AIDS: 16%. | HIV-infected patients regularly attending the outpatient infectious disease clinic of Cliniques universitaires Saint-Luc were recruited on a voluntary basis and without financial compensation for this study. | Belgium | 50/64=78.1% |
| WHOQOL-HIV-BREF | Saddki 2009^84^ | 157 | Mean (SD, Range): 35.7 (7.50, 18-67) | 59.9% man  40.1% woman | Malaysian 18yrs+ HIV+ ART at least six months | Not mentioned | Asymptomatic: 59.9%; Symptomatic: 40.1%. | PLWHA who came for treatment at the Infectious Disease Unit of Hospital Raja Perempuan Zainab II, Kota Bharu, Kelantan. | Malaysia | 157/160=98.1% |
| WHOQOL-HIV-BREF | Hsiung 2011^85^ | 680 | Mean (SD): 36.3 (10.1) | 95% man | 18yrs+ HIV+ | Not mentioned | CD4+ T-cells count (cells/mm3): 450.04 (219.2); viral load<50 (copies/ml): 57.1%. | Recruited from the National Taiwan University Hospital and the Taipei City Hospital. | China | 680/690=98.6% |
| WHOQOL-HIV-BREF | O'Connell 2012^86^ | 1923 | Mean (SD): 33.4 (9.8) | 65.7% man  34.3% woman | HIV+ | Not mentioned | Asymptomatic: 41.1%; Symptomatic: 33.1%;  AIDS: 24.8%. | The pilot study contained data from six centres: Australia, Brazil, South and North India: Bangalore and New Delhi respectively, Thailand and Zimbabwe. The field trial was conducted in seven centres and contained a second wave of data from five pilot centres (excluding Zimbabwe), and two new centres; Italy and Ukraine. | Australia, Brazil, India, Thailand, Zimbabwe, Italy, and Ukraine | 1923/2225=86.4% |
| WHOQOL-HIV-BREF | Tran 2012^87^ | 1016 | Mean (SD): 35.4 (7.0) | 63.8% man  36.2% woman | HIV+ adults | Not mentioned | AIDS: 37.6%; Symptomatic: 50.0%; Asymptomatic: 12.4%; taking ART: 88.8%; CD4+ T-cells count (cells/mm3)≤500: 86.4%. | Cross-sectional study in 3 epicenters of Vietnam, namely the 2012 Vietnam HIV Services Users Survey. Three metropolitan areas, including Ha Noi, Hai Phong, and Ho Chi Minh City, were selected to represent different geographical areas of the largest HIV epidemics in Vietnam. | Vietnam | Not mentioned |
| WHOQOL-HIV-BREF | Canavarro 2012^88^ | 1196 | Mean (SD, Range): 40.73 (9.69, 18-81) | 67.6% man  32.4% woman | 18yrs+ HIV+ | Mean (SD): 8.06 (4.81) | CD4+ T-cells count (cells/mm3) <200 cells/mm3: 23.5%; 201-499: 42%; >500: 34.6%. | The study sample comprised HIV-infected patients contacting the main departments of infectious diseases in Portuguese hospitals (a total of 10 institutions), recruited by convenience sampling. | Portugal | 1196/1251=95.6% |
| WHOQOL-HIV-BREF | Pereira 2014^89^ | 185 | Mean (SD, Range): 57.84 (6.79; 50–81) | 64.9% man  35.1% woman | 50yrs+ HIV+ | Mean (SD, Range): 6.34 (5.20; 0-24) | Median (SD, range):  CD4+ T-cells count (cells/ mm3): 433.06 (275.65; 5–1234) | The main departments of infectious diseases of 10 Portuguese hospitals. | Portugal | Not mentioned |
| WHOQOL-HIV-BREF | Meemon, 2016^90^ | 329 | Mean (SD): 41.95 (7.82) | 55.6% woman | HIV+ adults | Mean (SD): 10.40 (6.04) | CD4+ T-cells count (cells/mm3) = 489.51 (249.35);  98.2% Taking ART. | Recruited by convenience from all seven hospitals and consisted of adult HIV-infected patients who visited the HIV outpatient unit during the hospitals’ active HIV clinic days in Phayao Province, Thailand. | Thailand | 329/518=63.5% |
| WHOQOL-HIV-BREF | Salehi 2016^91^ | 61 | Mean (SD): 38.06 (9.32) | 72.1% man  27.9% woman | 18yrs+ HIV+ | Not mentioned | All in the AIDS stage of their disease and used HAART | Regularly attended the outpatient infectious disease clinic consultation centre for patients with behavioral disorders in the North East of the Islamic Republic of Iran. | Iran | Not mentioned |
| WHOQOL-HIV-BREF | Tesfaye 2016^92^ | 348 | Mean (SD): 32.9 (8.8) | 66.7% woman | HIV+ adults | Not mentioned | Not mentioned | Jimma University Specialized Hospital, located in Jimma City, and two primary care centres (one in Jimma City, the other in Agaro town). | Ethiopia | Not mentioned |
| WHOQOL-HIV-BREF | Zhu 2017^93^ | 1100 | Mean (SD): 39.62 (12.73) | 87.7% man  12.3% woman | 18yrs+ HIV+ without cognitive impairment | Mean (SD): 3.91(2.85) | Symptomatic: 37.2%, Asymptomatic: 62.8%; CD4+ T-cells count (cells/mm3)< 200: 9.0%; 200–499: 58.4%; ≥500: 32.6%. | Recruited from local Centers for Disease Control and Prevention branches and infectious disease hospitals from five cities (Shenyang, Dalian, Dandong, Zhengzhou and Ningbo). | China | 1100/1201=91.6% |
| WHOQOL-HIV-BREF | Fuster-RuizdeApodaca 2019^94^ | 1462 | Mean (SD): 45.0 (10.2) | 79.3% man  19.3% woman  1.4% nonbinary | HIV+ adults | Mean (SD): 13.8 (9.6) | Undetectable viral load: 90.4%; CD4+ T-cells count (cells/mm3)>400: 68.1%. | 33 service delivery points (hospitals and non-governmental organizations). | Spain | Rate of refusal to participate in the study average 7% (0-18%) |
| WHOQOL-HIV-BREF | Barger 2020^95^ | 586 | Median (IQR): 55.8 (48.9–62.8) | 73.2% man | Adults (≥ 18 years old) with a confirmed HIV-1 diagnosis | Median (IQR): 20.1 (11.8-27.7) | CD4+ T-cells count (cells/mm3)≥500: 72.0%; viral load (copies/ml)<50: 92.7%. | 13 public hospitals in the Nouvelle Aquitaine region of south-western France of the ANRS CO3 Aquitaine cohort. | France | 586/587=99.8% |
| ISSQoL | Bucciardini 2006^96^ | 322 | Mean (SD, Range): 40.0 (7.3, 18–68) | 60.8% man  35.5% woman  3.6% missing | 18yrs+ HIV+ | Mean (SD, Range): 10.4 (5.4, 0.5-24) | Asymptomatic: 30.7%; Symptomatic: 43.1%; AIDS: 23.8%; missing: 2.4%. | Persons from clinics and CBOs, equally distributed over 15 out of 21 regions of Italy were enrolled. | Italy | 322/332=97.0% |
| HIV-SQUAD | Spire 2009^97^ | 590 | Mean: 42.1 | 67.9% man | ARV-naive or as ARV-experienced patients | Diagnosed and treated with ARV treatment for 8.7 years. | CDC stage A: 46.6%; CD4+ T-cells count (cells/mm3): 401.5; viral load (log10 copies/ml): 3.50. | HIV patients treated with a combination of antiretroviral drugs were enrolled in an observational, longitudinal study in France. | France | 599/607=98.7% |
| PROQOL-HIV-43 | Duracinsky 2012 (1)^98^ | 152 | Median: 40 | 56% man | PLWHA | Median: 7 | Median CD4+ T-cells count (cells/mm3): 378; Treated: 84%; CDC stage A: 31%; CDC stage B: 18%; CDC stage C: 51%. | Recruited from 11 centers across 9 countries (Australia, Brazil, Cambodia, China, France,  India, Senegal, Thailand, and USA). | 9 countries | Not mentioned |
| PROQOL-HIV-43 | Duracinsky 2012 (2)^99^ | 791 | Median (IQR): 41 (34-48) | 36% woman | 18yrs+ HIV-seropositive outpatients receiving routine HIV clinical care | Diagnosis years from 2008 Median (IQR): 5 (2-10) | Median (IQR) CD4+ T-cells count (cells/mm3): 404 (246-552). | HIV patients were recruited from 9 centers in 8 countries (Australia, Brazil, Cambodia, China, France, Senegal, Thailand, and USA). | 8 countries | 791/826=98.8% |
| PROQOL-HIV-43 | Duracinsky 2014^100^ | 79 | Mean (SD): 46.7 (10.9) | 66% man  34% woman | 18yrs+ HIV-seropositive outpatients receiving routine HIV clinical care | Mean (SD, IQR): 15 (8, 8-23) | CD4+ T-cells count (cell/mm3): 432 (338);  undetectable viral load (n=21): 9 (43%). | The Kremlin Bicêtre hospital (Assistance Publique-Hôpitaux de Paris) and the institutional apartments for people living with HIV, Service ACT du Val de Marne, Fondation Maison des champs. | France | 79/80=98.75% |
| PROQOL-HIV-38 | Lalanne 2016^101^ | 2537 | Mean (SD): 47.4 (10.8) | 70.6% man  29.4% woman | 18yrs+ HIV+ diagnosed at least 6 months before participating in the study | Median (Range): 18 (5-33) | 93.6% Antiretroviral treatment; CD4+ T-cells count (cell/mm3): 392.1 (273.8); Undetectable viral load: 73.6%. | During the VESPA2 national survey (2011-2012), A total of 68 primary sampling units were considered in the final database. | France | 2537/3022=83.95% |
| PozQol | Brown 2018^102^ | 465 | Mean (SD): 46.26 (11.98) | 81.3% man  3% woman  15.7% missing or nonbinary | 18yrs+ HIV+ | Year first tested positive for HIV  1980-1995: 105  1996-2009: 178  2010-2017: 172 | undetectable viral load: 432 (92.9). | Advertised through PLHIV community organizations and online platforms including Facebook, the Facebook page of the Institute of Many, and Grindr. Additionally, hard copies of the survey were distributed through PLHIV community organizations in Victoria. | Australia | Not mentioned |
| RSC | Burgess1993^103^ | A: 71  S: 14  AIDS: 18 | Mean (SD): A: 35 (8.7); S: 33 (5.4); AIDS: 35 (5.5). | 100% gay men | HIV+ gay or bisexual men | Not mentioned | CD4+ T-cells count (cells/mm3): A: 445 (10-1194)  S: 215 (14-342); AIDS: 75 (18-228). | All subjects were gay or bisexual men who had attended one of 3 HIV dedicated outpatient clinics. | UK | Not mentioned |
| HSI | Whalen 1994^104^ | 148 | Not mentioned | 87% man | HIV+ outpatients | Not mentioned | AIDS: 32%; AIDS-Related Complex (ARC): 30%; Pre-ARC: 38%. | The study group consisted of a random sample of HIV-infected outpatients who attended an ambulatory clinic that specializes in their care at University Hospitals of Cleveland. | USA | 148/197=75.1% |
| HAT | Nokes 1994^105^ | Healthy: 53;  HIV+: 60;  AIDS: 43. | Range: Healthy: 19-60; HIV+: 28-67; AIDS: 27-68. | Healthy: 13.2% man;  HIV+: 98% man;  AIDS: man 100%. | 18yrs+ HIV+ | HIV+: diagnosed with HIV disease was 35.5 (range 2-125) months; AIDS: diagnosed with CDC-defined AIDS was 25 (range 1-79) months | Hospitalized or ambulatory HN positive (n=60); Hospitalized or outpatients with CDC-defined AIDS (n=43); and healthy college students (n=53). | The group with HIV disease or AIDS was receiving health care at a veteran hospital in New York City; Healthy subjects were students at Hunter College who indicated on the subject information section of the questionnaire that they were HIV negative. | USA | Not mentioned |
| SSC-HIV | Holzemer 1999^107^ | 686 | Mean (Range): 39.4 (20-64) | 22.8% woman | HIV+ | Mean (Range): 7.41 (1-25) | CD4+ T-cells count (cells/mm3): 323 (1-1500); AIDS: 61.3%. | Seven cities across the United States: Birmingham, Boston, Honolulu, New York, San Francisco Bay area, Seattle and Tampa. Specific sites within these cities varied and included university-based AIDS clinics, private practices, public and for-profit hospitals, residential and day care facilities, community-based organizations and home care. | USA | Not mentioned |
| SSC-HIV | Sousa 2006^106^ | 917 | Mean (SD): 39.35 (8.13) | 100% man | Diagnosed with AIDS | Months Mean (SD): 50.63 (4.22) | Not mentioned | The data for this analysis included response sets to surveys that were collected as part of the AIDS Time-Oriented Health Outcome Study databank. The ATHOS is a longitudinal database of persons with HIV-associated illness. | USA | Not mentioned |
| SSC-HIV-rev | Holzemer 2001^108^ | 372 | Mean (SD): 40.0 (8.3) | 31.7% woman | HIV+ adults | Year of HIV diagnosis:  1975-1990: 24%; 1991-2000: 72%. | AIDS diagnosis: 54.0%; No AIDS diagnosis: 37.6%; No Hospitalized with AIDS: 76.1%. | The setting for data collection was an HIV/AIDS outpatient clinic in Harris County, Texas, which provides primary care for people living with HIV/AIDS. | USA | Not mentioned |
| HCSUS Symptom Measure | Mathews 2000^109^ | 3072 | Not mentioned | Not mentioned | 18yrs+ HIV+ receiving medical care | Not mentioned | Not mentioned | Patients selected from among all patients receiving care from 145 providers in 28 metropolitan areas and 51 providers in 25 rural areas. | USA | 3072/4042=76% |
| HIV-SI/SDM | Justice 2001^110^ | 113 | Median: 44 | 5% woman  95% man | HIV+ | Not mentioned | Median CD4+ T-cells count (cells/mm3): 365; Median viral load (copies/mm3): 423. | Sample of all active patients at the Cleveland Veterans Affairs Medical Center HIV clinic. | USA | 113/115=98.3% |
| HIV-SI/SDM | Regnault 2009^111^ | 759 | Mean (SD): 37.0 (9.5) | 71% man | HIV-1-infected treatment-naive patients | Not mentioned | HIV RNA concentration (in copies/mL)≥100,000: 47%; CD4+ T-cells count (cells/mm3): 268.5 (140.3). | The trial was conducted in 12 countries: Argentina, Australia, Belgium, Canada, Italy, Mexico, Poland, the United Kingdom, South Africa, Switzerland, the Netherlands, and the United States. | 12 countries | 759/917=82.8% |
| HIV-SI/SDM | Marc 2012^112^ | 5521 | Mean (SD, Range): 43.0 (8.7, 18-91) | 88.6% man  11.4% woman | 18yrs+ HIV+ | Not mentioned | Median (SD) CD4+ T-cells count (cell/mm3): 429 (275.6); Median (SD) viral load (log10 copies/ml): 2.6 (1.2). | A secondary data analysis of a cross-sectional subset of the Collaborations HIV Outcomes Research US Cohort, which is a population-based observational study designed to follow the clinical and epidemiological outcomes of adults with HIV infection in the US. | USA | Not mentioned |
| HRFS | Barroso 2002^113^ | 54 | Mean (SD): 41 (6.3) | 78% man | HIV+ adults | Not mentioned | Not mentioned | Research assistant visited prospective participants in their homes to ascertain their agreement to participate. | USA | Not mentioned |
| HRFS | Pence 2008^114^ | 128 | Median (IQR): 44 (38-48) | 66% man | 21yrs+ HIV+ | Median (IQR):  10 (6-15) | 82% Receiving ART at baseline | Participants were recruited via flyers advertising the study at HIV/AIDS treatment centers and service organizations in a southern US state. | USA | Not mentioned |
| HRFS | Li 2016^115^ | 142 | Mean (SD, Range): 33.1 (9.4, 22-73) | 97,2% man  2.8% woman | 18yrs+ HIV+ | Not mentioned | 76,1% Receiving HAART | Recruited from the Infectious Diseases Outpatient Clinic and the HIV/AIDS Case Management Program of a medical centre in central Taiwan. | China | 142/192=74.0% |
| HDQ | O'Brien 2014^116^ | 361 | Median (IQR): 46 (40–52) | 80% man  18% woman  2% nonbinary | 18yrs+ HIV+ | Median (IQR)  16 (9–23); | Undetectable viral load: 76%;  Taking ART: 83%;  CD4+ T-cells count (cell/mm3) <200: 46%. | Recruited adults living with HIV by posting recruitment posters and brochures in hospital clinics, AIDS service organizations and a specialty hospital in southern Ontario. | Canada | Not mentioned |
| HDQ | O'Brien 2015^117^ | 235: 139 Canadian, 96 Irish | Median (IQR):  Canada: 48 (44-45);  Ireland: 41(34-48) | Canada: 17% woman;  Ireland: 24% woman. | 18yrs+ HIV+ self-identified as having experienced an episode of illness attributed to HIV | Median (IQR):  Canada: 16 (11–25); Ireland: 12 (6–17). | Undetectable viral load: Canada: 89%, Ireland: 85%;  Taking ART: Canada: 91%, Ireland: 88%. | The majority of Irish participants (89; 93 %) were recruited from St. James’s Hospital GUIDE Clinic in Dublin, whereas the majority of Canadian participants (129; 91 %) were recruited from HIV community-based service organizations in southern Ontario. | Canada and Ireland | 235/239=98.33% |
| HDQ | Brown 2019^118^ | 243 | Median (IQR) (Range): 40 (33-48) (22-67) | 100% man | 18yrs+ HIV+ | Median year of diagnosis of 2012, 96% diagnosed 1996 or after. | Undetectable viral load (<40 copies/ml) (n=193): 81.8%; Median (IQR) CD4+ T-cells count (cells/mm3): 676 (508-875). | An outpatient HIV clinic in central London, UK. | UK | 243/244=99.6% |
| HDQ | O’Brien 2019^119^ | 128 | Median (IQR)：51 (43-58) (range: 22-79) | 68.3% man  30.1% woman  1.6% nonbinary | Adults living with HIV in US | Median (IQR) (range): 16 (8-21) (<1-36) | Undetectable viral load (<50 copies/ml): 93.7%; 96.0% Receiving HAART. | Recruited adults living with HIV from Garden State Infectious Disease Associates in Voorhees, New Jersey. | USA | 128/132=97.0% |
| ISS-HIV-SS | Bucciardini 2016^120^ | 161 | Mean (SD, Range): 49.0 (9.8, 25–77) | 24.2% man  75.8% woman | 18yrs+ HIV+ | Mean (SD, Range): 13.4 (9.2, 0–30) | CD4+ T-cells count (cells/mm3): 652 (312, 24–1686); Viral load (log10 copies/ml): 1.76(0.6, 1–6). | From the two Italian clinical centers (Azienda Ospedaliera Universitaria Ospedali Riuniti, Ancona; Ospedale S. Maria della Misericordia, Perugia). | Italy | Not mentioned |
| HSS-40 | Berger 2001^121^ | 318 | Mean (SD, Range): 37.0 (7.7, 19-82) | 80.6% man  19.4% woman | 18yrs+ HIV+ | Mean (SD, Range): 4.2 (3.1, 0-11) | AIDS: 54.4%. | More than 60 organizations in eight states (Illinois, Missouri, Wisconsin, Kansas, Michigan, Arizona, New York, and Texas), such as HIV clinics, physicians' offices, HIV support groups, and HIV/AIDS service organizations, agreed to help distribute the questionnaires. The clientele of these organizations ranged from individuals who were indigent or uninsured to those who were employed and had health insurance. | USA | Not mentioned |
| HSS-40 | Rao 2008^122^ | 541 | Not mentioned | 67% man  33% woman | Not mentioned | Not mentioned | Not mentioned | PLWHA recruited from AIDS Service Organizations and Ryan White Title II/III clinics in South Carolina and from Department of Public Health clinics in Iowa. | USA | Not mentioned |
| HSS-32 | Bunn 2007^123^ | 157 | Mean (SD, Range): 43.2 (9.0, 19-64) | 71% man | 18yrs+ HIV+ | Mean (SD): 11.8 (6.9) | Not mentioned | The sample consisted of 157 individuals living with HIV/AIDS in Vermont and northern New England, who were recruited from five infectious disease clinics, six AIDS service organizations, advertisements in local media, and by word of mouth. | USA | Not mentioned |
| HSS-32 | Reinius 2018^124^ | 1036  South India: 250;  Sweden: 188;  US: 598. | Mean (SD, Range): 41 (11, 18–83) | 40% woman | 18yrs+ HIV+ | Not mentioned | Not mentioned | Swedish cohort: Karolinska University hospital in Stockholm; South Indian cohort: networks caring for men and women living with HIV in the state of Tamil Nadu; US cohort: Ryan White II/III clinics and a community-based organization providing medical case management in South Carolina (n = 210), Iowa Department of Public Health Title II case-managed clients in Iowa (n = 331), and a private University Hospital HIV Clinic in Chicago (n = 62). | South India, Sweden, and US | 1036/1046=99.0% |
| HSS-12 | Rongkavilit 2010^125^ | 70 | Mean (SD): 22.8 (2.1) | 37.1% man  58.6% woman  4.2% nonbinary | Thai youth living with HIV aged 16–25 years | Mean (SD): 1.9 (1.8) | Not mentioned | Convenient sample of patients who were being followed at an HIV clinic at King Chulalongkorn Hospital and two clinics at the Thai Red Cross AIDS Research Center in Bangkok. | Thailand | 70/74=94.6% |
| HSS-12 | Reinius 2017^126^ | 880 | Mean (Range): 47.9 (18-82) | 26% woman | 18yrs+ HIV+ diagnosed with HIV >6 months | Not mentioned | Not mentioned | Participants were recruited consecutively at 15 different centres for HIV care across Sweden, resulting in a total of 1096 valid responses. | Sweden | response rate ranging between 36 and 70% for different centres |
| HSS-12 | Luz 2020^127^ | Grindr: 114  Social media: 164  Hornet: 1824 | Mean (SD):  Grindr: 38.6 (10.1);  Social media: 43.7 (12.3);  Hornet: 37.8 (9.9). | Grindr: 100% man; Social media: 54.3% man, 38.4% woman; Hornet: 98.4% man, 1.6% nonbinary | 18yrs+ residency in Brazil, and self-report of HIV-infection | Not mentioned | Not mentioned | Three convenience samples of adult Brazilians were recruited to complete a web-based survey through advertisements on different platforms. The Grindr platform was used to recruit GBM whereas social media apps (Facebook and WhatsApp) were used to recruit women and men. The Hornet platform was used to recruit a second sample of GBM. | Brazil | Not mentioned |
| HSS-39 | Lindberg 2014^128^ | 194 | Mean (SD): 48.8 (11.7) | 43.8% woman  56.2% man | 18yrs+ HIV+ | Not mentioned | Not mentioned | Recruited from the Department of Infectious Diseases at the Karolinska University Hospital in Stockholm, Sweden; the sample of patients listed at the clinic was judged representative regarding gender distribution and immigration status for people living with HIV in Sweden. | Sweden | 53% |
| HSS-30 | Fuster-RuizdeApodaca 2015^129^ | 597  Group 1:40  Group 2: 557 | Mean (SD, Range): 1: 42.98 (5.95);  2: 43.43 (8.09, 18-76). | 70.8% man  28% woman  1.3% nonbinary | 18yrs+ HIV+ not having any severe psychiatric or cognitive disorder | Mean (SD): 13.5 (7.6) | Median (SD): CD4+ T-cells count (cells/mm3): 557.8 (288.7); Undetectable viral load: 62.7%; Taking ART: 88.2%. | During patients consultations or when attending to diverse services. | Spain | Not mentioned |
| HSS-10 | Kagiura 2020^130^ | 451 | Mean (SD): 47.7 (10.7) | 90.9% man | 20yrs+ HIV+ | Not mentioned | Not mentioned | Nine hospitals in Japan. | Japan | 451/819=55.1% |
| HASI-P | Holzemer 2007^131^ | 1477 | Mean (SD, Range): 36.09 (8.9, 16-69) | 74.1% man | HIV+ adults | Mean (SD, Range): 3.35 (2.84, 0-18) | Taking ARVs now: 46.2%. | In South Africa, field workers interviewed PLWA in one region in their homes, at clinics and in PLWA support groups. In Malawi, data collectors worked with the National Association of People Living with HIV/ AIDS and most data collection took place at support group meetings. In Swaziland, PLWA support groups were used and participants received a cash incentive. In Lesotho, data collection was conducted by field workers in all regions of the country and participants received a cash incentive. In Tanzania, research assistants visited PLWA support groups and participants received a cash incentive. | South Africa, Malawi, Swaziland, Lesotho, and Tanzania | Not mentioned |
| HASI-P | Maluccio 2017^132^ | 904 | Mean (SD): 39.1 (9.7) | 71.6% woman | 18yrs+ HIV+ non-pregnant naïve | Mean (SD): 2.2 (1.8) | Median (SD): CD4+ T-cells count (cells/mm3): 338.2 (63.2). | Gulu and Soroti district TASO clinics. | Uganda | Not mentioned |
| IHSM | Sayles 2008^133^ | 202 | Mean (Range): 43 (18-76) | 49.5% man 49.0% woman  1.5% nonbinary | 18yrs+ HIV+ | Not mentioned | Patients on HAART: over 70%. | Participants recruited from five community-based organizations providing outreach and social services to HIV-positive women and men, as well as from two HIV specialty clinics in a large U.S. city. | USA | Not mentioned |
| IA-RSS | Kalichman 2009^134^ | 456 | Median (IQR): 35 (29-40) | 69.5% woman | HIV+ adults | Not mentioned | Median (IQR): CD4+ T-cells count (cells/mm3): 203 (129–289); Currently on ART: 56.8%. | Recruited from the Mbarara Immune Suppression Syndrome Clinic | Uganda | Not mentioned |
| IA-RSS | Tsai 2013^135^ | South Africa: 1068;  Swaziland: 1087;  USA: 219. | Not mentioned | South Africa: 61% woman;  Swaziland: 67% woman;  USA: 23% woman. | HIV+ adults | Mean (SD): South Africa: 3.7 (2.4); Swaziland: 1.9 (1.4); USA: 12.5 (6.2). | Taking ARVs:  South Africa: 48%;  Swaziland: 78%;  USA: 63%. | Data were collected through confidential surveys administered in three cities/countries: Cape Town South Africa, nationally in Swaziland (from the City of Manzini and the regions of Hhohho, Lubombo and Shiselweni), and Atlanta Georgia USA recruited from community support and HIV treatment services. | South Africa, Swaziland, and USA | Not mentioned |
| IA-RSS | Geibela 2020^136^ | Cambodia: 1207  Dominican Republic: 891  Uganda: 391  Tanzania: 527 | Mean (SD): Cambodia: 44.9 (9.9); Dominican Republic: 39.1 (10.5); Uganda: 36.2 (10.9); Tanzania: 33.4 (8.5) | Cambodia: 60.8% woman; Dominican Republic: 58.1% woman; Uganda: 59.9% woman; Tanzania: 100.0% woman | PLHIV | Mean (SD): Cambodia: 11.0 (4.8); Dominican Republic: 7.1 (5.4); Uganda: 6.9 (6.6); Tanzania: NC. | Not mentioned | PLHIV in Uganda (six districts, 2017), the Dominican Republic (six provinces, 2018) and Cambodia (six provinces, 2019), FSWs in Tanzania (two districts, 2018–2019). | Cambodia, Dominican Republic, Uganda, and Tanzania. | Not mentioned |
| ISAT | Phillips 2011^137^ | 255 | Not mentioned | 100% woman | HIV+ adults | Not mentioned | Not mentioned | Recruited from ten community-based HIV/AIDS service organizations in three states. | USA | 255/280=91.1% |
| HARSI | Neufeld 2012^138^ | 217 | Not mentioned | 50.6% man  48.0% woman  1.5% nonbinary | 18yrs+ HIV+ had experienced sexual abuse as a child and/or adolescent | Not mentioned | Not mentioned | Recruited from community organizations and health care clinics serving those with HIV/AIDS in New York City. | USA | 271/333=81.4% |
| SEP-HASS | Zelaya 2012^139^ | 200 | Range: 18-40 | 44% man  50% woman  6% nonbinary | 18-40 years diagnosed with HIV for at least one year | Not mentioned | Diagnosis of AIDS: 34%;  Receiving ART: 52.1%. | Recruited by simple random sampling from HIV positive network meetings (support groups) in low-income communities of Chennai. | India | Not mentioned |
| HIV-SM | Earnshaw 2013^140^ | 95 | Mean (SD): 49.34 (9.45) | 49.5% man  50.5% woman | 18yrs+ HIV+ | Mean (SD): 15.56 (6.50) | CD4+ T-cells count (cells/mm3) < 200: 10.0%;  Chronic Illness Comorbidity: 77.9%; ARV Adherent: 35.8%; ARV Non-Adherent: 48.4%; ARV Not on Treatment: 15.8%. | Data were drawn from a study of psychosocial determinants of retention in HIV medical care at a community clinic providing integrated HIV care in the Bronx, New York. | USA | 95/101=94.1% |
| HA-SAL-GBT | Molina 2013^141^ | 170 | Mean (SD, Range): 39.8 (7.9, 21–59) | 100% gay/ bisexual men and trans-women | HIV+ GBT of Latin American descent | Mean (SD, Range): 15.80 (5.80, 7–31) | AIDS: 55%; One Drug Use: 24%; Multiple drugs Use: 32%. | Enrolled in Chicago and San Francisco. Participants were recruited using respondent-driven sampling, a social network, peer referral method. | USA | Not mentioned |
| VR-HARSSR | Kipp 2015^142^ | 85 | Median (IQR): 45 (19) | 74.1% man  25.9% woman | 18yrs+ HIV+ with at least one prior medical provider visit at the clinic | Median (IQR): 10 (12) | Taking ART: 89.4%. | The study was conducted at the Vanderbilt University Medical Center outpatient HIV clinic in Nashville, Tennessee. | USA | 85/211=40.28% |
| MAH | Ross 1994^143^ | Adelaide:38  Sydney: 69 | Mean (SD): Adelaide: 39.4(9.0), Sydney: 37.0(7.5). | 100% man | HIV+ men reported homosexual contact as the mode of infection | Not mentioned | AIDS: 33.7%. | The Adelaide sample was obtained as part of a research study into immune function, and the Sydney sample was obtained at the Albion Street Clinic, the main HIV ambulatory care centre in the city. | Australia | Not mentioned |
| SS-HIV | Niu 2016^144^ | 667 | Mean (SD, Range): 31.9 (10.6, 18-76) | 7.3% woman | 18yrs+ HIV+ | Newly diagnosed (< 1 month): 83.5% | CD4+ T-cells count (cells/mm3) < 200: 12.6%, HIV-related clinical symptoms: 36.7%. | People living with HIV who attended the free voluntary counseling and testing clinic of Changsha CDC and the Changsha Infectious Disease Hospital. | China | 667/700=95.3% |
| PSSHIV | Su 2008^145^ | 215 | 30-39: 53.3% | 63% man | 18-60 yrs old HIV+ known about their HIV status for at least 2 months | Not mentioned | CD4+ T-cells count (cells/mm3)≤200: 31%, >200: 31.5%; 40.5% Receiving HAART. | The participants were being recruited by convenience sampling method in the two study sites (Hengyang in Hunan Province and Shenzhen in Guangdong Province). | China | Not mentioned |
| Screenphiv | Remor 2012^146^ | 84 | Not mentioned | Not mentioned | HIV+ adults | Not mentioned | Not mentioned | From different regions of Spain. | Spain | Not mentioned |
| Screenphiv | Remor 2016^147^ | 744 | Mean (SD, Range): 43.04 (9.44, 18-82) | 72% man | 18yrs+ HIV+ | Diagnosed with HIV infection at least 6 months before the date of evaluation. | Not mentioned | Contacted through hospitals, non-governmental organizations, and patient associations. | Spain | 744/757=98.3% |
| ISCS | Golub 2013^148^ | 129 | Mean (SD, Range): 42.46 (10.12, 18-54) | 100% man | 18yrs+ HIV+ MSM | HIV+ >10 years: 50%; 5-9 year: 17%; 1-4 year: 25%; <1 year: 9%. | Not mentioned | Participants were ethnically diverse HIV-positive men surveyed at a series of gay, lesbian, and bisexual community events in New York City. | USA | 129/133=96.99% |
| IHIV | Buscher 2015^149^ | 356 | <35: 12%;  35-49: 52.9%;  >=50: 35.1% | 64.1% man  35.9% woman | 18yrs+ HIV+ prescribed ART for at least the last 30 days | 0-2: 8.5%; 2-5: 15.9%; 5-10: 21.5%; 10-15: 22.9%; ≥15: 31.2%. | CD4+ T-cells count (cells/mm3)> 350: 58%; Undetectable viral load (copies/mL) <400: 71.9%. | Thomas Street Health Center, an HIV clinic in Houston, Texas. | US | Not mentioned |
| HIVMS | Audet 2015^150^ | 125 | Mean (SD): 41.7 (2.0) | 73% man | HIV+ adults | Mean (SD): 8.1 (5.6) | CD4+ T-cells count (cells/mm3) (N=75): 432 (338); Years with AIDS (N=40): 2.3 (0.5). | All were receiving care for HIV at the Comprehensive Care Center in Nashville, TN. | US | Not mentioned |
| PLHIV-RS | Gottert 2019^151^ | Cameroon: 400; Senegal: 406; Uganda: 401. | Mean (Range): Cameroon: 37.9 (18-69); Senegal: 42.1 (18–70); Uganda: 36.2 (18–81). | Cameroon: 72.2% woman;  Senegal: 79.0% woman;  Uganda: 59.9% woman. | HIV+ adults | Mean (Range): Cameroon: 7.8 (0–27); Senegal: 12.6 (0–23); Uganda: 6.8 (0–58). | Not mentioned | Conducted in collaboration with Metabiota and Réseau Cameronais des Associations de Personnes Vivant avec le VIH in Cameroon, Enda Santé and Réseau National des Associations de PVVIH du Sénégal in Senegal, and the National Forum of PLHIV Networks in Uganda. used a combination of two non-probabilistic sampling methods—venue-based and snowball sampling—to enroll a diverse group of PLHIV. | Cameroon, Senegal, and Uganda | Not mentioned |
| BIS | Martinez 2005^152^ | 147 | Mean (Range): 42 (22-65) | 21% woman | HIV+ adults | 1 month to 19 years | 46% diagnosis of AIDS;  Mean (Range): CD4+ T-cells count (cells/mm3): 448 (9-1898); Mean (Range): HIV RNA (log10 copies/ml): 4.51 (1.26-6.03). | Recruited from two sites (a public hospital HIV clinic and a university hospital HIV clinic) in California. | USA | 147/149=98.7% |
| OCLS | Lee 2006^153^ | 107 | Median (range): 43 (26–66) | 93% man  7% woman | HIV+ adults | Not mentioned | Median (Range): CD4+ T-cells count (cells/mm3): 491 (125–1485); HIV RNA (log10 copies/mL): 2.60 (1.60–5.59). | All adult HIV-infected patients attending the Owen Lipid/ Lipodystrophy Clinic for monitoring and/or treatment of lipid abnormalities or LD were eligible for participation in this study. | USA | Not mentioned |
| ACTG-ABCD | Guaraldi 2006^154^ | LD+: 295  LD-: 35 | Mean (SD): LD+: 44 (7)  LD-: 39 (6) | LD+: 26% woman; LD-: 33% woman | 18yrs+ HIV+ taking ART | Not mentioned | CD4+ T-cells count (cells/mm3): LD+: 543 (283), LD-: 526 (243); Viral load log10 (copies/ml): LD+: 2.4 (1.04), LD-: 2.7 (1.2); Months on ARV: LD+: 98 (42), LD-: 83 (16). | This was a cross-sectional study of HIV-infected patients, seen consecutively at the HIV outpatient clinic of Modena Teaching Hospital in Italy. | Italy | Not mentioned |
| ACTG-ABCD-SF | Blashill 2014^155^ | 106 | Mean (SD): 47.5 (7.8) | 100% man | 18yrs+ HIV+ MSM | Mean (SD): 13.1 (8.2) | Years taking HIV medications: 10.1 (6.8); CD4+ T-cells count (cells/mm3): 674 (281); Viral load (log10 copies/ml): 1.9 (0.4). | Participants were 106 HIV-infected MSM recruited from the infectious disease clinic at Massachusetts General Hospital and Fenway Health—a community health center in Boston, MA serving the gay, lesbian, bisexual, and transgender community. | USA | 106/124=85.48% |
| FAI | Wu 2016^156^ | 96 | Mean (SD, Range): 48.8 (8.3, 31-84) | 87.5% man  12.5% woman | 18yrs+ HIV+ | Not mentioned | Viral load (log10 copies/ml): 4.7 (4.5); Median CD4+ T-cells count (cells/mm3): 435.47 (281.0); Years of ART: 9.4 (4.5). | Patients with lipoatrophy were recruited from the Johns Hopkins Moore Clinic, the lipodystrophy clinic of Marcus Conant, MD at the University of California San Francisco Medical Center, and the lipodystrophy clinic of Peter Englehard, DO in Miami, Florida. | USA | Not mentioned |
| MAS | Aversa 1998^157^ | 62 | Mean (SD): 35.87 (6.99) | 74% man  26% woman | HIV+ taking ART | Not mentioned | Asymptomatic: 7%; Somewhat symptomatic: 32%; More symptomatic: 61%. | The sample was obtained by collaborating with several community-based AIDS networks in the state of Florida. | USA | Not mentioned |
| HIVTSQ | Woodcock 2001^158^ | USA: 127  Canada: 23 | Mean (SD, Range): 37.3 (8.4, 21-62) | 89.3% man | HIV-1+ adults | Not mentioned | Viral load (log10 copies/ml): 4.1 (0.76); CD4+ T-cells count (cells/mm3): 385.8 (178.2). | 127 were recruited at 29 trial sites in the USA and 23 at 11 trial sites in Canada. | USA | 150/338=44.38% |
| HIVTSQ-s | Woodcock 2006^159^ | 152 | Mean (SD, Range): 41.2 (7.9, 24-69) | 84.9% man  15.1% woman | HIV+ taking ART | Not mentioned | Viral load (log10 copies/ml): 4.09 (0.82, 1.69-5.78). | A clinical trial of an investigational product for treatment of HIV, just before randomization to one of three treatments. | USA | Not mentioned |
| TES | Webb 2001^160^ | 43 | Mean (SD): 38.6 (7.7) | 93% man | advanced HIV+ | Not mentioned | Not mentioned | Patients were recruited from a dedicated HIV facility at a London teaching hospital to undertake an anonymous self-completed questionnaire. | UK | Not mentioned |
| SIS | Green 2002^161^ | 547 | Mean (SD): 42.4 (8.16) | 91.4% man | HIV-1+ adults or adolescents | Not mentioned | Not mentioned | Patients were recruited from clinical centers in Australia, Canada, the United States, Europe, and Latin America. | Three countries and two continents | Not mentioned |
| QUOTE-HIV | Hekkink 2003^162^ | 44 | Mean (SD): 42.8 (7.6) | 84.09% man | HIV+ adults | Mean (SD): 7.1 (4.2) | Not mentioned | HIV infected population in Amsterdam. | Netherlands | 44/80=55% |
| AHHCP | Bodenlos 2004^163^ | 129 | Median (IQR): 38 (18-61) | 57.36% man | 18yrs+ HIV+ | Months Mean (SD, Range): 72.49 (50.81, 1-252) | Viral load (copies/ml) <5000: 57%, 5001-50000: 21%, >50000: 22%; CD4+ T-cells count (cells/mm3) <100: 16%; 101-250:19%; 251-500:34%; >500: 31%. | Participants were recruited from an HIV clinic within a teaching hospital in a southeastern city. | USA | 129/160=80.625% |
| AGAS | Holstad 2010^164^ | GBL: 247  KHARMA: 207 | Mean:  GBL: 41  KHARMA: 43 | GBL: 32.4% woman; KHARMA: 100% woman. | GBL: HIV+; KHARMA: HIV+ women. | Years:  GBL: Range: 1-21, >10 37%; KHARMA: >10: 46.9%. | Not mentioned | Data from the baseline assessments of two separate, National Institutes of Health funded studies were used for this project. Both studies focused on promoting ART adherence. Participants for both studies were recruited from HIV treatment sites in a large Southeastern city. | USA | Not mentioned |
| HCR | Bova 2006^165^ | 99 | Mean (SD, Range): 42.9 (7.8, 22-62) | 50.5% man | HIV+ adults currently followed by an HCP. | Not mentioned | Asymptomatic: 57.6%; Symptomatic: 17.2%; AIDS: 18.2%. | Recruited from HIV primary care sites and from the ATHENA cohort by mail, phone, or directly by a team member after a primary care visit. | USA | Not mentioned |
| HMRS | Balfour 2007^166^ | 142 | Mean (SD): 40.46 (8.84) | 86.6% man  13.4% woman | HIV+ not currently taking ART for at least 1 month | Mean (SD): 4.66 (5.40) | CD4+ T-cells count (cells/mm3): 330.27 (200.04); Viral load (copies/ml): 74238.20 (115667.41). | Participants were drawn from two samples of HIV-positive outpatients seen at a university teaching hospital. | Canada | Not mentioned |
| SECope | Johnson 2007^167^ | 233 | Mean (SD): 46.0 (8.2) | 86% man  12% woman  3% other | HIV+ adults on ART | Not mentioned | Undetectable Viral Load: (n=154) (66%); CD4+ T-cells count (cells/mm3): 416 (251). | This administration took place in the context of a baseline interview for a clinical trial of a side effects coping intervention. | USA | Not mentioned |
| HTOS | Brennan 2009^168^ | 346 | Median (IQR): 43 (38–48) | 100% man | 18yrs+ HIV+ MSM | Median (IQR): 12 (7–16) | Viral load (copies/ml) <50: 70%; >50: 30%. | MSM attending a two-day sexual health intervention in Boston, Los Angeles, Houston, and New York, four cities with high HIV incidence. | USA | 346/539=64.19% |
| HIV-MT-SES | Erlen 2010^169^ | 326 | Mean (SD, Range): 43.84 (7.97, 20-66) | 69.3% man | 18yrs+ HIV+ | Not mentioned | Not mentioned | Participants were recruited from HIV/AIDS primary care clinics in western Pennsylvania and eastern Ohio in the USA. | USA | Not mentioned |
| BEHKA-HIV | Osborn 2010^170^ | 204 | Mean (SD): 40.1 (9.2) | 79.9% man | Consecutive HIV+ | Not mentioned | Not mentioned | Eligible patients had been prescribed one or more antiretroviral medications, and were receiving medical care through outpatient infectious disease clinics at Northwestern Memorial Hospital in Chicago, Illinois and Louisiana State University Health Sciences Center in Shreveport, Louisiana. | USA | Not mentioned |
| HTRM | Fernández 2011^171^ | 201 | Mean (SD): 20.5 (2.5) (< 18 years old: 11%) | 67% man | Adolescents and young adults living with HIV | Not mentioned | Treatment experienced: 34%. | Enrolled adolescents and young adults living with HIV from 15 adolescent medicine clinics in the United States affiliated with the ATN. | USA | Not mentioned |
| HTRFS | Claborn 2015^172^ | 96 | Not mentioned | 85.4% man | 18yrs+ HIV+ had been prescribed an ART regimen for at least 6 months | Not mentioned | Mean (SD, Median, Range):  Prescribed ART: 10.12 (7.86, 9, <1-43) | Conducted in an outpatient community health center that primarily served PLWH. | USA | Not mentioned |
| HECCS | Logie 2017^173^ | 173 | Mean (SD): 40.7 (8.8) | 100% woman | 18yrs+ HIV+ | Not mentioned | Not mentioned | Spaces where HIV services were accessed nine peer research assistants who were ACB WLWH in three regions of Ontario. | Canada | Not mentioned |
| SSI | Renwick 1999^174^ | 120 | Mean (SD, Range): 36.9 (8.1, 22-63) | 89.17% man  10.83% woman | 18yrs+ HIV+ | Not mentioned | Diagnosis of AIDS: 29%; HIV+ with symptoms: 35%; HIV+ with no symptoms: 35%. | Drawn from three hospital-based clinics (62.5%) and two drop-in centres (37.5%) treating individuals with HIV in Metropolitan Toronto. | Canada | Not mentioned |
| USII-HIV | Ingram 1999^175^ | OSU: 96  VCU: 175 | Mean (SD, Range):  OSU: 36.03 (8.01, 21-62);  VCU: 37.70 (7.58, 21-61). | OSU: 90% man  VCU: 67% man | 21yrs+ HIV+ | Mean (SD, Range):  OSU: 4.21 (2.79, 1month-12 yrs);  VCU: not report. | CD4+ T-cells count (cells/mm3): OSU: 218.24 (224.90, 0-974); VCU: 312 (240, 1-1100). CD4+ T-cells count (cells/mm3) < 50: OSU: 37%; VCU: 13%. | The sample was drawn from two sources: The Ohio State University Adult AIDS Clinical Trials Unit and the Medical College of Virginia Infectious Disease Clinic at Virginia Commonwealth University. | USA | Not mentioned |
| PSS-HIV | Cortes 2014^176^ | Chile: 24  UK: 12 | Mean (SD): Chile: 35.87 (8.4);  UK: 40.83 (8.1). | Chile: 4.2% woman  UK: 41.7% woman | 18yrs+ HIV+ | Not mentioned | Not mentioned | Two samples of PLWHA; one in the UK and the other in Chile. PLWHA from the UK were recruited at the Derbyshire Positive Support; a peer support group for PLWHA. PLWHA from Chile were recruited at the Universidad XX Clinical Hospital. | UK and Chile | Not mentioned |
| HIV-ASES | Johnson 2007^177^ | Study 1: 264;  Study 2: 2848. | Mean (SD): Study 1: 46.5 (8.1); Study 2: 42.5 (7.6). | Study 1: 86% man;  Study 2: 74.1% man. | HIV+ taking ART | Not mentioned | Study 1: CD4+ T-cells count (cells/mm3): 377.5 (247.9);  Study 2: Undetectable Viral Load: 75.0%;  CD4+ T-cells count (cells/mm3): 427.6 (292.2). | HIV+ individuals in the San Francisco Bay Area were screened for recruitment into the Balance Project, a clinical trial of an HIV treatment side effects coping intervention. HIV+ individuals in San Francisco, Los Angeles, New York and Milwaukee were screened for inclusion in the Healthy Living Project, a clinical trial of a comprehensive cognitive-behavioral sexual risk reduction intervention. | USA | Not mentioned |
| PHIVSMS | Wallston 2011^178^ | 125 | Mean (SD): 41.7 (2.0) | 73% man | HIV+ adults | Mean (SD): 8.1 (5.6) | CD4+ T-cells count (cells/mm3): 432 (338); Years with AIDS: 2.3 (0.5); Diagnosed with AIDS: 31%. | Adults who were receiving care for HIV at the Comprehensive Care Center in Nashville, TN and who were not experiencing posttraumatic stress disorder or did not have a diagnosis of psychosis and were not currently taking antipsychotic medications were randomized into either an expressive writing or a control writing condition. | USA | Not mentioned |
| HIV-SMS-W | Webel 2012^179^ | 260 | Mean (SD): 46 (9.3) | 100% woman | 21yrs+ HIV+ women | Mean (SD): 15 (7.5) | Undetectable Viral Load: 50%; Median (IQR) CD4+ T-cells count (cells/mm3): 484 (233-780); Median (IQR) HIV RNA (copies/mm3): 2027.5 (400-10000). | Recruited from HIV Clinics and AIDS Service Organizations in Northeast Ohio and the San Francisco Bay Area in California. | USA | Not mentioned |
| HIV-IM | Nelsen 2012^180^ | 287 | Mean (SD): 50.8 (9.9) | 89.9% man  10.1% woman | HIV+ | <1: 4.9%; 1–10: 34.8%; >10: 60.3%. | CD4+ T-cells count (cells/mm3): 468.3 (286.8, 4.0–1491.0); Viral load (log10 copies/mL): 2.3 (1.2, 1.7–6.9);  Patients on HAART: 87.1%. | Participants were recruited from HIV clinics at the Michael E. DeBakey VA Medical Center and the Thomas Street Health Center in Houston, Texas. Both clinics exclusively serve adult patients with HIV. | USA | 287/311=92.3% |
| HIVESS | Gray 2016^181^ | Stage 2: 133  Stage 4: 107 | Stage 2: 50.46 (10.88); Stage 4: 52.51 (11.42) | Stage 4: 75.7% man; 24.3% woman. | HIV+ at least 10 years of therapy and no co-infection or other diseases | Not mentioned | Not mentioned | PLHIV were recruited from three French hospitals by hospital staff and researchers following certain inclusion criteria. | France | Not mentioned |
| HSM-SEWS | Webel 2011^182^ | 89 | Mean (Range): 47.0 (27–72) | 83% woman  16% nonbinary | 21yrs+ HIV+ women | Mean: 24.1 | CD4+ T-cells count (cells/mm3): 464.4 (257.6);  HIV RNA (1000/mL): 3.75; Current ART Use: 72.7%. | The study was conducted at several HIV clinics in the San Francisco Bay area. | USA | Not mentioned |

Abbreviations: PROM, Patient Reported Outcome Measure; AIDS, Acquired Immune Deficiency Syndrome; HIV, Human Immunodeficiency Virus; ART, antiretroviral therapy; PLWHA, people living with HIV/AIDS; IDU, Intravenous Drug Use; HAART, highly active antiretroviral treatment; PLWH, people living with HIV; RCT, randomized controlled trial; CDC, Centers for Disease Control and Prevention; SD, standard deviation; IQR, interquartile range;

MOS-HIV, Medical Outcomes Study-HIV Health Survey; HOPES, HIV Overview of Problems Evaluation System; HIV-QoL, HIV-related Quality of Life Questions; AIDS-HAQ, AIDS Health Assessment Questionnaire; HIV-PARSE, HIV Patient Reported Status and Experience; HRQOL, Health-Related Quality of Life; FAHI, Functional Assessment of HIV Infection; GHSA, General Health Self-Assessment; HIV-QL31, HIV Quality of Life 31 items Scale; HAT-QoL, HIV/AIDS Targeted QOL Instrument; MQoL-HIV, Multidimensional QOL for Patients with HIV/AIDS; LWHIVS, Living with HIV Scale; WHOQOL-HIV, World Health Organization Quality of Life HIV; ISSQoL, Instituto Superiore di Sanità Quality of Life; HIV-SQUAD, Symptom Quality of Life Adherence; PROQOL-HIV, Patient Reported Outcome Quality of Life-HIV Questionnaire; PozQol, Poz Quality of Life; RSC, Riverside Symptom Checklist; HSI, HIV Symptom Index; HAT, HIV Assessment Tool; SSC-HIV, Sign and Symptom Checklist for HIV; HCSUS-SM, HIV Cost and Services Utilization Study Symptom Measure; HIV-SI/SDM, HIV Symptom Index or Symptoms Distress Module of the ACTG; HRFS, HIV-Related Fatigue Scale; HDQ, HIV Disability Questionnaire; ISS-HIV-SS, Istituto Superiore di Sanità-HIV symptoms scale; HSS-40, HIV Stigma Scale; HASI-P, HIV/AIDS Stigma Instrument-PLWA; IHSM, Internalized HIV Stigma measure; IA-RSS, Internalized AIDS-Related Stigma Scale; ISAT, Internalized Stigma in those with HIV/AIDS; HARSI, HIV and Abuse Related Shame Inventory; SEP-HASS, Self, experienced, and perceived HIV/AIDS stigma scales; HIV-SM, HIV stigma mechanisms; HA-SAL-GBT, HIV/AIDS stigma assessment for Latino gay men, bisexual men and transgender women living with HIV; VR-HARSSR, Van Rie HIV/AIDS-Related Stigma Scale -Revised for use in the USA; MAH, The Mental Adjustment to HIV scale; SS-HIV, HIV/AIDS Stress Scale; PSSHIV, Perceived Stress Scale among PLWHA; ISCS, Impact on Self-Concept Scale; IHIV, Impact of HIV; HIVMS, HIV Meaningfulness Scale; PLHIV-RS, People Living with HIV Resilience Scale; BIS, Body Image in Patients with HIV/AIDS; OCLS, Owen Clinic Lipodystrophy Scale; ACTG-ABCD, Adult AIDS Clinical Trial Group's Assessment of Body Change and Distress; ACTG-ABCD Short form, ACTG-ABCD-SF; FAI, Facial Appearance Inventory; MAS, Medication Attribution Scale; HIVTSQ, HIV Treatment Satisfaction Questionnaire; TES, Treatment-related Empowerment Scale; SIS, Subcutaneous Injection Survey; QUOTE-HIV, Quality of care through the patient’s eyes; AHHCP, Attitudes toward HIV Health Care Provider scale; AGAS, Antiretroviral General Adherence Scale; HCR, Health Care Relationship Trust Scale; HMRS, HIV Medication Readiness Scale; HTOS, HIV Treatment Optimism Scale; HIV-MT-SES, HIV Medication Taking Self-Efficacy Scale; BEHKA-HIV, Brief Estimate of Health Knowledge and Action-HIV version; HTRM, HIV Treatment Readiness Measure; HTRFS, HIV Treatment Regimen Fatigue Scale; HECCS, HIV Engagement in and Continuity of Care Scale; SSI, Social Support Inventory; USII-HIV, Unsupportive Social Interactions Inventory –HIV version; PSS-HIV, Perceived Social Support for HIV; HIV-ASES, HIV Treatment Adherence Self-Efficacy Scale; PHIVSMS, Perceived HIV Self-Management Scale; HIV-SMS-W, HIV Self-management Scale (Women); HIV-IM, HIV Intention Measure; HIVESS, HIV Exercise Stereotypes Scale; HSM-SEWS, HIV Symptom Management Self-Efficacy for Women Scale.

## Table S5. Methodological Quality Assessment of the Included Records

| **PROM (Author Year)** | **PROM development** | **Content validity** | **Structural validity** | **Internal consistency** | **CCV/MI** | **Reliability** | **Measurement error** | **Criterion validity** | **HTCV** | **Responsiveness** |
| --- | --- | --- | --- | --- | --- | --- | --- | --- | --- | --- |
| MOS-HIV (Carretero 1996)^31^ |  |  |  | V |  |  |  | N/A | D |  |
| MOS-HIV (Murri 1997)^32^ |  |  |  | V |  |  |  | N/A | I |  |
| MOS-HIV (Revicki 1998)^33^ |  |  | V | V |  | D |  | N/A | D |  |
| MOS-HIV (Badia 1999)^34^ |  |  |  | V |  | V |  | N/A | D |  |
| MOS-HIV (Holmes 1999)^35^ |  |  |  | V |  |  |  | N/A | D |  |
| MOS-HIV (Scott-Lennox 1999)^36^ |  |  | A | V |  |  |  | N/A | D |  |
| MOS-HIV (Delate 2001)^37^ |  |  |  | V |  |  |  | N/A | D |  |
| MOS-HIV (Paton 2002)^38^ |  |  | I | V |  |  |  | N/A | D |  |
| MOS-HIV (Schifano 2003)^39^ |  |  |  | V |  |  |  | N/A | D |  |
| MOS-HIV (Ichikawa 2004)^40^ |  |  | A | V |  |  |  | N/A | D |  |
| MOS-HIV (Lau 2006)^41^ |  |  | A | V |  |  |  | N/A | D |  |
| MOS-HIV (Taylor 2009)^42^ |  |  | A | V |  |  |  | N/A | D |  |
| MOS-HIV (Henderson 2010)^43^ |  |  | V | I |  |  |  | N/A | V |  |
| MOS-HIV (Stasinopoulou 2010)^44^ |  |  |  | V |  |  |  | N/A | D |  |
| MOS-HIV (Chariyalertsak 2011)^45^ |  |  |  | V |  |  |  | N/A | I |  |
| MOS-HIV (Hsiung 2011)^46^ |  |  | V | V |  |  |  | N/A | I |  |
| MOS-HIV (Epino 2012)^47^ |  |  | A | V |  |  |  | N/A | D |  |
| MOS-HIV (Huang 2012)^48^ |  |  |  | V |  |  |  | N/A | D |  |
| MOS-HIV (Marzieh 2017)^49^ |  |  | I | V |  |  |  | N/A |  |  |
| MOS-HIV (Liu 2018)^50^ |  |  | V | V |  | A |  | N/A | D |  |
| MOS-HIV (Shim 2018)^51^ |  |  | A | V |  |  |  | N/A | D |  |
| MOS-HIV-29 (Stangl 2012)^52^ |  |  | A | V |  |  |  | N/A | D | D |
| MOS-HIV-17 (McDonnell 2000)^53^ |  |  |  | V |  |  |  | N/A | D |  |
| HOPES (Schag 1992)^54^ |  |  |  | V |  |  |  | N/A | I |  |
| HOPES (De Boer 1996)^55^ |  |  |  | V |  |  |  | N/A | D | D |
| HIV-QoL (Cleary 1993)^56^ |  |  |  | V |  |  |  | N/A |  |  |
| AIDS-HAQ (Lubeck 1997)^57^ |  |  |  | V |  |  |  | N/A | V | D |
| HIV-PARSE (Bozzette 1994)^58^ |  |  |  | V |  |  |  | N/A |  |  |
| HIV-PARSE-Brief (Bozzette 1995)^59^ |  |  |  | V |  |  |  | N/A |  |  |
| HRQOL (Hays 1995)^60^ |  |  | I | V |  |  |  | N/A | D |  |
| FAHI (Cella 1996)^61^ |  |  |  | V |  |  |  | N/A | I |  |
| FAHI (Peterman 1997)^62^ |  |  | V | V |  |  |  | N/A | D |  |
| FAHI (Viala-Danten 2010)^63^ |  |  |  | V |  |  |  | N/A | V | V |
| FAHI (Byrne 2013)^64^ |  |  |  | V |  |  |  | N/A | D |  |
| FAHI (Nyongesa 2017)^65^ |  | D | I | V |  |  |  | N/A | D |  |
| GHSA (Lenderking 1997)^66^ |  |  | A | V |  |  |  | N/A | D |  |
| HIV-QL31 (Leplège 1997)^67^ | I |  | A | V |  |  |  | N/A | D |  |
| HAT-QoL-42 (Holmes 1997)^68^ |  |  |  | V |  |  |  | N/A | D |  |
| HAT-QoL-42 (Holmes 1998)^69^ |  |  |  | V |  |  |  | N/A | D |  |
| HAT-QoL-30 (Holmes 1999)^35^ |  |  |  | V |  |  |  | N/A | D |  |
| HAT-QoL-34 (Holmes 2008)^70^ |  |  |  | V |  | V |  | N/A |  |  |
| HAT-QoL-34 (Taylor 2009)^42^ |  |  | A | V |  |  |  | N/A | D |  |
| HAT-Qol-34 (Mafirakureva 2016)^71^ |  |  |  | V |  |  |  | N/A | V |  |
| MQoL-HIV (Smith 1997)^72^ |  |  |  | V |  | I |  | N/A | D | V |
| MQoL-HIV (Badia 1999)^34^ |  |  |  | V |  | V |  | N/A | D |  |
| MQoL-HIV (Kemmler 2003)^73^ |  |  |  | V |  | A |  | N/A | D |  |
| MQoL-HIV (Remple 2004)^74^ |  | D |  | V |  | A |  | N/A | V |  |
| MQoL-HIV (Watanabe 2004)^75^ |  |  | A | V |  |  |  | N/A | I |  |
| LWHIVS (Holzemer 1998)^76^ | I |  | A | V |  |  |  | N/A | D |  |
| WHOQOL-HIV (Starace 2002)^77^ |  |  |  | V |  |  |  | N/A | D |  |
| WHOQOL-HIV (WHOQOL HIV Group 2004)^78^ |  |  | V | V |  |  |  | N/A |  |  |
| WHOQOL-HIV (Zimpel 2007)^79^ |  |  |  | V |  |  |  | N/A | D |  |
| WHOQOL-HIV (Canavarro 2011)^80^ |  |  |  | V |  |  |  | N/A | D |  |
| WHOQOL-HIV (Mweemba 2011)^81^ |  |  | I | V |  |  |  | N/A |  |  |
| WHOQOL-HIV (Olsen 2013)^82^ |  |  |  |  | I |  |  | N/A |  |  |
| WHOQOL-HIV (Reychler 2013)^83^ |  |  |  | V |  | V |  | N/A | V |  |
| WHOQOL-HIV-BREF (Saddki 2009)^84^ |  |  | A | V |  | A |  | N/A | D |  |
| WHOQOL-HIV-BREF (Hsiung 2011)^85^ |  |  | V | V |  |  |  | N/A | I |  |
| WHOQOL-HIV-BREF (O'Connell 2012)^86^ |  |  | V | V |  |  |  | N/A | D |  |
| WHOQOL-HIV-BREF (Tran 2012)^87^ |  |  | A | V |  |  |  | N/A | D |  |
| WHOQOL-HIV-BREF (Canavarro 2012)^88^ |  |  | V | V |  |  |  | N/A | D |  |
| WHOQOL-HIV-BREF (Pereira 2014)^89^ |  |  | A | V |  |  |  | N/A | D |  |
| WHOQOL-HIV-BREF (Meemon 2016)^90^ |  |  | V | V |  |  |  | N/A | D |  |
| WHOQOL-HIV-BREF (Salehi 2016)^91^ |  |  |  | V |  |  |  | N/A |  |  |
| WHOQOL-HIV-BREF (Tesfaye 2016)^92^ |  |  | V | V |  |  |  | N/A | D |  |
| WHOQOL-HIV-BREF (Zhu 2017)^93^ |  |  | V | V |  | A |  | N/A | D |  |
| WHOQOL-HIV-BREF (Fuster-Ruizde Apodaca 2019)^94^ |  |  | V | V |  |  |  | N/A | D |  |
| WHOQOL-HIV-BREF (Barger 2020)^95^ |  |  | V | V |  |  |  | N/A | D |  |
| ISSQoL (Bucciardini 2006)^96^ | D |  |  | V |  |  |  | N/A | D |  |
| HIV-SQUAD (Spire 2009)^97^ |  |  | A | V |  |  |  | N/A | D |  |
| PROQOL-HIV (Duracinsky 2012(1))^98^ | I |  |  |  |  |  |  | N/A |  |  |
| PROQOL-HIV (Duracinsky 2012(2))^99^ |  |  | A | V |  | D |  | N/A | D |  |
| PROQOL-HIV-43 (Duracinsky 2014)^100^ |  |  |  |  |  | D |  | N/A |  |  |
| PROQOL-HIV-38 (Lalanne 2016)^101^ |  |  | V | V |  |  |  | N/A | I |  |
| PozQol (Brown 2018)^102^ | D |  | V | V |  | V |  | N/A | A |  |
| RSC (Burgess1993)^103^ |  |  |  | V |  |  |  | N/A | V |  |
| HSI (Whalen 1994)^104^ |  |  |  | V |  | V |  | N/A | D | D |
| HAT (Nokes 1994)^105^ |  |  | I | I |  | D |  | N/A |  |  |
| SSC-HIV (Holzemer 1999)^107^ |  |  | A | V |  |  |  | N/A |  |  |
| SSC-HIV (Sousa 2006)^106^ |  |  | V |  |  |  |  | N/A |  |  |
| SSC-HIV-rev (Holzemer 2001)^108^ |  |  | A | V |  |  |  | N/A | D |  |
| HCSUS-SM (Mathews 2000)^109^ |  |  |  | V |  |  |  | N/A |  |  |
| HIV-SI/SDM (Justice 2001)^110^ | D |  |  |  |  |  |  | N/A | I |  |
| HIV-SI/SDM (Regnault 2009)^111^ |  |  |  |  | D |  |  | N/A |  |  |
| HIV-SI/SDM (Marc 2012)^112^ |  |  | V | V |  |  |  | N/A | D |  |
| HRFS (Barroso 2002)^113^ | I |  |  | V |  | D |  | N/A |  |  |
| HRFS (Pence 2008)^114^ |  |  |  | V |  |  |  | N/A | D |  |
| HRFS (Li 2016)^115^ |  |  |  | V |  | V |  | N/A | D |  |
| HDQ (O'Brien 2014)^116^ |  |  | A |  |  |  |  | N/A |  |  |
| HDQ (O'Brien 2015)^117^ |  |  |  | V |  | D |  | N/A | D |  |
| HDQ (Brown 2019)^118^ |  |  |  | V |  |  | D | N/A | I |  |
| HDQ (O'Brien 2019)^119^ |  |  |  | V |  | D |  | N/A | V |  |
| ISS-HIV-SS (Bucciardini 2016)^120^ |  |  | A | V |  |  |  | N/A | V |  |
| HSS-40 (Berger 2001)^121^ |  |  | A | V |  | A |  | N/A | V |  |
| HSS-40 (Rao 2008)^122^ |  |  |  |  | D |  |  | N/A |  |  |
| HSS-32 (Bunn 2007)^123^ |  |  | I | V |  |  |  | N/A | D |  |
| HSS-32 (Reinius 2018)^124^ |  |  | V |  | I |  |  | N/A |  |  |
| HSS-12 (Rongkavilit 2010)^125^ |  |  | D | V |  |  |  | N/A | D |  |
| HSS-12 (Reinius 2017)^126^ |  |  | V | V |  |  |  | N/A |  |  |
| HSS-12 (Luz 2020)^127^ |  |  | V | V | D |  |  | N/A | D |  |
| HSS-39 (Lindberg 2014)^128^ |  |  | I | V |  |  |  | N/A | I |  |
| HSS-30 (Fuster-RuizdeApodaca 2015)^129^ |  | D | V | V |  |  |  | N/A | V |  |
| HSS-10 (Kagiura 2020)^130^ |  |  | V | V |  |  |  | N/A | D |  |
| HASI-P (Holzemer 2007)^131^ |  |  | A | V |  |  |  | N/A | D |  |
| HASI-P (Maluccio 2017)^132^ |  |  | A | V |  |  |  | N/A | V |  |
| IHSM (Sayles 2008)^133^ |  |  | A | V |  |  |  | N/A | D |  |
| IA-RSS (Kalichman 2009)^134^ |  |  |  | V |  | D |  | N/A | D |  |
| IA-RSS (Tsai 2013)^135^ |  |  | A | V |  | D |  | N/A | I |  |
| IA-RSS (Geibel 2020)^136^ |  |  | V | V |  |  |  | N/A | D |  |
| ISAT (Phillips 2011)^137^ |  |  | A | V |  |  |  | N/A | V |  |
| HARSI (Neufeld 2012)^138^ |  |  | A | V |  | D |  | N/A |  |  |
| SEP-HASS (Zelaya 2012)^139^ |  |  |  | V |  |  |  | N/A | I |  |
| HIV-SM (Earnshaw 2013)^140^ |  |  |  | V |  |  |  | N/A | D |  |
| HA-SAL-GBT (Molina 2013)^141^ |  |  | I | V |  |  |  | N/A | D |  |
| VR-HARSSR (Kipp 2015)^142^ |  |  | A | V |  |  |  | N/A | V |  |
| MAHIVS (Ross 1994)^143^ |  |  | I | V |  |  |  | N/A |  |  |
| SS-HIV (Niu 2016)^144^ |  |  | V | V |  | A |  | N/A | D |  |
| PSSHIV (Su 2008)^145^ | I |  | A | V |  | A |  | N/A | D |  |
| Screenphiv (Remor 2012)^146^ | D |  | I | V |  |  |  | N/A |  |  |
| Screenphiv (Remor 2016)^147^ | D |  | V | V |  |  |  | N/A | V |  |
| ISCS (Golub 2013)^148^ |  |  | A | V |  |  |  | N/A | D |  |
| IHIV (Buscher 2015)^149^ |  |  | V | V |  |  |  | N/A |  |  |
| HIVMS (Audet 2015)^150^ |  |  | A | V |  | D |  | N/A | I |  |
| PLHIV-RS (Gottert 2019)^151^ | D |  | V | V |  |  |  | N/A | D |  |
| BIS (Martinez 2005)^152^ |  |  | A | D |  | A |  | N/A |  |  |
| OCLS (Lee 2006)^153^ |  |  | A | I |  |  |  | N/A |  |  |
| ACTG-ABCD (Guaraldi 2006)^154^ |  |  |  | V |  |  |  | N/A | D |  |
| ACTG-ABCD-SF (Blashill 2014)^155^ |  |  | A | V |  |  |  | N/A | D |  |
| FAI (Wu 2016)^156^ | D |  |  | D |  |  |  | N/A | D |  |
| MAS (Aversa 1998)^157^ |  |  |  | V |  |  |  | N/A | V |  |
| HIVTSQ (Woodcock 2001)^158^ |  |  | A | V |  |  |  | N/A | D |  |
| HIVTSQ-s (Woodcock 2006)^159^ |  |  | V | V |  |  |  | N/A | D |  |
| TES (Webb 2001)^160^ |  |  |  | V |  |  |  | N/A | D |  |
| SIS (Green 2002)^161^ |  |  | A | V |  |  |  | N/A | D |  |
| QUOTE-HIV (Hekkink 2003)^162^ |  |  |  | V |  |  |  | N/A |  |  |
| AHHCP (Bodenlos 2004)^163^ |  |  | A | V |  |  |  | N/A | V |  |
| AGAS (Holstad 2010)^164^ |  |  | A | V |  |  |  | N/A | D |  |
| HCR (Bova 2006)^165^ | I |  | A | V |  | A |  | N/A | D |  |
| HMRS (Balfour 2007)^166^ |  |  | A | V |  | D |  | N/A | D | V |
| SECope (Johnson 2007)^167^ | I |  | V | V |  | A |  | N/A | D |  |
| HTOS (Brennan 2009)^168^ |  |  | A | V |  |  |  | N/A |  |  |
| HIV-MT-SES (Erlen 2010)^169^ |  |  | V | V |  | D |  | N/A | D |  |
| BEHKA-HIV (Osborn 2010)^170^ |  |  | A | V |  |  |  | N/A |  |  |
| HTRM (Fernández 2011)^171^ |  |  | A | V |  | A |  | N/A |  |  |
| HTRFS (Claborn 2015)^172^ |  |  | I | V |  |  |  | N/A | D |  |
| HECCS (Logie 2017)^173^ |  |  | A | V |  |  |  | N/A | V |  |
| SSI (Renwick 1999)^174^ | I |  | A | V |  |  |  | N/A | I |  |
| USII-HIV (Ingram 1999)^175^ |  |  | A | V |  |  |  | N/A | D |  |
| PSS-HIV (Cortes 2014)^176^ |  |  |  | V |  |  |  | N/A | D |  |
| HIV-ASES (Johnson 2007)^177^ |  |  | V | V |  | D |  | N/A | D |  |
| PHIVSMS (Wallston 2011)^178^ |  |  |  | V |  |  |  | N/A | D |  |
| HIV-SMS-W (Webel 2012)^179^ | I |  | V | V |  | I |  | N/A |  |  |
| HIV-IM (Nelsen 2012)^180^ | D |  | V | V |  |  |  | N/A | D |  |
| HIVESS (Gray 2016)^181^ |  |  | V | V |  |  |  | N/A | I |  |
| HSM-SEWS (Webel 2011)^182^ |  |  | A | V |  | I |  | N/A | I |  |

Abbreviations: PROM, Patient Reported Outcome Measure; AIDS, Acquired Immune Deficiency Syndrome; HIV, Human Immunodeficiency Virus; ART, antiretroviral therapy; PLWHA, people living with HIV/AIDS; CCV/MI, Cross-cultural validity/ measurement invariance; HTCV: Hypotheses testing for construct validity; MOS-HIV, Medical Outcomes Study-HIV Health Survey; HOPES, HIV Overview of Problems Evaluation System; HIV-QoL, HIV-related Quality of Life Questions; AIDS-HAQ, AIDS Health Assessment Questionnaire; HIV-PARSE, HIV Patient Reported Status and Experience; HRQOL, Health-Related Quality of Life; FAHI, Functional Assessment of HIV Infection; GHSA, General Health Self-Assessment; HIV-QL31, HIV Quality of Life 31 items Scale; HAT-QoL, HIV/AIDS Targeted QOL Instrument; MQoL-HIV, Multidimensional QOL for Patients with HIV/AIDS; LWHIVS, Living with HIV Scale; WHOQOL-HIV, World Health Organization Quality of Life HIV; ISSQoL, Instituto Superiore di Sanità Quality of Life; HIV-SQUAD, Symptom Quality of Life Adherence; PROQOL-HIV, Patient Reported Outcome Quality of Life-HIV Questionnaire; PozQol, Poz Quality of Life; RSC, Riverside Symptom Checklist; HSI, HIV Symptom Index; HAT, HIV Assessment Tool; SSC-HIV, Sign and Symptom Checklist for HIV; HCSUS-SM, HIV Cost and Services Utilization Study Symptom Measure; HIV-SI/SDM, HIV Symptom Index or Symptoms Distress Module of the ACTG; HRFS, HIV-Related Fatigue Scale; HDQ, HIV Disability Questionnaire; ISS-HIV-SS, Istituto Superiore di Sanità-HIV symptoms scale; HSS-40, HIV Stigma Scale; HASI-P, HIV/AIDS Stigma Instrument-PLWA; IHSM, Internalized HIV Stigma measure; IA-RSS, Internalized AIDS-Related Stigma Scale; ISAT, Internalized Stigma in those with HIV/AIDS; HARSI, HIV and Abuse Related Shame Inventory; SEP-HASS, Self, experienced, and perceived HIV/AIDS stigma scales; HIV-SM, HIV stigma mechanisms; HA-SAL-GBT, HIV/AIDS stigma assessment for Latino gay men, bisexual men and transgender women living with HIV; VR-HARSSR, Van Rie HIV/AIDS-Related Stigma Scale -Revised for use in the USA; MAH, The Mental Adjustment to HIV scale; SS-HIV, HIV/AIDS Stress Scale; PSSHIV, Perceived Stress Scale among PLWHA; ISCS, Impact on Self-Concept Scale; IHIV, Impact of HIV; HIVMS, HIV Meaningfulness Scale; PLHIV-RS, People Living with HIV Resilience Scale; BIS, Body Image in Patients with HIV/AIDS; OCLS, Owen Clinic Lipodystrophy Scale; ACTG-ABCD, Adult AIDS Clinical Trial Group's Assessment of Body Change and Distress; ACTG-ABCD Short form, ACTG-ABCD-SF; FAI, Facial Appearance Inventory; MAS, Medication Attribution Scale; HIVTSQ, HIV Treatment Satisfaction Questionnaire; TES, Treatment-related Empowerment Scale; SIS, Subcutaneous Injection Survey; QUOTE-HIV, Quality of care through the patient’s eyes; AHHCP, Attitudes toward HIV Health Care Provider scale; AGAS, Antiretroviral General Adherence Scale; HCR, Health Care Relationship Trust Scale; HMRS, HIV Medication Readiness Scale; HTOS, HIV Treatment Optimism Scale; HIV-MT-SES, HIV Medication Taking Self-Efficacy Scale; BEHKA-HIV, Brief Estimate of Health Knowledge and Action-HIV version; HTRM, HIV Treatment Readiness Measure; HTRFS, HIV Treatment Regimen Fatigue Scale; HECCS, HIV Engagement in and Continuity of Care Scale; SSI, Social Support Inventory; USII-HIV, Unsupportive Social Interactions Inventory –HIV version; PSS-HIV, Perceived Social Support for HIV; HIV-ASES, HIV Treatment Adherence Self-Efficacy Scale; PHIVSMS, Perceived HIV Self-Management Scale; HIV-SMS-W, HIV Self-management Scale (Women); HIV-IM, HIV Intention Measure; HIVESS, HIV Exercise Stereotypes Scale; HSM-SEWS, HIV Symptom Management Self-Efficacy for Women Scale.

Methodological quality rated as: “A” =Adequate; “D” =Doubtful; “I” =Inadequate; “V” =Very good; “N/A”: Not applicable; “empty boxes” = not reported.

## Table S6. Results and Ratings for Each Psychometric Property of Each Record

| **PROM (Author Year)** | **Structural validity** | | **Internal consistency** | | **CCV/MI** | | **Reliability** | | **Measurement error** | | **HTCV** | | **Responsiveness** | |
| --- | --- | --- | --- | --- | --- | --- | --- | --- | --- | --- | --- | --- | --- | --- |
|  | **N** | **Results (Rating)** | **N** | **Results (Rating)** | **N** | **Results (Rating)** | **N** | **Results (Rating)** | **N** | **Results (Rating)** | **N** | **Results (Rating)** | **N** | **Results (Rating)** |
| MOS-HIV (Carretero 1996)^31^ |  |  | 100 | Subscale Cronbach's alpha =0.67 - 0.92. (-) |  |  |  |  |  |  | 100 | Results in line with 5 hypo (5+). |  |  |
| MOS-HIV (Murri 1997)^32^ |  |  | 213 | Subscale Cronbach's alpha =0.76 - 0.93. (+) |  |  |  |  |  |  | 213 | Result in line with 1 hypo (1+); Result not in line with 1 hypo’s (1‐). |  |  |
| MOS-HIV (Revicki 1998)^33^ | 1020 | 2 factors accounted for 62% of the variance, GFI =0.991. (+) | Group 1: 1022  Group 2: 1231  Group 3: 134 | Group 1: PHS Cronbach's alpha =0.91, MHS Cronbach's alpha =0.94; Group 2: PHS Cronbach's alpha =0.92, MHS Cronbach's alpha =0.94; Observational study: PHS Cronbach's alpha =0.90, MHS Cronbach's alpha =0.90. (+) |  |  | 134 | PHS ICC =0.72; MHS ICC =0.53. (-) |  |  | Group 1: 1022  Group 2: 1231  Group 3: 134 | Group1: Results in line with 5 hypo (5+); Group2: Result in line with 1 hypo (1+); Group 3: Results in line with 2 hypo (2+). |  |  |
| MOS-HIV (Badia 1999)^34^ |  |  | 275 | Subscale Cronbach's alpha =0.78 - 0.89. (+) |  |  | 48 | Subscale ICC =0.3-0.85. (-) |  |  | 275 | Results in line with 3 hypo (3+); Results not in line with 3 hypo’s (3‐). |  |  |
| MOS-HIV (Holmes 1999)^35^ |  |  | 215 | Subscale Cronbach's alpha =0.80 - 0.93. (+) |  |  |  |  |  |  | 215 | Results in line with 10 hypo (10+). |  |  |
| MOS-HIV (Scott-Lennox 1999)^36^ | 363 | 2 factors in the combined sample and for all translations except German as evidenced by the number of eigenvalues greater than 1.0 and the overall pattern of factor loadings. German's covariance of the scale scores could be summarized in a single dimension. (?) | 363 | Subscale Cronbach's alpha exceeded 70 for all translations except for the Cronbach's alpha of 0.68 for the Mental Health subscale in the French sample. (-) |  |  |  |  |  |  | 363 | Result in line with 1 hypo (1+); Result not in line with 1 hypo’s (1‐). |  |  |
| MOS-HIV (Delate 2001)^37^ |  |  | 242 | Subscale Cronbach's alpha =0.80 - 0.92. (+) |  |  |  |  |  |  | 242 | Results in line with 2 hypo (2+); Results not in line with 2 hypo’s (2‐). |  |  |
| MOS-HIV (Paton 2002)^38^ | 163 | 2 principal components with eigen values >1 which together accounted for 57.2%of the variance. (?) | 163 | Subscale Cronbach's alpha =0.73 - 0.95. (+) |  |  |  |  |  |  | 163 | Results in line with 6 hypo (6+). |  |  |
| MOS-HIV (Schifano 2003)^39^ |  |  | 146 | Subscale Cronbach's alpha =0.8 - 0.9. (+) |  |  |  |  |  |  | 146 | Results in line with 2 hypo (2+); Result not in line with 1 hypo’s (1‐). |  |  |
| MOS-HIV (Ichikawa 2004)^40^ | 200 | 2 factors explained 56% of the total variance. (?) | 200 | Subscale Cronbach's alpha =0.74 - 0.88. (+) |  |  |  |  |  |  | 200 | Results in line with 2 hypo (2+). |  |  |
| MOS-HIV (Lau 2006)^41^ | 242 | 2 factors explained 63.04% of the total variance. (?) | 242 | Subscale Cronbach's alpha =0.78 - 0.90. (+) |  |  |  |  |  |  | 242 | Results in line with 6 hypo (6+); Results not in line with 4 hypo (4-). |  |  |
| MOS-HIV (Taylor 2009)^42^ | 400 | MOS-HIV subscales resulted in one rather than two factors. (?) | 400 | Subscale Cronbach's alpha =0.54 - 0.80. (-) |  |  |  |  |  |  | 400 | Results in line with 7 hypo (7+). |  |  |
| MOS-HIV (Henderson 2010)^43^ | 532 | HIV+LD: CFI =0.991, RMSEA =0.064, SRMR =0.022; HIV: CFI =0.990，RMSEA =0.081，SRMR =0.020. (+) | 532 | HIV: Cronbach's alpha =0.970; HIV+LD: Cronbach's alpha =0.965. (+) |  |  |  |  |  |  | 532 | Result in line with 1 hypo (1+). |  |  |
| MOS-HIV (Stasinopoulou 2010)^44^ |  |  | 154 | Subscale Cronbach's alpha =0.70 - 0.88. (+) |  |  |  |  |  |  | 154 | Results in line with 3 hypo (3+); Result not in line with 1 hypo (1-). |  |  |
| MOS-HIV (Chariyalertsak 2011)^45^ |  |  | 50 | PHS Cronbach's alpha =0.76; MHS Cronbach's alpha =0.76; Subscale Cronbach's alpha =0.67 - 0.78. (-) |  |  |  |  |  |  | 50 | Results in line with 5 hypo (5+); Results not in line with 3 hypo (3-). |  |  |
| MOS-HIV (Hsiung 2011)^46^ | 619 | NNFI =0.92, CFI =0.94, SRMR =0.056. (+) | 619 | Subscale Cronbach's alpha =0.54 - 0.95. (-) |  |  |  |  |  |  | 619 | Results in line with 7 hypo (7+); Results not in line with 3 hypo (3-). |  |  |
| MOS-HIV (Epino 2012)^47^ | 610 | One primary health factor accounted for 57% of total variance. (?) | 610 | Subscale Cronbach's alpha =0.80 - 0.90. (+) |  |  |  |  |  |  | 610 | Results in line with 4 hypo (4+); Result not in line with 1 hypo (1-). |  |  |
| MOS-HIV (Huang 2012)^48^ |  |  | 120 | Subscale Cronbach's alpha =0.75 - 0.91. (+) |  |  |  |  |  |  | 120 | Results in line with 2 hypo (2+); Result not in line with 1 hypo (1-). |  |  |
| MOS-HIV (Marzieh 2017)^49^ | 100 | RMSEA =0.06, SRMR =0.07, NNFI =0.96, CFI =0.97, GFI =0.92, AGFI =0.86. (+) | 100 | Subscale Cronbach's alpha =0.61 - 0.86. (-) |  |  |  |  |  |  |  |  |  |  |
| MOS-HIV (Liu 2018)^50^ | 635 | RMSEA =0.04, SRMR =0.061, NNFI =0.91, CFI =0.97, AGFI =0.93. (+) | 635 | PHS Cronbach's alpha =0.87; MHS Cronbach's alpha =0.89; subscale Cronbach's alpha =0.79 - 0.93. (+) |  |  | 60 | Subscale ICC =0.71 - 0.90. (+) |  |  | 635 | Results in line with 3 hypo (3+); Result not in line with 1 hypo’s (1‐). |  |  |
| MOS-HIV (Shim 2018)^51^ | 201 | Two-factor structures: RMSEA =0.27, CFI =0.608, TLI =0.449, SRMR =1.367; Modification structures: RMSEA =0.079, CFI =0.974, TLI =0.953, SRMR =0.027. (+) | 201 | PHS Cronbach’s alpha =0.90; MHS Cronbach’s alpha =0.94; Subscale Cronbach’s alpha =0.78 - 0.95. (+) |  |  |  |  |  |  | 201 | Results in line with 9 hypo (9+); Results not in line with 8 hypo’s (8‐). |  |  |
| MOS-HIV-29 (Stangl 2012)^52^ | 947 | 2 factors accounted for 64% of total variance. (?) | 947 | PHS Cronbach’s alpha=0.79, MHS Cronbach’s alpha=0.85, Subscale Cronbach’s alpha =0.79 - 0.92. (+) |  |  |  |  |  |  | 947 | Results in line with 10 hypo (10+). | 947 | Result in line with 1 hypo (1+). |
| MOS-HIV-17 (McDonnell 2000)^53^ |  |  | 287 | Subscale Cronbach’s alpha =0.64 - 0.89. (-) |  |  |  |  |  |  | 287 | Results in line with 2 hypo (2+);  Results not in line with 5 hypo’s (5‐). |  |  |
| HOPES (Schag 1992)^54^ |  |  | 318 | Subscale Cronbach’s alpha =0.55 - 0.95. (-) |  |  |  |  |  |  | 318 | Results in line with 6 hypo (6+). |  |  |
| HOPES (De Boer 1996)^55^ |  |  | 106 | Subscale Cronbach’s alpha =0.80 - 0.93. (+) |  |  |  |  |  |  | 106 | Result in line with 1 hypo (1+);  Result not in line with 1 hypo (1-). | 65 | Result in line with 1 hypo (1+). |
| HIV-QoL (Cleary 1993)^56^ |  |  | 189 | Subscale Cronbach’s alpha =0.58 - 0.95. (-) |  |  |  |  |  |  |  |  |  |  |
| AIDS-HAQ (Lubeck 1997)^57^ |  |  | 1083 | Subscale Cronbach’s alpha =0.79-0.89. (+) |  |  |  |  |  |  | 1083 | Result in line with 1 hypo (1+). | 297 | Result in line with 1 hypo (1+). |
| HIV-PARSE (Bozzette 1994)^58^ |  |  | 1862 | Scale Cronbach’s alpha =0.94; Subscale Cronbach’s alpha =0.86 - 0.90. (+) |  |  |  |  |  |  |  |  |  |  |
| HIV-PARSE-Brief (Bozzette 1995)^59^ |  |  | 1934 | Subscale Cronbach’s alpha =0.78 - 0.93. (+) |  |  |  |  |  |  |  |  |  |  |
| HRQOL (Hays 1995)^60^ | 205 | CFI=0.91 (-). | 205 | Subscale Cronbach’s alpha =0.49 - 0.86. (-) |  |  |  |  |  |  | 205 | Results in line with 5 hypo (5+). |  |  |
| FAHI (Cella 1996)^61^ |  |  | SMS: 109;  BESS: 71;  BSSS: 64. | SMS: Scale Cronbach’s alpha =0.91; Subscale Cronbach’s alpha =0.55 - 0.89. (-); BESS: Scale Cronbach’s alpha =0.91; Subscale Cronbach’s alpha =0.63 - 0.89. (-);  BSSS: Scale Cronbach’s alpha =0.92; Subscale Cronbach’s alpha =0.60 - 0.91. (+). |  |  |  |  |  |  | SMS: 67;  BESS: 70;  BSSS: 64. | SMS: Results in line with 7 hypo (7+), Result not in line with 1 hypo (1-); BESS: Result in line with 1 hypo (1+); BSSS: Result in line with 1 hypo (1+). |  |  |
| FAHI (Peterman 1997)^62^ | 361 | 3 items had fit statistics outside the acceptable range on the physical well-being subscale; 3 items on the function and global well-being subscale with fit statistics outside the suggested range; 1 item on the emotional wellbeing/living with HIV subscale with fit statistics outside the suggested range. (-) | 361 | Subscale Cronbach’s alpha =0.73 - 0.91. (+) |  |  |  |  |  |  | 361 | Results in line with 5 hypo (5+). |  |  |
| FAHI (Viala-Danten 2010)^63^ |  |  | Group1: 565; Group2: 1096. | 563: Subscale Cronbach’s alpha=0.75-0.91. (+)  1091: Subscale Cronbach’s alpha=0.72-0.92. (+) |  |  |  |  |  |  | Group1: 565; Group2: 1096. | Results in line with 2 hypo (2+). | Group1: 565; Group2: 1096. | Result in line with 1 hypo (1+). |
| FAHI (Byrne 2013)^64^ |  |  | 170 | Scale Cronbach’s alpha =0.92; Subscale Cronbach’s alpha =0.70 - 0.89. (+) |  |  |  |  |  |  | 170 | Results in line with 4 hypo (4+);  Results not in line with 2 hypo (2-). |  |  |
| FAHI (Nyongesa 2017)^65^ | 103 | 5 factors, 52.2% of the variance being explained. (?) | 103 | Subscale Cronbach’s alpha =0.55 - 0.78. (-) |  |  |  |  |  |  | 103 | Result in line with 1 hypo (1+). |  |  |
| GHSA (Lenderking 1997)^66^ | 1694 | Five QoL modules could be reduced to a psychological and a physical factor. (?) | 1694 | Subscale Cronbach’s alpha=0.80-0.93. (+) |  |  |  |  |  |  | 1694 | Results in line with 6 hypo (6+);  Result not in line with 1 hypo (1-). |  |  |
| HIV-QL31 (Leplège 1997)^67^ | 102 | The 31 items have infit statistics that satisfy this criterion. If between 0.7 and 1.3 is adopted, the number of items to be withdrawn is nine out of 31. (-) | 102 | Scale Cronbach’s alpha =0.93; Subscale Cronbach’s alpha > 0.84. (+) |  |  |  |  |  |  | 102 | Results in line with 2 hypo (2+); Result not in line with 1 hypo (1-). |  |  |
| HAT-QoL-42 (Holmes 1997)^68^ |  |  | 106 | Subscale Cronbach’s alpha =0.48 - 0.90. (-) |  |  |  |  |  |  | 106 | Results in line with 7 hypo (7+);  Results not in line with 4 hypo’s (4‐). |  |  |
| HAT-QoL-42 (Holmes 1998)^69^ |  |  | 201 | Subscale Cronbach’s alpha =0.54 - 0.90. (-) |  |  |  |  |  |  | 201 | Results in line with 9 hypo (9+); Results not in line with 5 hypo’s (5‐). |  |  |
| HAT-QoL-30 (Holmes 1999)^35^ |  |  | 215 | Subscale Cronbach’s alpha =0.57 - 0.90. (-) |  |  |  |  |  |  | 215 | Results in line with 10 hypo (10+). |  |  |
| HAT-QoL-34 (Holmes 2008)^70^ |  |  | 153 | Subscale Cronbach’s alpha =0.80 - 0.92. (+) |  |  | 60 | Subscale ICC =0.64 - 0.84. (-) |  |  |  |  |  |  |
| HAT-QoL-34 (Taylor 2009)^42^ | 400 | Seven-factor solution explaining 61% of the total variance. (?) | 400 | Subscale Cronbach’s alpha =0.63 - 0.85. (-) |  |  |  |  |  |  | 400 | Results in line with 7 hypo (7+). |  |  |
| HAT-Qol-34 (Mafirakureva 2016)^71^ |  |  | 257 | Scale Cronbach’s alpha =0.83; Subscale Cronbach’s alpha =0.79 - 0.84. (+) |  |  |  |  |  |  | 257 | Results in line with 2 hypo (2+). |  |  |
| MQoL-HIV (Smith 1997)^72^ |  |  | 121 | Subscale Cronbach’s alpha =0.56 - 0.86. (-) |  |  | 121 | Subscale Cronbach’s alpha =0.64 - 0.88. (-) |  |  | 121 | Results in line with 2 hypo (2+). | 95 | Result in line with 1 hypo (1+). |
| MQoL-HIV (Badia 1999)^34^ |  |  | 283 | Scale Cronbach’s alpha =0.89; Subscale Cronbach’s alpha =0.44 - 0.82. (-) |  |  | 50 | Scale ICC =0.54; Subscale ICC =0.48 - 0.82. (-) |  |  | 283 | Results in line with 3 hypo (3+); Results not in line with 3 hypo’s (3‐). |  |  |
| MQoL-HIV (Kemmler 2003)^73^ |  |  | 207 | Subscale Cronbach’s alpha =0.61 - 0.85. (-) |  |  | 109 | Scale Pearson’s r =0.86; Subscale Pearson’s r =0.67 - 0.89. (-) |  |  | 207 | Results in line with 12 hypo (12+);  Results not in line with 3 hypo (3-). |  |  |
| MQoL-HIV (Remple 2004)^74^ |  |  | 85 | Subscale Cronbach’s alpha =0.43 - 0.92. (-) |  |  | 43 | Scale Pearson’s r =0.68; Subscale Pearson’s r =0.60 - 0.96. (-) |  |  | 85 | Result in line with 1 hypo (1+). |  |  |
| MQoL-HIV (Watanabe 2004)^75^ | 375 | Eight factors accounted for 76.9% of the total variance. (?) | 375 | Subscale Cronbach’s alpha =0.47-0.85. (-) |  |  |  |  |  |  | 375 | Results in line with 4 hypo (4+). |  |  |
| LWHIVS (Holzemer 1998)^76^ | 187 | 2 factors, 49.5％ of the variance being explained. (?) | 187 | Scale Cronbach’s alpha =0.84; Factor 1 Cronbach’s alpha =0.70; Factor 2 Cronbach’s alpha =0.41; Subscale Cronbach’s alpha =0.37 - 0.81. (-) |  |  |  |  |  |  | 187 | Results in line with 2 hypo (2+); Results not in line with 2 hypo (2-). |  |  |
| WHOQOL-HIV (Starace 2002)^77^ |  |  | 151 | Facet Cronbach’s alpha =0.53-0.89 (-) |  |  |  |  |  |  | 151 | Result in line with 1 hypo (1+);  Results not in line with 2 hypo (2-). |  |  |
| WHOQOL-HIV (WHOQOL HIV Group 2004)^78^ | 1334 | CFI=0.97. (+) | 1334 | All facet Cronbach’s alpha >0.7, apart from Safety and Security Cronbach’s alpha =0.67. (-) |  |  |  |  |  |  |  |  |  |  |
| WHOQOL-HIV (Zimpel 2007)^79^ |  |  | 308 | Facet Cronbach’s alpha = 0.32 - 0.92 (-). |  |  |  |  |  |  | 308 | Results in line with 8 hypo (8+). |  |  |
| WHOQOL-HIV (Canavarro 2011)^80^ |  |  | 200 | scale Cronbach’s alpha =0.86; Subscale Cronbach’s alpha =0.86-0.95 (+). |  |  |  |  |  |  | 200 | Results in line with 3 hypo (3+). |  |  |
| WHOQOL-HIV (Mweemba 2011)^81^ | 160 | 7 items be eliminated, 8 factors. (?) | 160 | Subscale Cronbach’s alpha =0.829 - 0.933 (+). |  |  |  |  |  |  |  |  |  |  |
| WHOQOL-HIV (Olsen 2013)^82^ |  |  |  |  | 20 | The conceptual equivalence of WHOQOL-HIV is only partially attained for use in Ethiopia. (?) |  |  |  |  |  |  |  |  |
| WHOQOL-HIV (Reychler 2013)^83^ |  |  | 50 | Scale Cronbach’s alpha =0.938; Subscale Cronbach’s alpha =0.937 - 0.9344 (+). |  |  | 50 | Scale ICC =0.781; Subscale ICC =0.798 - 0.931. (+) |  |  | 50 | Results in line with 10 hypo (10+). |  |  |
| WHOQOL-HIV-BREF (Saddki 2009)^84^ | 157 | 5 factors explained 60.1% of total variance. (?) | 157 | Subscale Cronbach’s alpha =0.70 - 0.83. (+) |  |  | 51 | Subscale ICC = 0.60 - 0.87. (-) |  |  | 157 | Result in line with 1 hypo (1+). |  |  |
| WHOQOL-HIV-BREF (Hsiung 2011)^85^ | 680 | NNFI =0.95, CFI =0.95, RMSEA =0.082, SRMR =0.091. (+) | 680 | Scale Cronbach’s alpha = 0.93; Subscale Cronbach’s alpha = 0.67 - 0.80. (-) |  |  |  |  |  |  | 680 | Results in line with 5 hypo (5+); Result not in line with 1 hypo (1-). |  |  |
| WHOQOL-HIV-BREF (O'Connell 2012)^86^ | 1923 | CFI =0.97, RMSEA =0.17. (+) | 1923 | Subscale Cronbach’s alpha =0.69 - 0.82. (-) |  |  |  |  |  |  | 1923 | Result in line with 1 hypo (1+). |  |  |
| WHOQOL-HIV-BREF (Tran 2012)^87^ | 1016 | 6 factors accounted for 60.0% of total variance. (?) | 1016 | Subscale Cronbach’s alpha =0.67 - 0.89. (-) |  |  |  |  |  |  | 1016 | Result in line with 1 hypo (1+); Result not in line with 1 hypo (1-). |  |  |
| WHOQOL-HIV-BREF (Canavarro 2012)^88^ | 1196 | CFI =0.81, RMSEA =0.078. (-) | 1196 | Scale Cronbach’s alpha=0.93, Subscale Cronbach’s alpha =0.61 - 0.80. (-) |  |  |  |  |  |  | 1196 | Result in line with 1 hypo (1+). |  |  |
| WHOQOL-HIV-BREF (Pereira 2014)^89^ | 185 | CFI =0.97, RMSEA =0.07. (+) | 185 | Scale Cronbach’s alpha =0.77, Subscale Cronbach’s alpha =0.65 - 0.86. (-) |  |  |  |  |  |  | 185 | Results in line with 2 hypo (2+); Result not in line with 1 hypo (1-). |  |  |
| WHOQOL-HIV-BREF (Meemon 2016)^90^ | 329 | CFI =0.93, SRMR =0.52, RMSEA =0.15. (-) | 329 | Scale Cronbach’s alpha=0.91, Subscale Cronbach’s alpha =0.45 - 0.80. (-) |  |  |  |  |  |  | 329 | Results not in line with 2 hypo (2-). |  |  |
| WHOQOL-HIV-BREF (Salehi 2016)^91^ |  |  | 61 | Scale Cronbach’s alpha =0.87, Subscale Cronbach’s alpha =0.71 - 0.83. (+) |  |  |  |  |  |  |  |  |  |  |
| WHOQOL-HIV-BREF (Tesfaye 2016)^92^ | 348 | CFI =0.82, TLI =0.77, RMSEA =0.06. (-) | 348 | Scale Cronbach’s alpha =0.92, Subscale Cronbach’s alpha =0.53 - 0.84. (-) |  |  |  |  |  |  | 348 | Results in line with 6 hypo (6+). |  |  |
| WHOQOL-HIV-BREF (Zhu 2017)^93^ | 1100 | 6 domains: CFI =0.81, RMSEA =0.07, AGFI =0.87. (-) | 1100 | Scale Cronbach’s alpha =0.93, Subscale Cronbach’s alpha =0.66 - 0.85. (-) |  |  | 57 | Subscale ICC =0.72 - 0.82 (+). |  |  | 1100 | Results in line with 3 hypo (3+). |  |  |
| WHOQOL-HIV-BREF (Fuster-Ruizde Apodaca 2019)^94^ | 1462 | 6 domains: CFI =0.97, GFI =0.98, AGFI =0.97, NFI =0.97, RMSEA =0.073, SRMR =0.064. (+)  5 domains: CFI =0.97, GFI =0.98, AGFI =0.98, NFI =0.97, RMSEA =0.066, SRMR =0.058. (+) | 1462 | Scale Cronbach’s alpha =0.77, Subscale Cronbach’s alpha =0.61 - 0.81. Subscale Omega coefficient =0.623 - 0.851. (-) |  |  |  |  |  |  | 1462 | Results in line with 4 hypo (4+); Result not in line with 1 hypo’s (1‐). |  |  |
| WHOQOL-HIV-BREF (Barger 2020)^95^ | 586 | SRMR =0.059; CFI =0.834; RMSEA =0.070. (+) | 586 | Scale Cronbach’s alpha =0.76; Subscale Cronbach’s alpha =0.63 - 0.79. (-) |  |  |  |  |  |  | 586 | Results in line with 2 hypo (2+) |  |  |
| ISSQoL (Bucciardini 2006)^96^ |  |  | 322 | Subscale Cronbach’s alpha =0.70 - 0.93. (+) |  |  |  |  |  |  | 322 | Results in line with 2 hypo (2+). |  |  |
| HIV-SQUAD (Spire 2009)^97^ | 270 | 44% of variance explained by the ﬁrst factor. (?) | 282 | Subscale Cronbach’s alpha =0.60 - 0.84. (-) |  |  |  |  |  |  | 293 | Results in line with 4 hypo (4+). |  |  |
| PROQOL-HIV-43 (Duracinsky 2012(1))^98^ |  |  |  |  |  |  |  |  |  |  |  |  |  |  |
| PROQOL-HIV-43 (Duracinsky 2012(2))^99^ | 791 | 8 factors accounted for 60% of total variance. (?) | 791 | Subscale Cronbach’s alpha =0.686 - 0.885. (-) |  |  | 34 | Scale ICC =0.859. (+) |  |  | 791 | Results in line with 6 hypo (6+). |  |  |
| PROQOL-HIV-43 (Duracinsky 2014)^100^ |  |  |  |  |  |  | 59 | Scale ICC =0.868; Subscale ICC =0.708 - 0.939. (+) |  |  |  |  |  |  |
| PROQOL-HIV-38 (Lalanne 2016)^101^ | 2526 | CFI =0.941, RMSEA =0.063, WRMR =2.836. (-) | 1693 | Subscale Cronbach’s alpha =0.791 - 0.907. (+) |  |  |  |  |  |  | 1693 | Results in line with 3 hypo (3+). |  |  |
| PozQol (Brown 2018)^102^ | 465 | CFI =0.992, SRMR =0.036, RMSEA =0.034. (+) | 465 | Scale Cronbach’s alpha =0.95; Subscale Cronbach’s alpha =0.82 - 0.91. (+) |  |  | 51 | Scale ICC =0.95; Subscale ICC =0.83 - 0.91 (+). |  |  | 465 | Results in line with 8 hypo (8+). |  |  |
| RSC (Burgess1993)^103^ |  |  | 103 | Physical Cronbach’s alpha =0.88; Cognitive Cronbach’s alpha =0.90; Psychological Cronbach’s alpha =0.83; Total Cronbach’s alpha =0.93. (+) |  |  |  |  |  |  | 103 | Results in line with 4 hypo (4+). |  |  |
| HSI (Whalen 1994)^104^ |  |  | 148 | Scale Cronbach’s alpha =0.79. (+) |  |  | 21 | Scale ICC =0.92. (+) |  |  | 148 | Results in line with 2 hypo (2+); Results not in line with 4 hypo (4-). | 60 | Result in line with 1 hypo (1+). |
| HAT (Nokes 1994)^105^ | 156 | The first three factors had eigenvalues greater than 2.4 and together explained 45.4% of the variance. (?) | 156 | Scale Cronbach’s alpha =0.92. (+) |  |  | 156 | Scale Cronbach’s alpha =0.96. (+) |  |  |  |  |  |  |
| SSC-HIV (Holzemer 1999)^107^ | 686 | Six factors explained 68.94% of the variance. (?) | 686 | Scale Cronbach’s alpha =0.95; Subscale Cronbach’s alpha =0.77 - 0.90. (+) |  |  |  |  |  |  |  |  |  |  |
| SSC-HIV (Sousa 2006)^106^ | 917 | RMSEA =0.036, SRMR =0.061, CFI =0.99. (+) |  |  |  |  |  |  |  |  |  |  |  |  |
| SSC-HIV-rev (Holzemer 2001)^108^ | 372 | 11-factor explained 73.3% of the variance. (?) | 372 | Scale Cronbach’s alpha=0.97; Subscale Cronbach’s alpha =0.76 - 0.91. (+)  Subscale GYN Problems Cronbach’s alpha =0.94. (+) |  |  |  |  |  |  | 372 | Results in line with 4 hypo (4+); Results not in line with 5 hypo (5-). |  |  |
| HCSUS-SM (Mathews 2000)^109^ |  |  | 3072 | Scale Cronbach’s alpha =0.80. (+) |  |  |  |  |  |  |  |  |  |  |
| HIV-SI/SDM (Justice 2001)^110^ |  |  |  |  |  |  |  |  |  |  | 113 | Results in line with 6 hypo (6+) |  |  |
| HIV-SI/SDM (Regnault 2009)^111^ |  |  |  |  | 759 | Statistically significant DIF between cultural groups was observed for four items: fatigue; fevers; anxiety; and headache. (-) |  |  |  |  |  |  |  |  |
| HIV-SI/SDM (Marc 2012)^112^ | 5521 | Two factors accounted for 41.4% of the total variance. GFI=0.88, AGFI=0.85, NFI=0.99 and NNFI=0.99, RMSEA=0.06. (+) | 5521 | Scale Cronbach’s alpha =0.92. Subscale Cronbach’s alpha =0.81 - 0.90. (+) |  |  |  |  |  |  | 5521 | Results in line with 7 hypo (7+). |  |  |
| HRFS (Barroso 2002)^113^ |  |  | 54 | Scale Cronbach’s alpha =0.94; Subscale Cronbach’s alpha =0.73 - 0.95. (+) |  |  | 14 | Scale r =0.43. (-) |  |  |  |  |  |  |
| HRFS (Pence 2008)^114^ |  |  | 128 | Subscale Cronbach’s alpha =0.91 - 0.97. (+) Fatigue-related impairment of functioning Subscale Cronbach’s alpha =0.92 - 0.95. (+) |  |  |  |  |  |  | 128 | Results in line with 8 hypo (8+). |  |  |
| HRFS (Li 2016)^115^ |  |  | 142 | Scale Cronbach’s alpha =0.97; Subscale Cronbach’s alpha =0.84 - 0.94. (+) |  |  | 142 | Scale r =0.686; Subscale r = 0.337 - 0.738. (-) |  |  | 142 | Results in line with 2 hypo (2+). |  |  |
| HDQ (O'Brien 2014)^116^ | 361 | CFI =0.812, TLI =0.805, RMSEA =0.030. (+) |  |  |  |  |  |  |  |  |  |  |  |  |
| HDQ (O'Brien 2015)^117^ |  |  | 235 | HDQ severity: Scale Cronbach’s alpha =0.96, 0.97; Subscale Cronbach’s alpha =0.84 - 0.93  HDQ episodic: Scale Cronbach’s alpha =0.96, 0.98; Subscale Cronbach’s alpha =0.81 - 0.95 (+) |  |  | 99 | Scale ICC =0.90; Subscale ICC =0.80 - 0.89. (+) |  |  | 235 | Canada: Results in line with 34 hypo (34+); Results not in line with 8 hypo’s (8‐). Ireland: Results in line with 22 hypo (22+); Results not in line with 18 hypo’s (18‐) |  |  |
| HDQ (Brown 2019)^118^ |  |  | 243 | HDQ Severity Scale: Scale Cronbach’s alpha =0.96; Subscale Cronbach’s alpha =0.85 - 0.93 (+); HDQ Episodic Scale: Scale KR-20 =0.95; Subscale KR-20 =0.74 - 0.91 (+). |  |  |  |  | 243 | SDC of 7.29 (difficulties with day-to-day activities) to 15.05 (cognitive domain). (?) | 243 | Results in line with 30 hypo (30+); Results not in line with 6 hypo’s (6‐). |  |  |
| HDQ (O’Brien 2019)^119^ |  |  | 128 | Scale Cronbach’s alpha=0.97; Subscale Cronbach’s alpha =0.88 - 0.93. (+)  Scale KR-20 =0.98; Subscale KR-20 =0.86 - 0.96. (+) |  |  | 61 | Scale ICC =0.94; Subscale ICC =0.88 - 0.92. (+) |  |  | 124 | Results in line with 13 hypo (13+); Results not in line with 2 hypo (2-). |  |  |
| ISS-HIV-SS (Bucciardini 2016)^120^ | 161 | 5 factors, 64.96％ of the variance being explained. (?) | 161 | Subscale Cronbach’s alpha =0.70 - 0.91. (+) |  |  |  |  |  |  | 161 | Result in line with 1 hypo (1+). |  |  |
| HSS-40 (Berger 2001)^121^ | 318 | The variance explained by the four-factor solution was 46%. (?) | 318 | Scale Cronbach’s alpha=0.96; Subscale Cronbach’s alpha =0.90 - 0.93. (+) |  |  | 139 | Scale r =0.92; Subscale r =0.87 - 0.90 (+). |  |  | 318 | Results in line with 6 hypo (6+). |  |  |
| HSS-40 (Rao 2008)^122^ |  |  |  |  | 541 | IRT analysis revealed that eleven out of forty items functioned differently across groups. (-) |  |  |  |  |  |  |  |  |
| HSS-32 (Bunn 2007)^123^ | 157 | CFI =0.908; RMSEA =0.072. (-) | 157 | Scale Cronbach’s alpha=0.96; Subscale Cronbach’s alpha =0.90 - 0.95. (+) |  |  |  |  |  |  | 157 | Results in line with 4 hypo (4+). |  |  |
| HSS-32 (Reinius 2018)^124^ | 1036 | 4 subscales: CFI =0.998, TLI =0.998, RMSEA =0.050; CFI =0.991, TLI =0.997, RMSEA =0.076; CFI =0.997, TLI =0.995, RMSEA =0.059; CFI =0.997, TLI =0.995, RMSEA =0.047. (+) |  |  | 1036 | DIF within US cohort: No items were flagged for DIF within the US cohort; DIF across cohorts-gender: One item was found to have non-uniform DIF between men and women; DIF between cohorts: Nine items in the HIV Stigma Scale were flagged for either uniform, non-uniform, or both uniform and non-uniform DIF between persons living in Sweden, the US, and South India, respectively. (-) |  |  |  |  |  |  |  |  |
| HSS-12 (Rongkavilit 2010)^125^ | 70 | GFI =0.90, RMSEA =0.02. (+) | 70 | Scale Cronbach’s alpha =0.75; Subscale Cronbach’s alpha =0.77 - 0.93. (+) |  |  |  |  |  |  | 70 | Result in line with 1 hypo (1+); Results not in line with 4 hypo’s (4‐). |  |  |
| HSS-12 (Reinius 2017)^126^ | 880 | CFI =0.963, TLI =0.950, RMSEA =0.071. (+) | 880 | Subscale Cronbach’s alpha =0.80 - 0.88. (+) |  |  |  |  |  |  |  |  |  |  |
| HSS-12 (Luz 2020)^127^ | Grindr: 114; Social media: 164; Hornet: 1824 | Grindr + social media: CFI =0.995, TLI =0.993, RMSEA =0.033, SRMR =0.038; Hornet + social media: CFI =0.983, TLI =0.976, RMSEA =0.06, SRMR =0.034; Hornet: CFI =0.980, TLI =0.973, RMSEA =0.071, SRMR =0.039. (+) | Grindr: 114  Social media: 164  Hornet: 1824 | Scale Cronbach’s alpha =0.83; Subscale Cronbach’s alpha =0.69 - 0.87. (-)  Scale omega =0.93; Subscale omega =0.78 - 0.94. (+) | Grindr: 114; Social media: 164; Hornet: 1824 | Statistically significant DIF between 2 groups was observed for 1 item: 10th item. (-) |  |  |  |  | Grindr: 114; Social media: 164; Hornet: 1824 | Grindr: Results in line with 2 hypo (2+); Results not in line with 10 hypo (10-). Social media: Results in line with 2 hypo (2+); Results not in line with 10 hypo (10-). Hornet: Results in line with 7 hypo (7+); Results not in line with 5 hypo (5-). |  |  |
| HSS-39 (Lindberg 2014)^128^ | 194 | 4 factors accounted for 62.2% of total variance. (?) | 194 | Scale Cronbach’s alpha =0.958;  Subscale Cronbach’s alpha =0.871 - 0.958. (+) |  |  |  |  |  |  | 194 | Results in line with 2 hypo (2+). |  |  |
| HSS-30 (Fuster-RuizdeApodaca 2015)^129^ | 557 | CFI =0.98, GFI =0.96, AGFI =0.96, NFI =0.96, NNFI =0.98, IFI =0.98, RMR =0.073, RMSEA =0.051. (+) | 557 | Scale Cronbach’s alpha =0.88;  Second-order factors Cronbach’s alpha =0.82, 0.87;  First-order factors Cronbach’s alpha =0.70 - 0.89. (+) |  |  |  |  |  |  | 557 | Results in line with 3 hypo (3+). |  |  |
| HSS-10 (Kagiura 2020)^130^ | 451 | CFI =0.999, TLI =0.998, RMSEA =0.035, SRMR =0.037. (+) | 451 | Scale omega =0.90; Subscale omega =0.60 - 0.87. (-) |  |  |  |  |  |  | 451 | Results in line with 6 hypo (6+) |  |  |
| HASI-P (Holzemer 2007)^131^ | 1477 | The variance explained by the 6-factor solution was 60.72%. (?) | 1477 | Scale Cronbach’s alpha =0.940; Subscale Cronbach’s alpha =0.758 - 0.906. (+) |  |  |  |  |  |  | 1477 | Results in line with 3 hypo (3+). |  |  |
| HASI-P (Maluccio 2017)^132^ | 639 | 5 subscales. (?) | 639 | Scale Cronbach’s alpha =0.873; Subscale Cronbach’s alpha =0.740 - 0.848. (+) |  |  |  |  |  |  | 639 | Results in line with 2 hypo (2+). |  |  |
| IHSM (Sayles 2008)^133^ | 202 | Eigenvalues and the scree plot indicated four existing underlying factors. (?) | 202 | Scale Cronbach’s alpha =0.93; Subscale Cronbach’s alpha =0.66 - 0.91. (-) |  |  |  |  |  |  | 202 | Results in line with 10 hypo (10+); Results not in line with 7 hypo (7-). |  |  |
| IA-RSS (Kalichman 2009)^134^ |  |  | SA: 1068; Swaziland: 1087; USA: 219 | South Africa Scale Cronbach’s alpha =0.73; Swaziland Scale Cronbach’s alpha =0.74; USA Scale Cronbach’s alpha =0.76. (+) |  |  | SA: 71; USA: 110 | South Africa Scale r =0.45; USA Scale r =0.62. (-) |  |  | SA: 1068; Swaziland: 1087; USA: 219 | Results in line with 12 hypo (12+); Results not in line with 3 hypo (3-). |  |  |
| IA-RSS (Tsai 2013)^135^ | 456 | Only one factor. (?) | 456 | Scale Cronbach’s alpha =0.73. (+) |  |  | 456 | Scale ICC =0.4. (-) |  |  | 456 | Results in line with 4 hypo (4+); Result not in line with 1 hypo (1‐). |  |  |
| IA-RSS (Geibel 2020)^136^ | Cambodia: 1207; Dominican Republic: 891; Uganda: 391; Tanzania: 527 | Cambodia: CFI =0.998, TLI =0.996, RMSEA =0.021; Dominican Republic: CFI =0.997, TLI =0.993, RMSEA =0.024; Uganda: CFI =0.950, TLI =0.917, RMSEA =0.091; Tanzania: CFI =0.998, TLI =0.994, RMSEA =0.032. (+) | Cambodia: 1207; Dominican Republic: 891; Uganda: 391; Tanzania: 527 | Cambodia Scale KR-20 =0.80; Dominican Republic Scale KR-20 =0.71; Uganda Scale KR-20 =0.75; Tanzania Scale KR-20 =0.84. (+) |  |  |  |  |  |  | Cambodia: 1207; Dominican Republic: 891; Uganda: 391; Tanzania: 527 | Cambodia: Result in line with 1 hypo (1+); Results not in line with 2 hypo (2-). Dominican Republic: Results in line with 2 hypo (2+); Result not in line with 1 hypo (1-). Uganda: Results in line with 2 hypo (2+); Result not in line with 1 hypo (1-). Tanzania: Results in line with 3 hypo (3+); Result not in line with 1 hypo (1-). |  |  |
| ISAT (Phillips 2011)^137^ | 255 | Only one factor. (?) | 255 | Time 1, 2, 3 Scale Cronbach’s alpha =0.91, 0.92, 0.92. (+) |  |  |  |  |  |  | 255 | Results in line with 2 hypo (2+). |  |  |
| HARSI (Neufeld 2012)^138^ | 271 | 3-factors final model explained 56.7% of the sample variance. (?) | 271 | Subscale Cronbach’s alpha =0.77 - 0.93. (+) |  |  | 66 | Subscale ICC =0.37 - 0.83. (-) |  |  |  |  |  |  |
| SEP-HASS (Zelaya 2012)^139^ |  |  | 200 | Subscale Cronbach’s alpha =0.83 - 0.86. (+) |  |  |  |  |  |  | 200 | Result in line with 1 hypo (1+). |  |  |
| HIV-SM (Earnshaw 2013)^140^ |  |  | 95 | Subscale Cronbach’s alpha =0.87 – 0.89. (+) |  |  |  |  |  |  | 95 | Results in line with 7 hypo (7+); Results not in line with 14 hypo (14-). |  |  |
| HA-SAL-GBT (Molina 2013)^141^ | 170 | Removed two items with factor loadings less than 0.60, revealed three dimensions of stigma. (?) | 170 | Subscale Cronbach’s alpha =0.88 – 0.96. (+) |  |  |  |  |  |  | 170 | Results in line with 24 hypo (24+); Results not in line with 12 hypo (12-). |  |  |
| VR-HARSSR (Kipp 2015)^142^ | 85 | 3 factors accounted for 57% of the total variance. (?) | 85 | Scale Cronbach’s alpha =0.90; Subscale Cronbach’s alpha =0.69 - 0.90. (-) |  |  |  |  |  |  | 85 | Results in line with 3 hypo (3+); Result not in line with 1 hypo (1-). |  |  |
| MAHIVS (Ross 1994)^143^ | 107 | Five factors accounting for 49.7%. (?) | 107 | Subscale Cronbach’s alpha =0.55 - 0.80. (-) |  |  |  |  |  |  |  |  |  |  |
| SS-HIV (Niu 2016)^144^ | 667 | 17 items scale: CFI =0.923, GFI =0.924, TLI =0.909, IFI =0.923, RMSEA =0.067. (-) | 667 | Scale Cronbach’s alpha =0.906; Subscale Cronbach’s alpha =0.791 - 0.846. (+) |  |  | 667 | Scale ICC =0.908; Subscale ICC =0.766 - 0.829. (+) |  |  | 667 | Results in line with 3 hypo (3+). |  |  |
| PSSHIV (Su 2008)^145^ | 215 | 8 factors explaining 71.47% of the total variance, KMO=0.905. (?) | 215 | Scale Cronbach’s alpha =0.94; Subscale Cronbach’s alpha =0.76 - 0.93. (+) |  |  | 31 | Scale ICC =0.89; Subscale ICC =0.72 - 0.92. (+) |  |  | 215 | Results in line with 3 hypo (3+). |  |  |
| Screenphiv (Remor 2012)^146^ | 84 | 6 subscales; RMSEA =0.017. (+) | 84 | 23 facets Cronbach’s alpha =0.53 - 0.95. (-) |  |  |  |  |  |  |  |  |  |  |
| Screenphiv (Remor 2016)^147^ | 744 | CFI =1, NFI =1, GFI =0.91, AGFI =0.92, RMSEA =0.079, RMR =0.081. (+) | 744 | Scale Cronbach’s alpha =0.92, 0.92; Subscale Cronbach’s alpha =0.65 - 0.91. (-) |  |  |  |  |  |  | 744 | Result in line with 1 hypo (1+). |  |  |
| ISCS (Golub 2013)^148^ | 129 | 7 items loaded onto “self-loss” which accounted for 42% of the total variance. 3 items loaded onto “self-growth” which accounted for an additional 21% of the total variance. (?) | 129 | “self-loss” Cronbach’s alpha =0.88; “self-growth” Cronbach’s alpha =0.82. (+) |  |  |  |  |  |  | 129 | Results in line with 3 hypo (3+); Result not in line with 1 hypo (1-). |  |  |
| IHIV (Buscher 2015)^149^ | 356 | Four-factor Positive Impact model: NNFI =0.99, CFI =0.99, RMSEA =0.045, SRMR =0.055(+); Three-factor Negative Impact model: NNFI =0.97, CFI =0.98, RMSEA =0.073, SRMR =0.062(+). | 356 | Subscale Cronbach’s alpha =0.75 - 0.89. (+) |  |  |  |  |  |  |  |  |  |  |
| HIVMS (Audet 2015)^150^ | 125 | EFA showed that all four of the HIVMS items loaded significantly on a single component that explained 62.5% of the variance. (?) | 125 | Scale Cronbach’s alpha =0.80. (+) |  |  | 125 | Only a describe in Abstract: acceptable test-retest reliability (0.70). (+) |  |  | 125 | Results in line with 7 hypo (7+). |  |  |
| PLHIV-RS (Gottert 2019)^151^ | 1207 | Unidimensional model: RMSEA in 3 countries: 0.06 - 0.08; TLI in Cameroon: 0.93; TLI in Uganda:0.89; All other cut-of criteria were met. (+) | 1207 | Cameroon Cronbach’s alpha =0.81; Senegal Cronbach’s alpha =0.92; Uganda Cronbach’s alpha =0.89. (+) |  |  |  |  |  |  | 1207 | Results in line with 3 hypo (3+). |  |  |
| BIS (Martinez 2005)^152^ | 147 | Unidimensional scale. (?) | 147 | 3 contexts (now, before, and others) Cronbach’s alpha =0.90, 0.95, 0.90, respectively. (+) |  |  | 38 | Scale ICC =0.72. (+) |  |  |  |  |  |  |
| OCLS (Lee 2006)^153^ | 107 | 3-factor solution. (?) | 107 | Patient Cronbach’s alpha =0.76. (+) |  |  |  |  |  |  |  |  |  |  |
| ACTG-ABCD (Guaraldi 2006)^154^ |  |  | 330 | Part 3 Cronbach’s alpha =0.94. (+) |  |  |  |  |  |  | 330 | Results in line with 4 hypo (4+); Results not in line with 4 hypo (4-). |  |  |
| ACTG-ABCD-SF (Blashill 2014)^155^ | 106 | 4 factors accounting for 65 % of the variance. (?) | 106 | 18 items: Scale Cronbach’s alpha =0.92; Subscale Cronbach’s alpha =0.62 - 0.93. (-)  10 items: Scale Cronbach’s alpha =0.84; Subscale Cronbach’s alpha =0.62 - 0.88. (-) |  |  |  |  |  |  | 106 | Results in line with 5 hypo (5+); Result not in line with 1 hypo (1-). |  |  |
| FAI (Wu 2016)^156^ |  |  | 96 | Scale Cronbach’s alpha =0.98. (+) |  |  |  |  |  |  | 96 | Results in line with 5 hypo (5+). |  |  |
| MAS (Aversa 1998)^157^ |  |  | 62 | Scale Cronbach’s alpha =0.89. (+) |  |  |  |  |  |  | 62 | Results in line with 3 hypo (3+); Results not in line with 2 hypo (2-). |  |  |
| HIVTSQ (Woodcock 2001)^158^ | 150 | 2-factor solution (9 items) accounted for 57.8% of the variance. (?) | 150 | Scale Cronbach’s alpha =0.82; G Subscale Cronbach’s alpha =0.80; L Subscale Cronbach’s alpha =0.74. (+) |  |  |  |  |  |  | 150 | Result in line with 1 hypo (1+); Result not in line with 1 hypo (1-). |  |  |
| HIVTSQ-s (Woodcock 2006)^159^ | 152 | NFI =0.95. (+) | 152 | Treatment satisfaction scale alpha =0.891; General satisfaction/clinical Subscale alpha =0.831; Lifestyle/ease Subscale alpha =0.821. (+) |  |  |  |  |  |  | 152 | Result in line with 1 hypo (1+); Results not in line with 2 hypo (2-). |  |  |
| TES (Webb 2001)^160^ |  |  | 43 | Scale Cronbach’s alpha =0.85. (+) |  |  |  |  |  |  | 43 | Results in line with 3 hypo (3+); Results not in line with 2 hypo (2-). |  |  |
| SIS (Green 2002)^161^ | 547 | Daily functioning (r =0.61 to r =0.80), Ease of injection (r =0.68 to r =0.76), and Activities of daily living (r =0.83 to r =0.89). (?) | 547 | Daily functioning (Cronbach’s alpha =0.86), ease of injection (Cronbach’s alpha =0.71), and activities of daily living (Cronbach’s alpha =0.82). (+) |  |  |  |  |  |  | 547 | Results in line with 1 hypo (2+); Result not in line with 1 hypo (1-). |  |  |
| QUOTE-HIV (Hekkink 2003)^162^ |  |  | 44 | GP importance Cronbach’s alpha =0.81; GP performance Cronbach’s alpha =0.95; SP importance Cronbach’s alpha =0.85; SP performance Cronbach’s alpha =0.92; AC importance Cronbach’s alpha =0.91; AC performance Cronbach’s alpha =0.92. (+) |  |  |  |  |  |  |  |  |  |  |
| AHHCP (Bodenlos 2004)^163^ | 129 | 2-factor solution explained 53.3% of the variance. (?) | 129 | Scale Cronbach’s alpha =0.92; Professionalism Subscale Cronbach’s alpha =0.87; Emotional Support Subscale Cronbach’s alpha =0.89. (+) |  |  |  |  |  |  | 129 | Results in line with 2 hypo (2+). |  |  |
| AGAS (Holstad 2010)^164^ | GBL: 247; KHARMA: 207. | GBL: One factor emerged that explained 57.22% of the variance; KHARMA: One factor emerged that explained 64.87% of the variance. (?) | GBL: 247; KHARMA: 207. | GBL: Scale Cronbach’s alpha =0.80; KHARMA: Scale Cronbach’s alpha =0.85. (+) |  |  |  |  |  |  | GBL: 247; KHARMA: 207. | GBL: Results in line with 4 hypo (4+); Result not in line with 1 hypo (1-).; KHARMA: Results in line with 5 hypo (5+). |  |  |
| HCR (Bova 2006)^165^ | 99 | Three-factor solution explained 69% of the variance. (?) | 99 | Scale Cronbach’s alpha =0.92; Subscale Cronbach’s alpha =0.81 - 0.89. (+) |  |  | 27 | Scale ICC =0.59. (-) |  |  | 99 | Result in line with 1 hypo (1+); Results not in line with 2 hypo (2-). |  |  |
| HMRS (Balfour 2007)^166^ | 142 | One factor (unidimensional factor structure) accounted for 53.2% of the variance. (?) | 142 | Scale Cronbach’s alpha =0.90. (+) |  |  | 31 | Scale Pearson’s r =0.83. (+) |  |  | 142 | Results in line with 3 hypo (3+). | 142 | Result in line with 1 hypo (1+). |
| SECope (Johnson 2007)^167^ | 233 | TLI =0.95, RMSEA =0.10, SRMR =0.08. (+) | 233 | Scale Cronbach’s alpha =0.87; Subscale Cronbach’s alpha =0.68 - 0.86. (-) |  |  | 170 | Scale ICC =0.87; Subscale ICC =0.69 - 0.83. (-) |  |  | 233 | Results in line with 14 hypo (14+); Results not in line with 7 hypo (7-). |  |  |
| HTOS (Brennan 2009)^168^ | 303 | 3-factor solution explained 53.5% of the variance. (?) | 346 | Scale Cronbach’s alpha =0.76; Subscale Cronbach’s alpha =0.71 - 0.86. (+) |  |  |  |  |  |  |  |  |  |  |
| HIV-MT-SES (Erlen 2010)^169^ | 326 | CFI =0.96, RMSEA =0.046 (95% CIs: 0.0289 to 0.0597). (+) | 326 | Scale Cronbach’s alpha =0.93; SEB Cronbach’s alpha =0.94, OE Cronbach’s alpha =0.93. (+) |  |  | 99 | Scale r =0.73; SEB r =0.66; OE r =0.73. (?) |  |  | 326 | Results in line with 4 hypo (4+). |  |  |
| BEHKA-HIV (Osborn 2010)^170^ | 204 | 2 factors explained 58% total percent of variance. (?) | 204 | Knowledge Subscale Cronbach’s alpha =0.73, Action Subscale Cronbach’s alpha =0.79. (+) |  |  |  |  |  |  |  |  |  |  |
| HTRM (Fernández 2011)^171^ | 201 | 5 factors explained 47.1% total percent of variance. (?) | 201 | Scale Cronbach’s alpha =0.842; Subscale Cronbach’s alpha =0.623 - 0.826. (-) |  |  | 179 | 2 items with low correlation (<0.3); The sum scores r =0.857; The mean scores r =0.865. (-) |  |  |  |  |  |  |
| HTRFS (Claborn 2015)^172^ | 96 | 2-factor solution accounted for 35.8% of the variance. (?) | 96 | Treatment Cynicism Cronbach’s alpha =0.89; Self-Efficacy Cronbach’s alpha =0.72. (+) |  |  |  |  |  |  | 96 | Results in line with 7 hypo (7+); Results not in line with 2 hypo (2-). |  |  |
| HECCS (Logie 2017)^173^ | 173 | CFI =0.998, TLI =0.990, RMSEA =0.032. (+) | 173 | Scale Cronbach’s alpha =0.903; Subscale Cronbach’s alpha =0.70 - 0.90. (+) |  |  |  |  |  |  | 173 | Result in line with 1 hypo (1+). |  |  |
| SSI (Renwick 1999)^174^ | 120 | 3 factors for revised 17-items version. (?) | 120 | Instrumental Cronbach’s alpha =0.45, 0.52, 0.38; Informational Cronbach’s alpha =0.66, 0.37, 0.67; Emotional Cronbach’s alpha =0.82, 0.80, 0.89. (-) |  |  |  |  |  |  | 120 | Results in line with 2 hypo (2+). |  |  |
| USII-HIV (Ingram 1999)^175^ | 271 | 4-factor solution accounted for 94% of the common variance. (?) | 271 | Subscale Cronbach’s alpha =0.81 - 0.88. (+) |  |  |  |  |  |  | 271 | Results in line with 7 hypo (7+); Results not in line with 13 hypo (13-). |  |  |
| PSS-HIV (Cortes 2014)^176^ |  |  | Chile: 24  UK: 12 | UK: Scale Cronbach’s alpha =0.910; Subscale Cronbach’s alpha =0.635 - 0.948. (-)  Chile: Scale Cronbach’s alpha =0.937; Subscale Cronbach’s alpha =0.833 - 0.924. (+) |  |  |  |  |  |  | Chile: 24  UK: 12 | Results in line with 3 hypo (3+). |  |  |
| HIV-ASES (Johnson 2007)^177^ | Study 1: 264; Study 2: 2848. | Study 1: 2 factors CFA: CFI =0.95, RMSEA =0.06, and SRMR =0.05; Study 2: 2 factors CFA: CFI =0.92, RMSEA =0.07, SRMR =0.04. (+) | Study 1: 264; Study 2: 2848. | Study 1: Integration Raykov’s coefficient ρ =0.90, Perseverance Raykov’s coefficient ρ =0.75; Study 2: Integration Raykov’s coefficient ρ =0.91, Perseverance Raykov’s coefficient ρ =0.79. (+) |  |  | Study 1: 264; Study 2: 232. | 3 months Study 1: Integration r =0.71, Perseverance r =0.71;  15 months Study 2: Integration r =0.49, Perseverance r =0.43. |  |  | Study 1: 264; Study 2: 2848. | Study 1: Results in line with 32 hypo (32+); Results not in line with 6 hypo (6-); Study 2: Results in line with 26 hypo (26+); Results not in line with 2 hypo (2-). |  |  |
| PHIVSMS (Wallston 2011)^178^ |  |  | 121 | Scale Cronbach’s alpha =0.78. (+) |  |  |  |  |  |  | 125 | Results in line with 6 hypo (6+). |  |  |
| HIV-SMS-W (Webel 2012)^179^ | 260 | EFA: 3 factors, 48.6％ of the variance being explained; CFA: CFI =0.93, TLI =0.91, RMSEA =0.050. (+) | 260 | Subscale Cronbach’s alpha =0.72 - 0.86. (+) |  |  | 108 | Subscale Cronbach’s alpha =0.61 - 0.85. (?) |  |  |  |  |  |  |
| HIV-IM (Nelsen 2012)^180^ | 287 | 2-factor solution accounted for 86% of the variance. (?) | 287 | Intention Cronbach’s alpha =0.81; Knowledge Cronbach’s alpha =0.83. (+) |  |  |  |  |  |  | 287 | Results in line with 2 hypo (2+). |  |  |
| HIVESS (Gray 2016)^181^ | 133 | CFI =0.92; TLI =0.91; RMSEA =0.07. (-) | 133 | EB Cronbach’s alpha =0.81; ER Cronbach’s alpha =0.71; LCE Cronbach’s alpha =0.85. (+) |  |  |  |  |  |  | 107 | Results in line with 3 hypo (3+). |  |  |
| HSM-SEWS (Webel 2011)^182^ | 89 | 1-factor solution accounted for 93% of the variance. (?) | 89 | Scale Cronbach’s alpha =0.92. (+) |  |  | 89 | Scale Cronbach’s alpha =0.92, 0.93, 0.86, 0.95 respectively (every four weeks). (?) |  |  |  |  |  |  |

Abbreviations: PROM, Patient Reported Outcome Measures; AIDS, Acquired Immune Deficiency Syndrome; HIV, Human Immunodeficiency Virus; ART, antiretroviral therapy; PLWHA, people living with HIV/AIDS; CCV/MI, Cross-cultural validity/ measurement invariance; HTCV: Hypotheses testing for construct validity; ICC, intraclass correlation coefficient; QoL, quality of life; EFA, exploratory factor analysis; CFA, confirmatory factor analysis; CFI, comparative fit index; TLI, tucker-lewis Index; RMSEA, root mean square error of approximation; SRMR, standardized root mean square residual; GFI, goodness of fit index; AGFI, adjusted goodness of fit index; NFI, normed fit index; NNFI, non-normed fit index; KMO, Kaiser-Meyer-Olkin; SDC, smallest detectable change;

MOS-HIV, Medical Outcomes Study-HIV Health Survey; HOPES, HIV Overview of Problems Evaluation System; HIV-QoL, HIV-related Quality of Life Questions; AIDS-HAQ, AIDS Health Assessment Questionnaire; HIV-PARSE, HIV Patient Reported Status and Experience; HRQOL, Health-Related Quality of Life; FAHI, Functional Assessment of HIV Infection; GHSA, General Health Self-Assessment; HIV-QL31, HIV Quality of Life 31 items Scale; HAT-QoL, HIV/AIDS Targeted QOL Instrument; MQoL-HIV, Multidimensional QOL for Patients with HIV/AIDS; LWHIVS, Living with HIV Scale; WHOQOL-HIV, World Health Organization Quality of Life HIV; ISSQoL, Instituto Superiore di Sanità Quality of Life; HIV-SQUAD, Symptom Quality of Life Adherence; PROQOL-HIV, Patient Reported Outcome Quality of Life-HIV Questionnaire; PozQol, Poz Quality of Life; RSC, Riverside Symptom Checklist; HSI, HIV Symptom Index; HAT, HIV Assessment Tool; SSC-HIV, Sign and Symptom Checklist for HIV; HCSUS-SM, HIV Cost and Services Utilization Study Symptom Measure; HIV-SI/SDM, HIV Symptom Index or Symptoms Distress Module of the ACTG; HRFS, HIV-Related Fatigue Scale; HDQ, HIV Disability Questionnaire; ISS-HIV-SS, Istituto Superiore di Sanità-HIV symptoms scale; HSS-40, HIV Stigma Scale; HASI-P, HIV/AIDS Stigma Instrument-PLWA; IHSM, Internalized HIV Stigma measure; IA-RSS, Internalized AIDS-Related Stigma Scale; ISAT, Internalized Stigma in those with HIV/AIDS; HARSI, HIV and Abuse Related Shame Inventory; SEP-HASS, Self, experienced, and perceived HIV/AIDS stigma scales; HIV-SM, HIV stigma mechanisms; HA-SAL-GBT, HIV/AIDS stigma assessment for Latino gay men, bisexual men and transgender women living with HIV; VR-HARSSR, Van Rie HIV/AIDS-Related Stigma Scale -Revised for use in the USA; MAH, The Mental Adjustment to HIV scale; SS-HIV, HIV/AIDS Stress Scale; PSSHIV, Perceived Stress Scale among PLWHA; ISCS, Impact on Self-Concept Scale; IHIV, Impact of HIV; HIVMS, HIV Meaningfulness Scale; PLHIV-RS, People Living with HIV Resilience Scale; BIS, Body Image in Patients with HIV/AIDS; OCLS, Owen Clinic Lipodystrophy Scale; ACTG-ABCD, Adult AIDS Clinical Trial Group's Assessment of Body Change and Distress; ACTG-ABCD Short form, ACTG-ABCD-SF; FAI, Facial Appearance Inventory; MAS, Medication Attribution Scale; HIVTSQ, HIV Treatment Satisfaction Questionnaire; TES, Treatment-related Empowerment Scale; SIS, Subcutaneous Injection Survey; QUOTE-HIV, Quality of care through the patient’s eyes; AHHCP, Attitudes toward HIV Health Care Provider scale; AGAS, Antiretroviral General Adherence Scale; HCR, Health Care Relationship Trust Scale; HMRS, HIV Medication Readiness Scale; HTOS, HIV Treatment Optimism Scale; HIV-MT-SES, HIV Medication Taking Self-Efficacy Scale; BEHKA-HIV, Brief Estimate of Health Knowledge and Action-HIV version; HTRM, HIV Treatment Readiness Measure; HTRFS, HIV Treatment Regimen Fatigue Scale; HECCS, HIV Engagement in and Continuity of Care Scale; SSI, Social Support Inventory; USII-HIV, Unsupportive Social Interactions Inventory –HIV version; PSS-HIV, Perceived Social Support for HIV; HIV-ASES, HIV Treatment Adherence Self-Efficacy Scale; PHIVSMS, Perceived HIV Self-Management Scale; HIV-SMS-W, HIV Self-management Scale (Women); HIV-IM, HIV Intention Measure; HIVESS, HIV Exercise Stereotypes Scale; HSM-SEWS, HIV Symptom Management Self-Efficacy for Women Scale.

Due to there is no generally accepted "gold-standard" for assessing PRO for PLWHA, the criterion validity of all the study was not assessed.

Result rated as: “+” = positive rating, “?” = indeterminate rating, “±” = inconsistent rating, “-” = negative rating, “empty boxes” = not reported.

## Table S7. The Overall Results and the Level of Evidence

| **PROM (Author Year)** | **Content validity** | | | | **Structural validity** | | **Internal consistency^a^** | | **CCV/MI** | | **Reliability** | | **Measurement error** | | **HTCV^b^** | | **Responsiveness** | |
| --- | --- | --- | --- | --- | --- | --- | --- | --- | --- | --- | --- | --- | --- | --- | --- | --- | --- | --- |
|  | **REL** | **COMN** | **COMB** | **Rating** | **N** | **Rating** | **N** | **Rating** | **N** | **Rating** | **N** | **Rating** | **N** | **Rating** | ***N*** | **Rating** | ***N*** | **Rating** |
| MOS-HIV (Carretero 1996)^31^ |  |  |  |  |  |  | 100 | - |  |  |  |  |  |  | 100 | + |  |  |
| MOS-HIV (Murri 1997)^32^ |  |  |  |  |  |  | 213 | + |  |  |  |  |  |  | 213 | - |  |  |
| MOS-HIV (Revicki 1998)^33^ |  |  |  |  | 1020 | + | 2385 | + |  |  | 134 | - |  |  | 2385 | + |  |  |
| MOS-HIV (Badia 1999)^34^ |  |  |  |  |  |  | 275 | + |  |  | 48 | - |  |  | 275 | - |  |  |
| MOS-HIV (Holmes 1999)^35^ |  |  |  |  |  |  | 215 | + |  |  |  |  |  |  | 215 | + |  |  |
| MOS-HIV (Scott-Lennox 1999)^36^ |  |  |  |  | 363 | ? | 363 | - |  |  |  |  |  |  | 363 | - |  |  |
| MOS-HIV (Delate 2001)^37^ |  |  |  |  |  |  | 242 | + |  |  |  |  |  |  | 242 | - |  |  |
| MOS-HIV (Paton 2002)^38^ |  |  |  |  | 163 | ? | 163 | + |  |  |  |  |  |  | 163 | + |  |  |
| MOS-HIV (Schifano 2003)^39^ |  |  |  |  |  |  | 146 | + |  |  |  |  |  |  | 146 | - |  |  |
| MOS-HIV (Ichikawa 2004)^40^ |  |  |  |  | 200 | ? | 200 | + |  |  |  |  |  |  | 200 | + |  |  |
| MOS-HIV (Lau 2006)^41^ |  |  |  |  | 242 | ? | 242 | + |  |  |  |  |  |  | 242 | - |  |  |
| MOS-HIV (Taylor 2009)^42^ |  |  |  |  | 400 | ? | 400 | - |  |  |  |  |  |  | 400 | + |  |  |
| MOS-HIV (Henderson 2010)^43^ |  |  |  |  | 532 | + | 532 | + |  |  |  |  |  |  | 532 | + |  |  |
| MOS-HIV (Stasinopoulou 2010)^44^ |  |  |  |  |  |  | 154 | + |  |  |  |  |  |  | 154 | + |  |  |
| MOS-HIV (Chariyalertsak 2011)^45^ |  |  |  |  |  |  | 50 | - |  |  |  |  |  |  | 50 | - |  |  |
| MOS-HIV (Hsiung 2011)^46^ |  |  |  |  | 619 | + | 619 | - |  |  |  |  |  |  | 619 | - |  |  |
| MOS-HIV (Epino 2012)^47^ |  |  |  |  | 610 | ? | 610 | + |  |  |  |  |  |  | 610 | + |  |  |
| MOS-HIV (Huang 2012)^48^ |  |  |  |  |  |  | 120 | + |  |  |  |  |  |  | 120 | - |  |  |
| MOS-HIV (Marzieh 2017)^49^ |  |  |  |  | 100 | + | 100 | - |  |  |  |  |  |  |  |  |  |  |
| MOS-HIV (Liu 2018)^50^ |  |  |  |  | 635 | + | 635 | + |  |  | 60 | + |  |  | 635 | + |  |  |
| MOS-HIV (Shim 2018)^51^ |  |  |  |  | 201 | + | 201 | + |  |  |  |  |  |  | 201 | - |  |  |
| Overall Rating |  |  |  |  | 5085 | + | 7965 | ± |  |  | 242 | ± |  |  | 7865 | + |  |  |
| LoE |  |  |  |  |  | M |  | M |  |  |  | M |  |  |  | H |  |  |
| MOS-HIV-29 (Stangl 2012)^52^ |  |  |  |  | 947 | ? | 947 | ? |  |  |  |  |  |  | 947 | + | 947 | + |
| Overall Rating |  |  |  |  | 947 | ? | 947 | ? |  |  |  |  |  |  | 947 | + | 947 | + |
| LoE |  |  |  |  |  | M |  | H |  |  |  |  |  |  |  | L |  | L |
| MOS-HIV-17 (McDonnell 2000)^53^ |  |  |  |  |  |  | 287 | ? |  |  |  |  |  |  | 287 | - |  |  |
| Overall Rating |  |  |  |  |  |  | 287 | ? |  |  |  |  |  |  | 287 | - |  |  |
| LoE |  |  |  |  |  |  |  | H |  |  |  |  |  |  |  | L |  |  |
| HOPES (Schag 1992)^54^ |  |  |  |  |  |  | 318 | ? |  |  |  |  |  |  | 318 | + |  |  |
| HOPES (De Boer 1996)^55^ |  |  |  |  |  |  | 106 | ? |  |  |  |  |  |  | 106 | - | 65 | + |
| Overall Rating |  |  |  |  |  |  | 424 | ? |  |  |  |  |  |  | 424 | + | 65 | + |
| LoE |  |  |  |  |  |  |  | H |  |  |  |  |  |  |  | VL |  | VL |
| HIV-QoL (Cleary 1993)^56^ |  |  |  |  |  |  | 189 | ? |  |  |  |  |  |  |  |  |  |  |
| Overall Rating |  |  |  |  |  |  | 189 | ? |  |  |  |  |  |  |  |  |  |  |
| LoE |  |  |  |  |  |  |  | H |  |  |  |  |  |  |  |  |  |  |
| AIDS-HAQ (Lubeck 1997)^57^ |  |  |  |  |  |  | 1083 | ? |  |  |  |  |  |  | 1083 | + | 297 | + |
| Overall Rating |  |  |  |  |  |  | 1083 | ? |  |  |  |  |  |  | 1083 | + | 297 | + |
| LoE |  |  |  |  |  |  |  | H |  |  |  |  |  |  |  | H |  | L |
| HIV-PARSE (Bozzette 1994)^58^ |  |  |  |  |  |  | 1862 | ? |  |  |  |  |  |  |  |  |  |  |
| Overall Rating |  |  |  |  |  |  | 1862 | ? |  |  |  |  |  |  |  |  |  |  |
| LoE |  |  |  |  |  |  |  | H |  |  |  |  |  |  |  |  |  |  |
| HIV-PARSE-Brief (Bozzette 1995)^59^ |  |  |  |  |  |  | 1934 | ? |  |  |  |  |  |  |  |  |  |  |
| Overall Rating |  |  |  |  |  |  | 1934 | ? |  |  |  |  |  |  |  |  |  |  |
| LoE |  |  |  |  |  |  |  | H |  |  |  |  |  |  |  |  |  |  |
| HRQOL (Hays 1995)^60^ |  |  |  |  | 205 | - | 205 | ? |  |  |  |  |  |  | 205 | + |  |  |
| Overall Rating |  |  |  |  | 205 | - | 205 | ? |  |  |  |  |  |  | 205 | + |  |  |
| LoE |  |  |  |  |  | VL |  | H |  |  |  |  |  |  |  | L |  |  |
| FAHI (Cella 1996)^61^ |  |  |  |  |  |  | 244 | ? |  |  |  |  |  |  | 201 | + |  |  |
| FAHI (Peterman 1997)^62^ |  |  |  |  | 361 | - | 361 | ? |  |  |  |  |  |  | 361 | + |  |  |
| FAHI (Viala-Danten 2010)^63^ |  |  |  |  |  |  | 1654 | ? |  |  |  |  |  |  | 1654 | + | 1654 | + |
| FAHI (Byrne 2013)^64^ |  |  |  |  |  |  | 170 | ? |  |  |  |  |  |  | 170 | - |  |  |
| FAHI (Nyongesa 2017)^65^ | ? | + | + | ± | 103 | ? | 103 | ? |  |  |  |  |  |  | 103 | + |  |  |
| Overall Rating | ? | + | + | ± | 464 | - | 2532 | ? |  |  |  |  |  |  | 2489 | + | 1654 | + |
| LoE |  |  |  | M |  | M |  | H |  |  |  |  |  |  |  | H |  | H |
| GHSA (Lenderking 1997)^66^ |  |  |  |  | 1694 | ? | 1694 | ? |  |  |  |  |  |  | 1694 | + |  |  |
| Overall Rating |  |  |  |  | 1694 | ? | 1694 | ? |  |  |  |  |  |  | 1694 | + |  |  |
| LoE |  |  |  |  |  | M |  | H |  |  |  |  |  |  |  | L |  |  |
| HIV-QL31 (Leplege 1997)^67^ | ? | + | ? | ? | 102 | - | 102 | ? |  |  |  |  |  |  | 102 | - |  |  |
| Overall Rating | ? | + | ? | ? | 102 | - | 102 | ? |  |  |  |  |  |  | 102 | - |  |  |
| LoE |  |  |  | VL |  | M |  | H |  |  |  |  |  |  |  | L |  |  |
| HAT-QoL-42 (Holmes 1997)^68^ |  |  |  |  |  |  | 106 | ? |  |  |  |  |  |  | 106 | - |  |  |
| HAT-QoL-42 (Holmes 1998)^69^ |  |  |  |  |  |  | 201 | ? |  |  |  |  |  |  | 201 | - |  |  |
| Overall Rating |  |  |  |  |  |  | 307 | ? |  |  |  |  |  |  | 307 | - |  |  |
| LoE |  |  |  |  |  |  |  | H |  |  |  |  |  |  |  | M |  |  |
| HAT-QoL-30 (Holmes 1999)^35^ |  |  |  |  |  |  | 215 | ? |  |  |  |  |  |  | 215 | + |  |  |
| Overall Rating |  |  |  |  |  |  | 215 | ? |  |  |  |  |  |  | 215 | + |  |  |
| LoE |  |  |  |  |  |  |  | H |  |  |  |  |  |  |  | L |  |  |
| HAT-QoL-34 (Holmes 2008)^70^ |  |  |  |  |  |  | 153 | ? |  |  | 60 | - |  |  |  |  |  |  |
| HAT-QoL-34 (Taylor 2009)^42^ |  |  |  |  | 400 | ? | 400 | ? |  |  |  |  |  |  | 400 | + |  |  |
| HAT-Qol-34 (Mafirakureva 2016)^71^ |  |  |  |  |  |  | 257 | ? |  |  |  |  |  |  | 257 | + |  |  |
| Overall Rating |  |  |  |  | 400 | ? | 810 | ? |  |  | 60 | - |  |  | 657 | + |  |  |
| LoE |  |  |  |  |  | M |  | H |  |  |  | M |  |  |  | H |  |  |
| MQoL-HIV (Smith 1997)^72^ |  |  |  |  |  |  | 121 | ? |  |  | 121 | - |  |  | 121 | + | 95 | + |
| MQoL-HIV (Badia 1999)^34^ |  |  |  |  |  |  | 283 | ? |  |  | 50 | - |  |  | 283 | - |  |  |
| MQoL-HIV (Kemmler 2003)^73^ |  |  |  |  |  |  | 207 | ? |  |  | 109 | - |  |  | 207 | + |  |  |
| MQoL-HIV (Remple 2004)^74^ | ? | + | ? | ? |  |  | 85 | ? |  |  | 43 | - |  |  | 85 | + |  |  |
| MQoL-HIV (Watanabe 2004)^75^ |  |  |  |  | 375 | ? | 375 | ? |  |  |  |  |  |  | 375 | + |  |  |
| Overall Rating | ? | + | ? | ? | 375 | ? | 1071 | ? |  |  | 323 | - |  |  | 1071 | + | 95 | + |
| LoE |  |  |  | M |  | M |  | H |  |  |  | H |  |  |  | H |  | M |
| LWHIVS (Holzemer 1998)^76^ | + | + | ? | ± | 187 | ? | 187 | ? |  |  |  |  |  |  | 187 | - |  |  |
| Overall Rating | + | + | ? | ± | 187 | ? | 187 | ? |  |  |  |  |  |  | 187 | - |  |  |
| LoE |  |  |  | VL |  | M |  | H |  |  |  |  |  |  |  | L |  |  |
| WHOQOL-HIV (Starace 2002)^77^ |  |  |  |  |  |  | 151 | - |  |  |  |  |  |  | 151 | - |  |  |
| WHOQOL-HIV (WHOQOL HIV Group 2004)^78^ |  |  |  |  | 1334 | + | 1334 | - |  |  |  |  |  |  |  |  |  |  |
| WHOQOL-HIV (Zimpel 2007)^79^ |  |  |  |  |  |  | 308 | - |  |  |  |  |  |  | 308 | + |  |  |
| WHOQOL-HIV (Canavarro 2011)^80^ |  |  |  |  |  |  | 200 | + |  |  |  |  |  |  | 200 | + |  |  |
| WHOQOL-HIV (Mweemba 2011)^81^ |  |  |  |  | 160 | ? | 160 | + |  |  |  |  |  |  |  |  |  |  |
| WHOQOL-HIV (Olsen 2013)^82^ |  |  |  |  |  |  |  |  | 20 | ? |  |  |  |  |  |  |  |  |
| WHOQOL-HIV (Reychler 2013)^83^ |  |  |  |  |  |  | 50 | + |  |  | 50 | + |  |  | 50 | 10+ |  |  |
| Overall Rating |  |  |  |  | 1494 | + | 2203 | ± | 20 | ? | 50 | + |  |  | 709 | + |  |  |
| LoE |  |  |  |  |  | M |  | L |  | VL |  | M |  |  |  | H |  |  |
| WHOQOL-HIV-BREF (Saddki 2009)^84^ |  |  |  |  | 157 | ? | 157 | ? |  |  | 51 | - |  |  | 157 | + |  |  |
| WHOQOL-HIV-BREF (Hsiung 2011)^85^ |  |  |  |  | 680 | + | 680 | ? |  |  |  |  |  |  | 680 | + |  |  |
| WHOQOL-HIV-BREF (O'Connell 2012)^86^ |  |  |  |  | 1923 | + | 1923 | ? |  |  |  |  |  |  | 1923 | + |  |  |
| WHOQOL-HIV-BREF (Tran 2012)^87^ |  |  |  |  | 1016 | ? | 1016 | ? |  |  |  |  |  |  | 1016 | - |  |  |
| WHOQOL-HIV-BREF (Canavarro 2012)^88^ |  |  |  |  | 1196 | - | 1196 | ? |  |  |  |  |  |  | 1196 | + |  |  |
| WHOQOL-HIV-BREF (Pereira 2014)^89^ |  |  |  |  | 185 | + | 185 | ? |  |  |  |  |  |  | 185 | - |  |  |
| WHOQOL-HIV-BREF (Meemon 2016)^90^ |  |  |  |  | 329 | - | 329 | ? |  |  |  |  |  |  | 329 | - |  |  |
| WHOQOL-HIV-BREF (Salehi 2016)^91^ |  |  |  |  |  |  | 61 | ? |  |  |  |  |  |  |  |  |  |  |
| WHOQOL-HIV-BREF (Tesfaye 2016)^92^ |  |  |  |  | 348 | - | 348 | ? |  |  |  |  |  |  | 348 | + |  |  |
| WHOQOL-HIV-BREF (Zhu 2017)^93^ |  |  |  |  | 1100 | - | 1100 | ? |  |  | 57 | + |  |  | 1100 | + |  |  |
| WHOQOL-HIV-BREF  (Fuster-Ruizde Apodaca 2019)^94^ |  |  |  |  | 1462 | + | 1462 | ? |  |  |  |  |  |  | 1462 | + |  |  |
| WHOQOL-HIV-BREF (Barger 2020)^95^ |  |  |  |  | 586 | + | 586 | ? |  |  |  |  |  |  | 586 | + |  |  |
| Overall Rating |  |  |  |  | 8982 | ± | 9043 | ? |  |  | 108 | ± |  |  | 8982 | + |  |  |
| LoE |  |  |  |  |  | L |  | H |  |  |  | M |  |  |  | L |  |  |
| ISSQoL (Bucciardini 2006)^96^ | + | + | + | + |  |  | 322 | ? |  |  |  |  |  |  | 322 | + |  |  |
| Overall Rating | + | + | + | + |  |  | 322 | ? |  |  |  |  |  |  | 322 | + |  |  |
| LoE |  |  |  | L |  |  |  | H |  |  |  |  |  |  |  | L |  |  |
| HIV-SQUAD (Spire 2009)^97^ |  |  |  |  | 270 | ? | 282 | ? |  |  |  |  |  |  | 293 | + |  |  |
| Overall Rating |  |  |  |  | 270 | ? | 282 | ? |  |  |  |  |  |  | 293 | + |  |  |
| LoE |  |  |  |  |  | M |  | H |  |  |  |  |  |  |  | L |  |  |
| PROQOL-HIV-43 (Duracinsky 2012(1))^98^ | + | + | + | + |  |  |  |  |  |  |  |  |  |  |  |  |  |  |
| PROQOL-HIV-43 (Duracinsky 2012)^99^ |  |  |  |  | 791 | ? | 791 | ? |  |  | 34 | + |  |  | 791 | + |  |  |
| PROQOL-HIV-43 (Duracinsky 2014)^100^ |  |  |  |  |  |  |  |  |  |  | 59 | + |  |  |  |  |  |  |
| Overall Rating | + | + | + | + | 791 | ? | 791 | ? |  |  | 93 | + |  |  | 791 | + |  |  |
| LoE |  |  |  | VL |  | M |  | H |  |  |  | L |  |  |  | L |  |  |
| PROQOL-HIV-38 (Lalanne 2016)^101^ |  |  |  |  | 2526 | - | 1693 | ? |  |  |  |  |  |  | 1693 | + |  |  |
| Overall Rating |  |  |  |  | 2526 | - | 1693 | ? |  |  |  |  |  |  | 1693 | + |  |  |
| LoE |  |  |  |  |  | H |  | H |  |  |  |  |  |  |  | VL |  |  |
| PozQol (Brown 2018)^102^ | + | + | + | + | 465 | + | 465 | + |  |  | 51 | + |  |  | 465 | + |  |  |
| Overall Rating | + | + | + | + | 465 | + | 465 | + |  |  | 51 | + |  |  | 465 | + |  |  |
| LoE |  |  |  | L |  | H |  | H |  |  |  | M |  |  |  | M |  |  |
| RSC (Burgess1993)^103^ |  |  |  |  |  |  | 103 | ? |  |  |  |  |  |  | 103 | + |  |  |
| Overall Rating |  |  |  |  |  |  | 103 | ? |  |  |  |  |  |  | 103 | + |  |  |
| LoE |  |  |  |  |  |  |  | H |  |  |  |  |  |  |  | H |  |  |
| HSI (Whalen 1994)^104^ |  |  |  |  |  |  | 148 | ? |  |  | 21 | + |  |  | 148 | - | 60 | + |
| Overall Rating |  |  |  |  |  |  | 148 | ? |  |  | 21 | + |  |  | 148 | - | 60 | + |
| LoE |  |  |  |  |  |  |  | H |  |  |  | L |  |  |  | L |  | VL |
| HAT (Nokes 1994)^105^ |  |  |  |  | 156 | ? | 156 | ? |  |  | 156 | + |  |  |  |  |  |  |
| Overall Rating |  |  |  |  | 156 | ? | 156 | ? |  |  | 156 | + |  |  |  |  |  |  |
| LoE |  |  |  |  |  | VL |  | VL |  |  |  | L |  |  |  |  |  |  |
| SSC-HIV (Holzemer 1999)^107^ |  |  |  |  | 686 | ? | 686 | + |  |  |  |  |  |  |  |  |  |  |
| SSC-HIV (Sousa 2006)^106^ |  |  |  |  | 917 | + |  |  |  |  |  |  |  |  |  |  |  |  |
| Overall Rating |  |  |  |  | 1603 | + | 686 | + |  |  |  |  |  |  |  |  |  |  |
| LoE |  |  |  |  |  | M |  | H |  |  |  |  |  |  |  |  |  |  |
| SSC-HIV-rev (Holzemer 2001)^108^ |  |  |  |  | 372 | ? | 372 | ? |  |  |  |  |  |  | 372 | - |  |  |
| Overall Rating |  |  |  |  | 372 | ? | 372 | ? |  |  |  |  |  |  | 372 | - |  |  |
| LoE |  |  |  |  |  | M |  | H |  |  |  |  |  |  |  | L |  |  |
| HCSUS-SM (Mathews 2000)^109^ |  |  |  |  |  |  | 3072 | ? |  |  |  |  |  |  |  |  |  |  |
| Overall Rating |  |  |  |  |  |  | 3072 | ? |  |  |  |  |  |  |  |  |  |  |
| LoE |  |  |  |  |  |  |  | H |  |  |  |  |  |  |  |  |  |  |
| HIV-SI/SDM (Justice 2001)^110^ | + | + | + | + |  |  |  |  |  |  |  |  |  |  | 113 | + |  |  |
| HIV-SI/SDM (Regnault 2009)^111^ |  |  |  |  |  |  |  |  | 759 | - |  |  |  |  |  |  |  |  |
| HIV-SI/SDM (Marc 2012)^112^ |  |  |  |  | 5521 | + | 5521 | + |  |  |  |  |  |  | 5521 | + |  |  |
| Overall Rating | + | + | + | + | 5521 | + | 5521 | + | 759 | - |  |  |  |  | 5634 | + |  |  |
| LoE |  |  |  | L |  | H |  | H |  | L |  |  |  |  |  | L |  |  |
| HRFS (Barroso 2002)^113^ | ? | + | ? | ? |  |  | 54 | ? |  |  | 14 | - |  |  |  |  |  |  |
| HRFS (Pence 2008)^114^ |  |  |  |  |  |  | 128 | ? |  |  |  |  |  |  | 128 | + |  |  |
| HRFS (Li 2016)^115^ |  |  |  |  |  |  | 142 | ? |  |  | 142 | - |  |  | 142 | + |  |  |
| Overall Rating | ? | + | ? | ? |  |  | 324 | ? |  |  | 156 | - |  |  | 270 | + |  |  |
| LoE |  |  |  | VL |  |  |  | H |  |  |  | H |  |  |  | M |  |  |
| HDQ (O'Brien 2014)^116^ |  |  |  |  | 361 | + |  |  |  |  |  |  |  |  |  |  |  |  |
| HDQ (O'Brien 2015)^117^ |  |  |  |  |  |  | 235 | + |  |  | 99 | + |  |  | 235 | - |  |  |
| HDQ (Brown 2019)^118^ |  |  |  |  |  |  | 243 | + |  |  |  |  | 243 | ? | 243 | + |  |  |
| HDQ (O’Brien 2019)^119^ |  |  |  |  |  |  | 128 | + |  |  | 61 | + |  |  | 124 | + |  |  |
| Overall Rating |  |  |  |  | 361 | + | 478 | + |  |  | 160 | + | 243 | ? | 478 | ± |  |  |
| LoE |  |  |  |  |  | M |  | H |  |  |  | M |  | L |  | M |  |  |
| ISS-HIV-SS (Bucciardini 2016)^120^ |  |  |  |  | 161 | ? | 161 | ? |  |  |  |  |  |  | 161 | + |  |  |
| Overall Rating |  |  |  |  | 161 | ? | 161 | ? |  |  |  |  |  |  | 161 | + |  |  |
| LoE |  |  |  |  |  | M |  | H |  |  |  |  |  |  |  | H |  |  |
| HSS-40 (Berger 2001)^121^ |  |  |  |  | 318 | ? | 318 | ? |  |  | 139 | + |  |  | 318 | + |  |  |
| HSS-40 (Rao 2008)^122^ |  |  |  |  |  |  |  |  | 541 | - |  |  |  |  |  |  |  |  |
| Overall Rating |  |  |  |  | 318 | ? | 318 | ? | 541 | - | 139 | + |  |  | 318 | + |  |  |
| LoE |  |  |  |  |  | M |  | H |  | L |  | M |  |  |  | H |  |  |
| HSS-32 (Bunn 2007)^123^ |  |  |  |  | 157 | - | 157 | ? |  |  |  |  |  |  | 157 | + |  |  |
| HSS-32 (Reinius 2018)^124^ |  |  |  |  | 1036 | + |  |  | 1036 | - |  |  |  |  |  |  |  |  |
| Overall Rating |  |  |  |  | 1193 | ± | 157 | ? | 1036 | - |  |  |  |  | 157 | + |  |  |
| LoE |  |  |  |  |  | M |  | H |  | VL |  |  |  |  |  | L |  |  |
| HSS-12 (Rongkavilit 2010)^125^ |  |  |  |  | 70 | + | 70 | + |  |  |  |  |  |  | 70 | - |  |  |
| HSS-12 (Reinius 2017)^126^ |  |  |  |  | 880 | + | 880 | + |  |  |  |  |  |  |  |  |  |  |
| HSS-12 (Luz 2020)^127^ |  |  |  |  | 2102 | + | 2102 | + | 2102 | - |  |  |  |  | 2102 | - |  |  |
| Overall Rating |  |  |  |  | 3052 | + | 3052 | + | 2102 | - |  |  |  |  | 2172 | - |  |  |
| LoE |  |  |  |  |  | H |  | H |  | L |  |  |  |  |  | M |  |  |
| HSS-39 (Lindberg 2014)^128^ |  |  |  |  | 194 | ? | 194 | ? |  |  |  |  |  |  | 194 | + |  |  |
| Overall Rating |  |  |  |  | 194 | ? | 194 | ? |  |  |  |  |  |  | 194 | + |  |  |
| LoE |  |  |  |  |  | VL |  | H |  |  |  |  |  |  |  | VL |  |  |
| HSS-30 (Fuster-RuizdeApodaca 2015)^129^ | ? | + | ? | ? | 557 | + | 557 | + |  |  |  |  |  |  | 557 | + |  |  |
| Overall Rating | ? | + | ? | ? | 557 | + | 557 | + |  |  |  |  |  |  | 557 | + |  |  |
| LoE |  |  |  | M |  | H |  | H |  |  |  |  |  |  |  | H |  |  |
| HSS-10 (Kagiura 2020)^130^ |  |  |  |  | 451 | + | 451 | - |  |  |  |  |  |  | 451 | + |  |  |
| Overall Rating |  |  |  |  | 451 | + | 451 | - |  |  |  |  |  |  | 451 | + |  |  |
| LoE |  |  |  |  |  | H |  | H |  |  |  |  |  |  |  | L |  |  |
| HASI-P (Holzemer 2007)^131^ |  |  |  |  | 1477 | ? | 1477 | ? |  |  |  |  |  |  | 1477 | + |  |  |
| HASI-P (Maluccio 2017)^132^ |  |  |  |  | 639 | ? | 639 | ? |  |  |  |  |  |  | 639 | + |  |  |
| Overall Rating |  |  |  |  | 2116 | ? | 2116 | ? |  |  |  |  |  |  | 2116 | + |  |  |
| LoE |  |  |  |  |  | H |  | H |  |  |  |  |  |  |  | H |  |  |
| IHSM (Sayles 2008)^133^ |  |  |  |  | 202 | ? | 202 | ? |  |  |  |  |  |  | 202 | - |  |  |
| Overall Rating |  |  |  |  | 202 | ? | 202 | ? |  |  |  |  |  |  | 202 | - |  |  |
| LoE |  |  |  |  |  | M |  | H |  |  |  |  |  |  |  | L |  |  |
| IA-RSS (Kalichman 2009)^134^ |  |  |  |  |  |  | 2374 | + |  |  | 181 | - |  |  | 2374 | + |  |  |
| IA-RSS (Tsai 2013)^135^ |  |  |  |  | 456 | ? | 456 | + |  |  | 456 | - |  |  | 456 | + |  |  |
| IA-RSS (Geibel 2020)^136^ |  |  |  |  | 3016 | + | 3016 | + |  |  |  |  |  |  | 3016 | - |  |  |
| Overall Rating |  |  |  |  | 3472 | + | 5846 | + |  |  | 637 | - |  |  | 5846 | ± |  |  |
| LoE |  |  |  |  |  | H |  | H |  |  |  | M |  |  |  | M |  |  |
| ISAT (Phillips 2011)^137^ |  |  |  |  | 255 | ? | 255 | ? |  |  |  |  |  |  | 255 | + |  |  |
| Overall Rating |  |  |  |  | 255 | ? | 255 | ? |  |  |  |  |  |  | 255 | + |  |  |
| LoE |  |  |  |  |  | M |  | H |  |  |  |  |  |  |  | H |  |  |
| HARSI (Neufeld 2012)^138^ |  |  |  |  | 271 | ? | 271 | ? |  |  | 66 | - |  |  |  |  |  |  |
| Overall Rating |  |  |  |  | 271 | ? | 271 | ? |  |  | 66 | - |  |  |  |  |  |  |
| LoE |  |  |  |  |  | M |  | H |  |  |  | VL |  |  |  |  |  |  |
| SEP-HASS (Zelaya 2012)^139^ |  |  |  |  |  |  | 200 | ? |  |  |  |  |  |  | 200 | + |  |  |
| Overall Rating |  |  |  |  |  |  | 200 | ? |  |  |  |  |  |  | 200 | + |  |  |
| LoE |  |  |  |  |  |  |  | H |  |  |  |  |  |  |  | VL |  |  |
| HIV-SM (Earnshaw 2013)^140^ |  |  |  |  |  |  | 95 | ? |  |  |  |  |  |  | 95 | - |  |  |
| Overall Rating |  |  |  |  |  |  | 95 | ? |  |  |  |  |  |  | 95 | - |  |  |
| LoE |  |  |  |  |  |  |  | M |  |  |  |  |  |  |  | VL |  |  |
| HA-SAL-GBT (Molina 2013)^141^ |  |  |  |  | 170 | ? | 170 | ? |  |  |  |  |  |  | 170 | - |  |  |
| Overall Rating |  |  |  |  | 170 | ? | 170 | ? |  |  |  |  |  |  | 170 | - |  |  |
| LoE |  |  |  |  |  | VL |  | H |  |  |  |  |  |  |  | L |  |  |
| VR-HARSSR (Kipp 2015)^142^ |  |  |  |  | 85 | ? | 85 | ? |  |  |  |  |  |  | 85 | + |  |  |
| Overall Rating |  |  |  |  | 85 | ? | 85 | ? |  |  |  |  |  |  | 85 | + |  |  |
| LoE |  |  |  |  |  | L |  | M |  |  |  |  |  |  |  | M |  |  |
| MAH (Ross 1994)^143^ |  |  |  |  | 107 | ? | 107 | ? |  |  |  |  |  |  |  |  |  |  |
| Overall Rating |  |  |  |  | 107 | ? | 107 | ? |  |  |  |  |  |  |  |  |  |  |
| LoE |  |  |  |  |  | VL |  | H |  |  |  |  |  |  |  |  |  |  |
| SS-HIV (Niu 2016)^144^ |  |  |  |  | 667 | - | 667 | ? |  |  | 667 | + |  |  | 667 | + |  |  |
| Overall Rating |  |  |  |  | 667 | - | 667 | ? |  |  | 667 | + |  |  | 667 | + |  |  |
| LoE |  |  |  |  |  | H |  | H |  |  |  | M |  |  |  | L |  |  |
| PSSHIV (Su 2008)^145^ | + | + | ? | ± | 215 | ? | 215 | ? |  |  | 31 | + |  |  | 215 | + |  |  |
| Overall Rating | + | + | ? | ± | 215 | ? | 215 | ? |  |  | 31 | + |  |  | 215 | + |  |  |
| LoE |  |  |  | VL |  | M |  | H |  |  |  | L |  |  |  | L |  |  |
| Screenphiv (Remor 2012)^146^ | ? | + | ? | ? | 84 | + | 84 | - |  |  |  |  |  |  |  |  |  |  |
| Screenphiv (Remor 2016)^147^ | ? | + | ? | ? | 744 | + | 744 | - |  |  |  |  |  |  | 744 | + |  |  |
| Overall Rating | ? | + | ? | ? | 828 | + | 828 | - |  |  |  |  |  |  | 744 | + |  |  |
| LoE |  |  |  | L |  | H |  | H |  |  |  |  |  |  |  | H |  |  |
| ISCS (Golub 2013)^148^ |  |  |  |  | 129 | ? | 129 | ? |  |  |  |  |  |  | 129 | + |  |  |
| Overall Rating |  |  |  |  | 129 | ? | 129 | ? |  |  |  |  |  |  | 129 | + |  |  |
| LoE |  |  |  |  |  | M |  | H |  |  |  |  |  |  |  | L |  |  |
| IHIV (Buscher 2015)^149^ |  |  |  |  | 356 | + | 356 | + |  |  |  |  |  |  |  |  |  |  |
| Overall Rating |  |  |  |  | 356 | + | 356 | + |  |  |  |  |  |  |  |  |  |  |
| LoE |  |  |  |  |  | H |  | H |  |  |  |  |  |  |  |  |  |  |
| HIVMS (Audet 2015)^150^ |  |  |  |  | 125 | ? | 125 | ? |  |  | 125 | + |  |  | 125 | + |  |  |
| Overall Rating |  |  |  |  | 125 | ? | 125 | ? |  |  | 125 | + |  |  | 125 | + |  |  |
| LoE |  |  |  |  |  | M |  | H |  |  |  | L |  |  |  | VL |  |  |
| PLHIV-RS (Gottert 2019)^151^ | + | + | + | + | 1207 | + | 1207 | + |  |  |  |  |  |  | 1207 | + |  |  |
| Overall Rating | + | + | + | + | 1207 | + | 1207 | + |  |  |  |  |  |  | 1207 | + |  |  |
| LoE |  |  |  | L |  | H |  | H |  |  |  |  |  |  |  | L |  |  |
| BIS (Martinez 2005)^152^ |  |  |  |  | 147 | ? | 147 | ? |  |  | 38 | + |  |  |  |  |  |  |
| Overall Rating |  |  |  |  | 147 | ? | 147 | ? |  |  | 38 | + |  |  |  |  |  |  |
| LoE |  |  |  |  |  | M |  | L |  |  |  | VL |  |  |  |  |  |  |
| OCLS (Lee 2006)^153^ |  |  |  |  | 107 | ? | 107 | ? |  |  |  |  |  |  |  |  |  |  |
| Overall Rating |  |  |  |  | 107 | ? | 107 | ? |  |  |  |  |  |  |  |  |  |  |
| LoE |  |  |  |  |  | M |  | VL |  |  |  |  |  |  |  |  |  |  |
| ACTG-ABCD (Guaraldi 2006)^154^ |  |  |  |  |  |  | 330 | ? |  |  |  |  |  |  | 330 | - |  |  |
| Overall Rating |  |  |  |  |  |  | 330 | ? |  |  |  |  |  |  | 330 | - |  |  |
| LoE |  |  |  |  |  |  |  | H |  |  |  |  |  |  |  | L |  |  |
| ACTG-ABCD-SF (Blashill 2014)^155^ |  |  |  |  | 106 | ? | 106 | ? |  |  |  |  |  |  | 106 | + |  |  |
| Overall Rating |  |  |  |  | 106 | ? | 106 | ? |  |  |  |  |  |  | 106 | + |  |  |
| LoE |  |  |  |  |  | M |  | H |  |  |  |  |  |  |  | L |  |  |
| FAI (Wu 2016)^156^ | ? | + | ? | ? |  |  | 96 | ? |  |  |  |  |  |  | 96 | + |  |  |
| Overall Rating | ? | + | ? | ? |  |  | 96 | ? |  |  |  |  |  |  | 96 | + |  |  |
| LoE |  |  |  | L |  |  |  | VL |  |  |  |  |  |  |  | VL |  |  |
| MAS (Aversa 1998)^157^ |  |  |  |  |  |  | 62 | ? |  |  |  |  |  |  | 62 | - |  |  |
| Overall Rating |  |  |  |  |  |  | 62 | ? |  |  |  |  |  |  | 62 | - |  |  |
| LoE |  |  |  |  |  |  |  | M |  |  |  |  |  |  |  | M |  |  |
| HIVTSQ (Woodcock 2001)^158^ |  |  |  |  | 150 | ? | 150 | ? |  |  |  |  |  |  | 150 | - |  |  |
| Overall Rating |  |  |  |  | 150 | ? | 150 | ? |  |  |  |  |  |  | 150 | - |  |  |
| LoE |  |  |  |  |  | M |  | H |  |  |  |  |  |  |  | L |  |  |
| HIVTSQ-s (Woodcock 2006)^159^ |  |  |  |  | 152 | + | 152 | + |  |  |  |  |  |  | 152 | - |  |  |
| Overall Rating |  |  |  |  | 152 | + | 152 | + |  |  |  |  |  |  | 152 | - |  |  |
| LoE |  |  |  |  |  | H |  | H |  |  |  |  |  |  |  | L |  |  |
| TES (Webb 2001)^160^ |  |  |  |  |  |  | 43 | ? |  |  |  |  |  |  | 43 | - |  |  |
| Overall Rating |  |  |  |  |  |  | 43 | ? |  |  |  |  |  |  | 43 | - |  |  |
| LoE |  |  |  |  |  |  |  | L |  |  |  |  |  |  |  | VL |  |  |
| SIS (Green 2002)^161^ |  |  |  |  | 547 | ? | 547 | ? |  |  |  |  |  |  | 547 | - |  |  |
| Overall Rating |  |  |  |  | 547 | ? | 547 | ? |  |  |  |  |  |  | 547 | - |  |  |
| LoE |  |  |  |  |  | M |  | H |  |  |  |  |  |  |  | L |  |  |
| QUOTE-HIV (Hekkink 2003)^162^ |  |  |  |  |  |  | 44 | ? |  |  |  |  |  |  |  |  |  |  |
| Overall Rating |  |  |  |  |  |  | 44 | ? |  |  |  |  |  |  |  |  |  |  |
| LoE |  |  |  |  |  |  |  | L |  |  |  |  |  |  |  |  |  |  |
| AHHCP (Bodenlos 2004)^163^ |  |  |  |  | 129 | ? | 129 | ? |  |  |  |  |  |  | 129 | + |  |  |
| Overall Rating |  |  |  |  | 129 | ? | 129 | ? |  |  |  |  |  |  | 129 | + |  |  |
| LoE |  |  |  |  |  | M |  | H |  |  |  |  |  |  |  | H |  |  |
| AGAS (Holstad 2010)^164^ |  |  |  |  | 454 | ? | 454 | ? |  |  |  |  |  |  | 454 | + |  |  |
| Overall Rating |  |  |  |  | 454 | ? | 454 | ? |  |  |  |  |  |  | 454 | + |  |  |
| LoE |  |  |  |  |  | M |  | H |  |  |  |  |  |  |  | L |  |  |
| HCR (Bova 2006)^165^ | + | + | ? | ± | 99 | ? | 99 | ? |  |  | 27 | - |  |  | 99 | - |  |  |
| Overall Rating | + | + | ? | ± | 99 | ? | 99 | ? |  |  | 27 | - |  |  | 99 | - |  |  |
| LoE |  |  |  | VL |  | L |  | M |  |  |  | VL |  |  |  | VL |  |  |
| HMRS (Balfour 2007)^166^ |  |  |  |  | 142 | ? | 142 | ? |  |  | 31 | + |  |  | 142 | + | 142 | + |
| Overall Rating |  |  |  |  | 142 | ? | 142 | ? |  |  | 31 | + |  |  | 142 | + | 142 | + |
| LoE |  |  |  |  |  | M |  | H |  |  |  | VL |  |  |  | L |  | H |
| SECope (Johnson 2007)^167^ | + | + | ? | ± | 233 | + | 233 | - |  |  | 170 | - |  |  | 233 | - |  |  |
| Overall Rating | + | + | ? | ± | 233 | + | 233 | - |  |  | 170 | - |  |  | 233 | - |  |  |
| LoE |  |  |  | VL |  | H |  | H |  |  |  | M |  |  |  | L |  |  |
| HTOS (Brennan 2009)^168^ |  |  |  |  | 303 | ? | 346 | ? |  |  |  |  |  |  |  |  |  |  |
| Overall Rating |  |  |  |  | 303 | ? | 346 | ? |  |  |  |  |  |  |  |  |  |  |
| LoE |  |  |  |  |  | M |  | H |  |  |  |  |  |  |  |  |  |  |
| HIV-MT-SES (Erlen 2010)^169^ |  |  |  |  | 326 | + | 326 | + |  |  | 99 | - |  |  | 326 | + |  |  |
| Overall Rating |  |  |  |  | 326 | + | 326 | + |  |  | 99 | - |  |  | 326 | + |  |  |
| LoE |  |  |  |  |  | H |  | H |  |  |  | L |  |  |  | L |  |  |
| BEHKA-HIV (Osborn 2010)^170^ |  |  |  |  | 204 | ? | 204 | ? |  |  |  |  |  |  |  |  |  |  |
| Overall Rating |  |  |  |  | 204 | ? | 204 | ? |  |  |  |  |  |  |  |  |  |  |
| LoE |  |  |  |  |  | M |  | H |  |  |  |  |  |  |  |  |  |  |
| HTRM (Fernández 2011)^171^ |  |  |  |  | 201 | ? | 201 | ? |  |  | 179 | - |  |  |  |  |  |  |
| Overall Rating |  |  |  |  | 201 | ? | 201 | ? |  |  | 179 | - |  |  |  |  |  |  |
| LoE |  |  |  |  |  | M |  | H |  |  |  | M |  |  |  |  |  |  |
| HTRFS (Claborn 2015)^172^ |  |  |  |  | 96 | ? | 96 | ? |  |  |  |  |  |  | 96 | + |  |  |
| Overall Rating |  |  |  |  | 96 | ? | 96 | ? |  |  |  |  |  |  | 96 | + |  |  |
| LoE |  |  |  |  |  | L |  | M |  |  |  |  |  |  |  | VL |  |  |
| HECCS (Logie 2017)^173^ |  |  |  |  | 173 | + | 173 | + |  |  |  |  |  |  | 173 | + |  |  |
| Overall Rating |  |  |  |  | 173 | + | 173 | + |  |  |  |  |  |  | 173 | + |  |  |
| LoE |  |  |  |  |  | M |  | H |  |  |  |  |  |  |  | H |  |  |
| SSI (Renwick 1999)^174^ | + | + | ? | ± | 120 | ? | 120 | ? |  |  |  |  |  |  | 120 | + |  |  |
| Overall Rating | + | + | ? | ± | 120 | ? | 120 | ? |  |  |  |  |  |  | 120 | + |  |  |
| LoE |  |  |  | VL |  | M |  | H |  |  |  |  |  |  |  | VL |  |  |
| USII-HIV (Ingram 1999)^175^ |  |  |  |  | 271 | ? | 271 | ? |  |  |  |  |  |  | 271 | - |  |  |
| Overall Rating |  |  |  |  | 271 | ? | 271 | ? |  |  |  |  |  |  | 271 | - |  |  |
| LoE |  |  |  |  |  | M |  | H |  |  |  |  |  |  |  | L |  |  |
| PSS-HIV (Cortes 2014)^176^ |  |  |  |  |  |  | 36 | ? |  |  |  |  |  |  | 36 | + |  |  |
| Overall Rating |  |  |  |  |  |  | 36 | ? |  |  |  |  |  |  | 36 | + |  |  |
| LoE |  |  |  |  |  |  |  | L |  |  |  |  |  |  |  | VL |  |  |
| HIV-ASES (Johnson 2007)^177^ |  |  |  |  | 3112 | + | 3112 | + |  |  | 496 | ? |  |  | 3112 | + |  |  |
| Overall Rating |  |  |  |  | 3112 | + | 3112 | + |  |  | 496 | ? |  |  | 3112 | + |  |  |
| LoE |  |  |  |  |  | H |  | H |  |  |  | L |  |  |  | L |  |  |
| PHIVSMS (Wallston 2011)^178^ |  |  |  |  |  |  | 121 | ? |  |  |  |  |  |  | 125 | + |  |  |
| Overall Rating |  |  |  |  |  |  | 121 | ? |  |  |  |  |  |  | 125 | + |  |  |
| LoE |  |  |  |  |  |  |  | H |  |  |  |  |  |  |  | L |  |  |
| HIV-SMS-W (Webel 2012)^179^ | + | + | + | + | 260 | + | 260 | + |  |  | 108 | ? |  |  |  |  |  |  |
| Overall Rating | + | + | + | + | 260 | + | 260 | + |  |  | 108 | ? |  |  |  |  |  |  |
| LoE |  |  |  | VL |  | H |  | H |  |  |  | VL |  |  |  |  |  |  |
| HIV-IM (Nelsen 2012)^180^ | ? | + | + | ± | 287 | ? | 287 | ? |  |  |  |  |  |  | 287 | + |  |  |
| Overall Rating | ? | + | + | ± | 287 | ? | 287 | ? |  |  |  |  |  |  | 287 | + |  |  |
| LoE |  |  |  | L |  | L |  | H |  |  |  |  |  |  |  | L |  |  |
| HIVESS (Gray 2016)^181^ |  |  |  |  | 133 | - | 133 | ? |  |  |  |  |  |  | 107 | + |  |  |
| Overall Rating |  |  |  |  | 133 | - | 133 | ? |  |  |  |  |  |  | 107 | + |  |  |
| LoE |  |  |  |  |  | H |  | H |  |  |  |  |  |  |  | VL |  |  |
| HSM-SEWS (Webel 2011)^182^ |  |  |  |  | 89 | ? | 89 | ? |  |  | 89 | ? |  |  |  |  |  |  |
| Overall Rating |  |  |  |  | 89 | ? | 89 | ? |  |  | 89 | ? |  |  |  |  |  |  |
| LoE |  |  |  |  |  | L |  | M |  |  |  | VL |  |  |  |  |  |  |

Abbreviations: PROM, Patient Reported Outcome Measures; AIDS, Acquired Immune Deficiency Syndrome; HIV, Human Immunodeficiency Virus; ART, antiretroviral therapy; PLWHA, people living with HIV/AIDS; REL: Relevance; COMN: Comprehensiveness; COMB: Comprehensibility; CCV/MI, Cross-cultural validity/ measurement invariance; HTCV: Hypotheses testing for construct validity; LoE, Level of Evidence (using the Grading of Recommendations, Assessment, Development and Evaluations assessment tool); MOS-HIV, Medical Outcomes Study-HIV Health Survey; HOPES, HIV Overview of Problems Evaluation System; HIV-QoL, HIV-related Quality of Life Questions; AIDS-HAQ, AIDS Health Assessment Questionnaire; HIV-PARSE, HIV Patient Reported Status and Experience; HRQOL, Health-Related Quality of Life; FAHI, Functional Assessment of HIV Infection; GHSA, General Health Self-Assessment; HIV-QL31, HIV Quality of Life 31 items Scale; HAT-QoL, HIV/AIDS Targeted QOL Instrument; MQoL-HIV, Multidimensional QOL for Patients with HIV/AIDS; LWHIVS, Living with HIV Scale; WHOQOL-HIV, World Health Organization Quality of Life HIV; ISSQoL, Instituto Superiore di Sanità Quality of Life; HIV-SQUAD, Symptom Quality of Life Adherence; PROQOL-HIV, Patient Reported Outcome Quality of Life-HIV Questionnaire; PozQol, Poz Quality of Life; RSC, Riverside Symptom Checklist; HSI, HIV Symptom Index; HAT, HIV Assessment Tool; SSC-HIV, Sign and Symptom Checklist for HIV; HCSUS-SM, HIV Cost and Services Utilization Study Symptom Measure; HIV-SI/SDM, HIV Symptom Index or Symptoms Distress Module of the ACTG; HRFS, HIV-Related Fatigue Scale; HDQ, HIV Disability Questionnaire; ISS-HIV-SS, Istituto Superiore di Sanità-HIV symptoms scale; HSS-40, HIV Stigma Scale; HASI-P, HIV/AIDS Stigma Instrument-PLWA; IHSM, Internalized HIV Stigma measure; IA-RSS, Internalized AIDS-Related Stigma Scale; ISAT, Internalized Stigma in those with HIV/AIDS; HARSI, HIV and Abuse Related Shame Inventory; SEP-HASS, Self, experienced, and perceived HIV/AIDS stigma scales; HIV-SM, HIV stigma mechanisms; HA-SAL-GBT, HIV/AIDS stigma assessment for Latino gay men, bisexual men and transgender women living with HIV; VR-HARSSR, Van Rie HIV/AIDS-Related Stigma Scale -Revised for use in the USA; MAH, The Mental Adjustment to HIV scale; SS-HIV, HIV/AIDS Stress Scale; PSSHIV, Perceived Stress Scale among PLWHA; ISCS, Impact on Self-Concept Scale; IHIV, Impact of HIV; HIVMS, HIV Meaningfulness Scale; PLHIV-RS, People Living with HIV Resilience Scale; BIS, Body Image in Patients with HIV/AIDS; OCLS, Owen Clinic Lipodystrophy Scale; ACTG-ABCD, Adult AIDS Clinical Trial Group's Assessment of Body Change and Distress; ACTG-ABCD Short form, ACTG-ABCD-SF; FAI, Facial Appearance Inventory; MAS, Medication Attribution Scale; HIVTSQ, HIV Treatment Satisfaction Questionnaire; TES, Treatment-related Empowerment Scale; SIS, Subcutaneous Injection Survey; QUOTE-HIV, Quality of care through the patient’s eyes; AHHCP, Attitudes toward HIV Health Care Provider scale; AGAS, Antiretroviral General Adherence Scale; HCR, Health Care Relationship Trust Scale; HMRS, HIV Medication Readiness Scale; HTOS, HIV Treatment Optimism Scale; HIV-MT-SES, HIV Medication Taking Self-Efficacy Scale; BEHKA-HIV, Brief Estimate of Health Knowledge and Action-HIV version; HTRM, HIV Treatment Readiness Measure; HTRFS, HIV Treatment Regimen Fatigue Scale; HECCS, HIV Engagement in and Continuity of Care Scale; SSI, Social Support Inventory; USII-HIV, Unsupportive Social Interactions Inventory –HIV version; PSS-HIV, Perceived Social Support for HIV; HIV-ASES, HIV Treatment Adherence Self-Efficacy Scale; PHIVSMS, Perceived HIV Self-Management Scale; HIV-SMS-W, HIV Self-management Scale (Women); HIV-IM, HIV Intention Measure; HIVESS, HIV Exercise Stereotypes Scale; HSM-SEWS, HIV Symptom Management Self-Efficacy for Women Scale.

Due to there is no generally accepted "gold-standard" for assessing PRO for PLWHA, the criterion validity of all the study was not assessed.

Results rated as: “+” = positive rating; “?” = indeterminate rating; “±” = inconsistent rating; “-” = negative rating; “empty boxes” = not reported.

Overall Results of PROMs rated as: “+” = sufficient; “?” = indeterminate; “±” = inconsistent; “-” = insufficient; “empty boxes” = not reported.

LoE rated as: “H” = High; “M” = Moderate; “L” = Low; “VL” = Very low; “empty boxes” = not reported.

^a^ The internal consistency can be rated as "sufficient" at least low evidence for “sufficient” structural validity AND Cronbach’s alpha(s) ≥ 0.70 for each unidimensional scale or subscale, the evidence for “sufficient” structural validity may come from different studies, and the “at least low evidence” was defined by grading the evidence according to the GRADE approach.

^b^ The results of all included records should be taken together and it should then be decided if 75% of the results are in accordance with the hypotheses.

## Table S8. PRISMA 2020 Abstract Checklist

| **Section and Topic** | **Item #** | **Checklist item** | **Reported?** |
| --- | --- | --- | --- |
| **TITLE** | | |  |
| **Title** | 1 | Identify the report as a systematic review. | Yes |
| **BACKGROUND** | | |  |
| **Objectives** | 2 | Provide an explicit statement of the main objective(s) or question(s) the review addresses. | Yes |
| **METHODS** | | |  |
| **Eligibility criteria** | 3 | Specify the inclusion and exclusion criteria for the review. | Yes |
| **Information sources** | 4 | Specify the information sources (e.g. databases, registers) used to identify studies and the date when each was last searched. | Yes |
| **Risk of bias** | 5 | Specify the methods used to assess risk of bias in the included studies. | Yes |
| **Synthesis of results** | 6 | Specify the methods used to present and synthesise results. | Yes |
| **RESULTS** | | |  |
| **Included studies** | 7 | Give the total number of included studies and participants and summarise relevant characteristics of studies. | Yes |
| **Synthesis of results** | 8 | Present results for main outcomes, preferably indicating the number of included studies and participants for each. If meta-analysis was done, report the summary estimate and confidence/credible interval. If comparing groups, indicate the direction of the effect (i.e. which group is favoured). | Yes |
| **DISCUSSION** | | |  |
| **Limitations of evidence** | 9 | Provide a brief summary of the limitations of the evidence included in the review (e.g. study risk of bias, inconsistency and imprecision). | Yes |
| **Interpretation** | 10 | Provide a general interpretation of the results and important implications. | Yes |
| **OTHER** | | |  |
| **Funding** | 11 | Specify the primary source of funding for the review. | Yes |
| **Registration** | 12 | Provide the register name and registration number. | No |

*From:* Page MJ, McKenzie JE, Bossuyt PM, Boutron I, Hoffmann TC, Mulrow CD, et al. The PRISMA 2020 statement: an updated guideline for reporting systematic reviews. BMJ 2021;372:n71. doi: 10.1136/bmj.n71. For more information, visit: <http://www.prisma-statement.org/>

## Table S9. PRISMA 2020 Main Checklist

| **Section and Topic** | **Item #** | **Checklist item** | **Location where item is reported** |
| --- | --- | --- | --- |
| **TITLE** | | |  |
| **Title** | 1 | Identify the report as a systematic review. | p.1 |
| **ABSTRACT** | | |  |
| **Abstract** | 2 | See the PRISMA 2020 for Abstracts checklist. | Table S7 |
| **INTRODUCTION** | | |  |
| **Rationale** | 3 | Describe the rationale for the review in the context of existing knowledge. | p.4-5 |
| **Objectives** | 4 | Provide an explicit statement of the objective(s) or question(s) the review addresses. | p.5 |
| **METHODS** | | |  |
| **Eligibility criteria** | 5 | Specify the inclusion and exclusion criteria for the review and how studies were grouped for the syntheses. | p.6-7 |
| **Information sources** | 6 | Specify all databases, registers, websites, organisations, reference lists and other sources searched or consulted to identify studies. Specify the date when each source was last searched or consulted. | p.6 |
| **Search strategy** | 7 | Present the full search strategies for all databases, registers and websites, including any filters and limits used. | p.6; Table S2 |
| **Selection process** | 8 | Specify the methods used to decide whether a study met the inclusion criteria of the review, including how many reviewers screened each record and each report retrieved, whether they worked independently, and if applicable, details of automation tools used in the process. | p.6-7 |
| **Data collection process** | 9 | Specify the methods used to collect data from reports, including how many reviewers collected data from each report, whether they worked independently, any processes for obtaining or confirming data from study investigators, and if applicable, details of automation tools used in the process. | p.7 |
| **Data items** | 10a | List and define all outcomes for which data were sought. Specify whether all results that were compatible with each outcome domain in each study were sought (e.g. for all measures, time points, analyses), and if not, the methods used to decide which results to collect. | p.7; Table 1; Table S3; Table S4; Table S5; Table S6; |
|  | 10b | List and define all other variables for which data were sought (e.g. participant and intervention characteristics, funding sources). Describe any assumptions made about any missing or unclear information. | p.7; Table 1; Table S3; Table S4; Table S5; Table S6; |
| **Study risk of bias assessment** | 11 | Specify the methods used to assess risk of bias in the included studies, including details of the tool(s) used, how many reviewers assessed each study and whether they worked independently, and if applicable, details of automation tools used in the process. | p.7 |
| **Effect measures** | 12 | Specify for each outcome the effect measure(s) (e.g. risk ratio, mean difference) used in the synthesis or presentation of results. | N/A |
| **Synthesis methods** | 13a | Describe the processes used to decide which studies were eligible for each synthesis (e.g. tabulating the study intervention characteristics and comparing against the planned groups for each synthesis (item #5)). | Figure 1 |
|  | 13b | Describe any methods required to prepare the data for presentation or synthesis, such as handling of missing summary statistics, or data conversions. | N/A |
|  | 13c | Describe any methods used to tabulate or visually display results of individual studies and syntheses. | Table S3 |
|  | 13d | Describe any methods used to synthesize results and provide a rationale for the choice(s). If meta-analysis was performed, describe the model(s), method(s) to identify the presence and extent of statistical heterogeneity, and software package(s) used. | N/A |
|  | 13e | Describe any methods used to explore possible causes of heterogeneity among study results (e.g. subgroup analysis, meta-regression). | N/A |
|  | 13f | Describe any sensitivity analyses conducted to assess robustness of the synthesized results. | N/A |
| **Reporting bias assessment** | 14 | Describe any methods used to assess risk of bias due to missing results in a synthesis (arising from reporting biases). | N/A |
| **Certainty assessment** | 15 | Describe any methods used to assess certainty (or confidence) in the body of evidence for an outcome. | N/A |
| **RESULTS** | | |  |
| **Study selection** | 16a | Describe the results of the search and selection process, from the number of records identified in the search to the number of studies included in the review, ideally using a flow diagram. | p.8 |
|  | 16b | Cite studies that might appear to meet the inclusion criteria, but which were excluded, and explain why they were excluded. | Figure 1 |
| **Study characteristics** | 17 | Cite each included study and present its characteristics. | p.10; p.29; Table 1; Table S3 |
| **Risk of bias in studies** | 18 | Present assessments of risk of bias for each included study. | p.29; Table S4 |
| **Results of individual studies** | 19 | For all outcomes, present, for each study: (a) summary statistics for each group (where appropriate) and (b) an effect estimate and its precision (e.g. confidence/credible interval), ideally using structured tables or plots. | p.29-30; Table S5 |
| **Results of syntheses** | 20a | For each synthesis, briefly summarise the characteristics and risk of bias among contributing studies. | p.30 |
|  | 20b | Present results of all statistical syntheses conducted. If meta-analysis was done, present for each the summary estimate and its precision (e.g. confidence/credible interval) and measures of statistical heterogeneity. If comparing groups, describe the direction of the effect. | Table 2 |
|  | 20c | Present results of all investigations of possible causes of heterogeneity among study results. | N/A |
|  | 20d | Present results of all sensitivity analyses conducted to assess the robustness of the synthesized results. | N/A |
| **Reporting biases** | 21 | Present assessments of risk of bias due to missing results (arising from reporting biases) for each synthesis assessed. | N/A |
| **Certainty of evidence** | 22 | Present assessments of certainty (or confidence) in the body of evidence for each outcome assessed. | N/A |
| **DISCUSSION** | | |  |
| **Discussion** | 23a | Provide a general interpretation of the results in the context of other evidence. | p.36-38 |
|  | 23b | Discuss any limitations of the evidence included in the review. | p.38-39 |
|  | 23c | Discuss any limitations of the review processes used. | p.38-39 |
|  | 23d | Discuss implications of the results for practice, policy, and future research. | p.38-40 |
| **OTHER INFORMATION** | | |  |
| **Registration and protocol** | 24a | Provide registration information for the review, including register name and registration number, or state that the review was not registered. | N/A |
|  | 24b | Indicate where the review protocol can be accessed, or state that a protocol was not prepared. | N/A |
|  | 24c | Describe and explain any amendments to information provided at registration or in the protocol. | N/A |
| **Support** | 25 | Describe sources of financial or non-financial support for the review, and the role of the funders or sponsors in the review. | p.41 |
| **Competing interests** | 26 | Declare any competing interests of review authors. | p.41 |
| **Availability of data, code and other materials** | 27 | Report which of the following are publicly available and where they can be found: template data collection forms; data extracted from included studies; data used for all analyses; analytic code; any other materials used in the review. | N/A |

*From:*  Page MJ, McKenzie JE, Bossuyt PM, Boutron I, Hoffmann TC, Mulrow CD, et al. The PRISMA 2020 statement: an updated guideline for reporting systematic reviews. BMJ 2021;372:n71. doi: 10.1136/bmj.n71. For more information, visit: <http://www.prisma-statement.org/>
